# Directed Brain Connectivity Identifies Widespread Functional Network Abnormalities in Parkinson’s Disease

**DOI:** 10.1093/cercor/bhab237

**Published:** 2021-07-30

**Authors:** Mite Mijalkov, Giovanni Volpe, Joana B Pereira

**Keywords:** anti symmetric correlations, directed networks, functional MRI, network analysis, Parkinson's disease

## Abstract

Parkinson’s disease (PD) is a neurodegenerative disorder characterized by topological abnormalities in large-scale functional brain networks, which are commonly analyzed using undirected correlations in the activation signals between brain regions. This approach assumes simultaneous activation of brain regions, despite previous evidence showing that brain activation entails causality, with signals being typically generated in one region and then propagated to other ones. To address this limitation, here, we developed a new method to assess whole-brain directed functional connectivity in participants with PD and healthy controls using antisymmetric delayed correlations, which capture better this underlying causality. Our results show that whole-brain directed connectivity, computed on functional magnetic resonance imaging data, identifies widespread differences in the functional networks of PD participants compared with controls, in contrast to undirected methods. These differences are characterized by increased global efficiency, clustering, and transitivity combined with lower modularity. Moreover, directed connectivity patterns in the precuneus, thalamus, and cerebellum were associated with motor, executive, and memory deficits in PD participants. Altogether, these findings suggest that directional brain connectivity is more sensitive to functional network differences occurring in PD compared with standard methods, opening new opportunities for brain connectivity analysis and development of new markers to track PD progression.

## Introduction

Parkinson’s disease (PD) is a complex neurodegenerative disorder characterized by a wide range of motor and nonmotor symptoms such as memory, executive, visuospatial, or olfactory deficits (Chaudhuri et al. 2006; Jankovic 2008). The presence of such diverging symptoms suggests that the brain changes occurring in PD cannot be directly linked to the dysfunction of a single brain region but rather to widespread changes in functional connectivity between many regions or brain networks (Pievani et al. 2011).

Functional connectivity can be measured using functional magnetic resonance imaging (MRI), a noninvasive technique that detects changes in blood oxygen level-dependent signals, which are considered to reflect the underlying neuronal brain activity (Biswal et al. 1995). In participants with PD, several studies have shown that motor and nonmotor symptoms can arise due to the loss of integrity in these functional connections (Tahmasian et al. 2015; Gao and Wu 2016). In particular, abnormal functional connectivity in the basal ganglia—thalamocortical network (Blandini et al. 2000; Helmich et al. 2010; Baudrexel et al. 2011) has been linked to motor symptoms in PD, whereas changes in the default mode, dorsal-attention, fronto-parietal, salience, and associative visual networks (van Eimeren et al. 2009; Tessitore et al. 2012; Amboni et al. 2015; Baggio et al. 2015b; Gorges et al. 2015; Putcha et al. 2015; Gao and Wu 2016) have been shown to correlate with cognitive deficits in these participants.

In the past few years, several studies have used functional MRI to assess the functional brain connectome, a whole-brain network that summarizes the complete set of pairwise functional connections in the brain (Biswal et al. 2010). This network consists of a set of nodes, or brain regions, connected by edges, representing the strength of the functional connections. This connectivity network can then be analyzed using graph theory by computing several global and local measures that reflect whether brain regions are efficiently connected by short network paths (global efficiency) or are well integrated into their neighborhood (clustering) or community (modularity) (Rubinov and Sporns 2010). These analyses have shown significant changes in the global efficiency, local efficiency, and clustering coefficient in the whole brain (Göttlich et al. 2013; Baggio et al. 2014) or within specific networks in PD participants (Wei et al. 2014; Koshimori et al. 2016; Maidan et al. 2019). Changes in the nodal network topology of prefrontal and supplementary motor areas as well as the striatum and thalamus (Wu et al. 2009; Sang et al. 2015; Fang et al. 2017; Lopes et al. 2017) have also been reported in PD, sometimes in association with clinical measures (Lebedev et al. 2014; Sreenivasan et al. 2019).

Despite being useful to assess network changes in PD, these studies were based on the assumption that brain activity in different brain regions occurs simultaneously and, therefore, can be captured by same-time undirected correlations in the activation signals between them. As such, they do not convey information about the directionality of the interaction between brain regions (Friston 2011), which is important due to an increasing number of studies showing that directed brain activity patterns are altered in PD. These directed patterns have been assessed using dynamic causal modeling (Rowe et al. 2010; Kahan et al. 2014), structural equation modeling (Rowe et al. 2002; Palmer et al. 2009), psycho–physiological interactions (Wu et al. 2011), or Granger causality (Wu et al. 2012; Ghasemi and Mahloojifar 2013) methods. Due to the complex nature and longer computational time required by these methods, their application is currently limited to the assessment of brain connectivity between a few regions or to the analysis of functional MRI data acquired during a specific task, which normally relies on a priori hypotheses of which brain regions should be tested. Moreover, several generalizations for the assessment of directed whole-brain connectivity have also been recently proposed (Razi et al. 2017; Frässle et al. 2018; Seguin et al. 2019; Gilson et al. 2020; Prando et al. 2020; Frässle et al. 2021). However, these methods are still constrained by their computational efficiency and identifiability (Frässle et al. 2021). Moreover, their application to study functional networks to assess functional changes in neurodegenerative diseases has not been systematically evaluated.

Here, we present an intuitive and computationally light method to assess resting-state, whole-brain directed functional networks based on antisymmetric lagged correlations. First, we obtain a lagged correlation adjacency matrix for each participant by calculating the pairwise lagged correlations between all pairs of brain regions. Then, the antisymmetric correlations are derived as the antisymmetric part of the lagged correlation adjacency matrix. We demonstrate that the topological organization of these functional networks is more sensitive to pathological changes related to PD when compared with functional networks built by standard undirected methods.

## Materials and Methods

### Construction of Directed Functional Networks

Activation signals are typically generated in one brain region and then propagated to other ones (Hammond 2015), which entails causality and lags in the activation of various brain regions. Such temporal lags can also arise, for example, due to the spatial distribution of brain regions and the finite transmission speeds between them (Deco et al. 2009). Therefore, capturing the information stored in this complex temporal lag framework is necessary to achieve a coherent characterization of functional connectivity (Lahaye et al. 2003; Jafri et al. 2008; Mijalkov et al. 2020). In this work, we harvest this additional information by calculating directed functional connectivity between brain regions using lagged Pearson’s correlations. In this approach, a brain region is considered to have a directed interaction with other brain regions if its activation time series has similar properties with the time-shifted version of the second brain region’s activation pattern. Moreover, brain regions that are more closely connected to each other are expected to activate with a much shorter delay than regions that are more indirectly connected (Ghosh et al. 2008; Deco et al. 2009). Building on the assumption that quasi-simultaneous brain activity primarily occurs between nodes connected by direct paths, we can interpret the different lags in the activation patterns between brain regions as an indicator of the topological connectivity distance between them. For example, connectivity networks at small temporal lags represent brain regions connected with direct connections, while larger temporal lags capture the network of regions connected via indirect connections of various lengths. Therefore, in order to explore the functional activation patterns of the brain at these different scales of topological connectivity, we assessed directed functional connectivity at multiple temporal lags (“Methods: Lagged correlation”).

**Figure 1:**
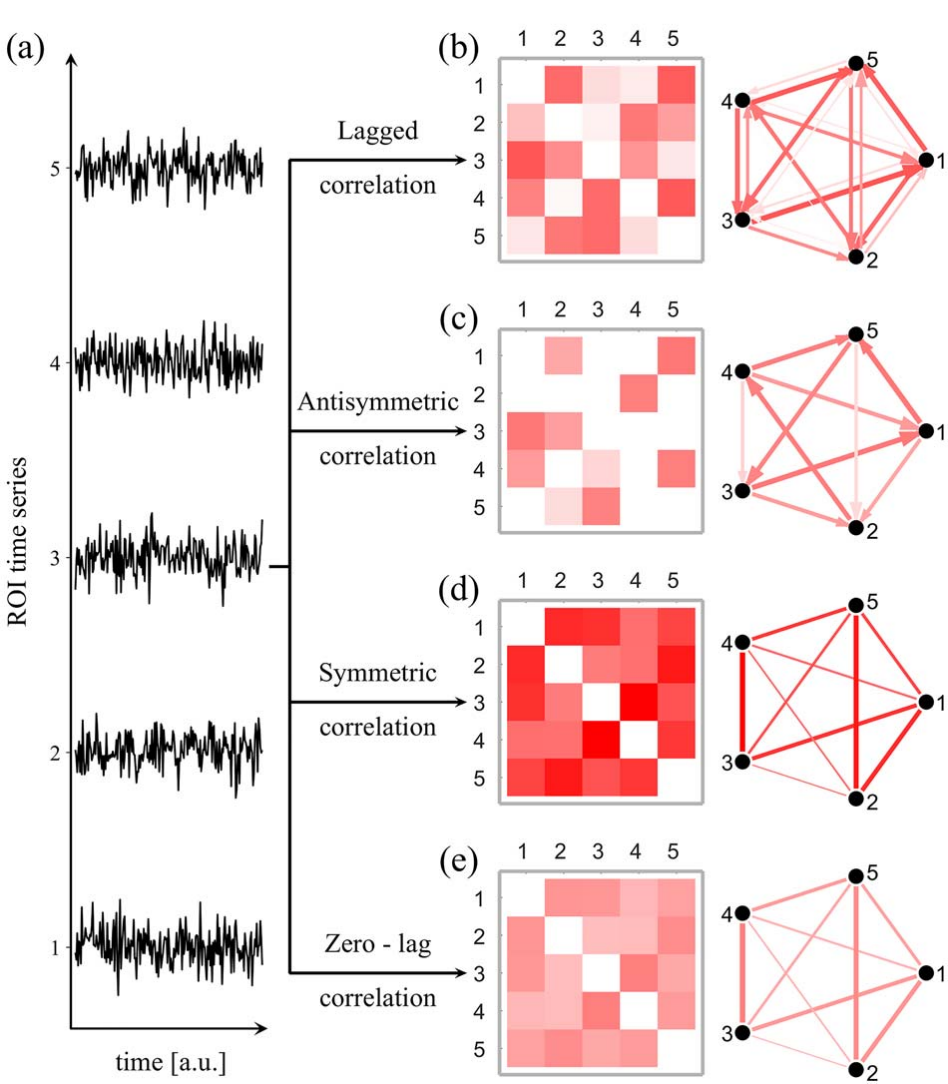
Different methods used to calculate functional networks. (*a*) For illustration purposes, we show an example of the time activation series of only five nodes. (*b*) Lagged correlation functional networks can be estimated by calculating the lagged Pearson’s correlation coefficient between these time series, at different lags. Here, the lagged adjacency matrix and corresponding network are calculated at lag of 1. The lagged adjacency matrix can be written as a sum of (*c*) anti-symmetric and (*d*) symmetric matrices. Finally, for comparison, we show the commonly used method of zero-lag correlation (*e*). In all matrices, redder colors and thicker lines indicate stronger connections.

Figure 1 illustrates the different methods we used to calculate the functional connectivity networks for a set of five brain regions and their activation time series (Fig. 1*a*). The connectivity matrix and the corresponding network calculated by the lagged correlation adjacency method for these five brain regions are shown in Figure 1*b*: the lagged correlation method evaluates the directed connection between two regions in both directions; a pair of elements in the lagged adjacency matrix (namely, *(i*, *j)* and *(j*, *i)*) provides an estimate of the directed relation from brain region *i* to brain region *j* and vice versa. As this is a correlation-based measure, it does not attempt to evaluate the “effective connectivity” between two brain regions (Friston 2011). Instead, we use it to quantify the directed functional connectivity between the two regions, with the direction depending on the temporal precedence (i.e., the early region is the source, and the late region is the end of the connection).

As any other square matrix, the lagged correlation adjacency matrix can be uniquely expressed as the sum of a symmetric and antisymmetric matrix. Specifically, the antisymmetric matrix captures the directionality of the functional network, identifying the relevant directed connections between the couples of brain regions (Fig. 1*c*). We call this method “antisymmetric correlation” (“Methods: Anti-symmetric and symmetric correlations”).

To highlight the effectiveness of the directed networks in detecting topological changes between controls and participants with PD, we compare our method with two undirected network approaches. In the first approach, functional connectivity is evaluated as the symmetric matrix extracted from the lagged correlation adjacency matrix (Fig. 1*d*), in which the undirected connection between two regions is the sum of the weights of the two corresponding directed connections (“Methods: Anti-symmetric and symmetric correlations”). Second, we also compare our method with the conventional approach to quantify functional connectivity, in which the connectivity strength between two regions is estimated by calculating the zero-lag Pearson’s correlation coefficient (Fig. 1*e*) between their activation time series (“Methods: Zero-lag correlation”). While these two methods are identical when the symmetric correlation is calculated at lag 0 and show a very high correlation at small lags (Supplementary Fig. 1), the correlation between the two methods decreases with the increase of the temporal lag. This indicates that the symmetric correlation captures different scales of undirected connectivity as a function of the temporal lag, thus providing an appropriate framework to compare the behavior of directed and undirected methods at different temporal lags. Since these different connectivity scales cannot be efficiently captured by the zero-lag correlation, the agreement between the two methods decreases for high temporal lags.

We tested the ability of all four methods to detect topological changes in 95 participants with PD compared with 15 controls with functional MRI data from the Parkinson’s Progression Markers Initiative (“Methods: Participants”) (Marek et al. 2011). For up-to-date information on the study, visit www.ppmi-info.org. The nodes in the adjacency matrices corresponded to the 200 brain regions derived from the Craddock atlas (Craddock et al. 2012), while the edges were calculated according to the four methods described above, yielding four different weighted adjacency matrices for each participant. For each adjacency matrix, we calculated a binary matrix where the correlation coefficient was considered 1 if it was above a certain threshold, and 0 if it was below. As there are multiple other thresholding approaches and, currently, there is no consensus as to which network density should be used (Fornito et al. 2013), we performed the thresholding at the complete available range of network densities (*D*) of the antisymmetric correlation network (${D}_{\mathrm{min}}=1\%$ to ${D}_{\mathrm{max}}=50\%$ in steps of $1\%$) and we compared the network topologies across that range. In addition, we also compared our results with the ones obtained with an alternative weighted analysis approach, in which the weight of the individual edges was retained after the binarization of the network. The negative correlation coefficients and self-connections were excluded from all analyses by setting them to zero.

### Lagged Correlation

The lagged correlation between the activation time series of two brain regions ($j$ and $k$) with activation time courses ${x}_j$ and ${x}_k,$ respectively, is calculated as the Pearson’s correlation coefficient between ${x}_j$ and lagged versions of ${x}_k$ evaluated as a function of the temporal lag. The lag is the number of repetition times by which ${x}_k$ is shifted with respect to ${x}_j$ before calculating the correlation. Therefore, the strength of the functional connectivity between the brain regions $j$ and $k$ at a given delay *d* is calculated as

$$ {\rho}_{j\to k}(d)=\frac{1}{N-d-1}\ \sum_{i=1}^{N-d}\left(\frac{x_j^{\prime }-\mu \left({x}_j^{\prime}\right)}{\sigma \left({x}_j^{\prime}\right)}\right)\ \left(\frac{x_k^{\prime }-\mu \left({x}_k^{\prime}\right)}{\sigma \left({x}_k^{\prime}\right)}\right), $$

where $N$ is the total number of measurements, ${x}_j^{\prime }$ represents the first $N-d$ measurements of ${x}_j$, ${x}_k^{\prime }$represents the last $N-d$ measurements of ${x}_k$, $\mu ({x}_j^{\prime})$ and $\sigma ({x}_j^{\prime})$ are the mean and standard deviation (SD) of ${x}_j^{\prime }$, respectively, and $\mu ({x}_k^{\prime})$ and $\sigma ({x}_k^{\prime})$ are the mean and SD of ${x}_k^{\prime }$. In this construction, ${x}_k$ is shifted by $d$ time steps with respect to ${x}_j$; therefore, the correlation coefficient ${\rho}_{j\to k}(d)$ is an estimation of the directed functional connectivity from region $j$ to region $k$ due to temporal precedence. By repeating this calculation for all pairs of nodes, we obtain the weighted directed lagged correlation functional network. This network was subsequently binarized at the specified range of densities in order to compare network topologies between the two groups. In this matrix, the directed connection between a pair of nodes $j$ and $k$ is represented by a pair of elements $(j,k)$ and $(k,j)$ that quantify the estimated directed connection from brain region $j$ to brain region $k$ and vice versa.

### Antisymmetric and Symmetric Correlations

Being a square matrix, the lagged correlation matrix calculated as outlined above can be written as a sum of univocally defined symmetric and antisymmetric matrices. Therefore, from the lagged correlation matrix $L$, one can calculate the corresponding antisymmetric matrix $A$ as

$$ A=L-{L}^{\mathrm{T}}, $$

where ${L}^{\mathrm{T}}$ denotes the transpose of $L$. As described previously, all negative connections are set to zero. Calculated in this way, the antisymmetric analysis represents any directed correlation between two regions $j$ and $k$ with a single entry in the adjacency matrix, which summarizes both the direction and the magnitude of the directed influence.

The symmetric matrix can be calculated as

$$ S=L+{L}^{\mathrm{T}}. $$

Symmetric matrices do not convey any information about the direction of the functional connections. The magnitude of a connection is calculated as the sum of the connection weights in the directed connections between nodes that run in both directions. The advantage of this method when compared with the zero-lag correlation method is that it can be evaluated at various temporal lags, therefore allowing a more direct comparison with the corresponding directed methods.

### Zero-Lag Correlation

In the standard zero-lag correlation method, the functional connectivity between two nodes $j$ and $k$ with respective activation time series ${x}_j$ and ${x}_k$ is quantified by the Pearson’s linear correlation coefficient at lag of $0$, calculated as

$$ {\rho}_{jk}={$\operatorname{cov}\left({x}_j,{x}_k\right)$}\!\left/ \!{$\left({\sigma}_j{\sigma}_k\right)$}\right., $$

where $\operatorname{cov}({x}_j,{x}_k)$ represents the covariance of the corresponding activation time series and ${\sigma}_j$ and ${\sigma}_k$ are their respective SDs. The functional networks are built by calculating the Pearson’s coefficient between all pairs of nodes in the network.

### Granger Causality

To compare the antisymmetric correlation method to alternative methods of directed functional connectivity, we also calculated whole-brain functional networks in controls and PD participants using Granger causality. The Granger causality was evaluated using the “Granger causal connectivity analysis” toolbox as described in Seth (2010). Granger causality is defined within the context of autoregressive linear models and it assumes that the behavior of two time series, ${x}_1(t)$ and ${x}_2(t)$, can be fitted to a bivariate autoregressive model

$$ {x}_1(t)=\sum_{j=1}^p{\mathrm{A}}_{11,j}{x}_1\left(t-j\right)+\sum_{j=1}^p{\mathrm{A}}_{12}{x}_2\left(t-j\right)+{\epsilon}_1(t) $$

$$ {x}_2(t)=\sum_{j=1}^p{\mathrm{A}}_{21,j}{x}_1\left(t-j\right)+\sum_{j=1}^p{\mathrm{A}}_{22}{x}_2\left(t-j\right)+{\epsilon}_1(t), $$

where $p$ is the model order (i.e., maximum number of lags that are included in the model), ${\epsilon}_1$ and ${\epsilon}_2$ are the residuals of the corresponding time series, and $\mathrm{A}$ is a matrix of the estimated coefficients of the model. In this linear regression model, ${x}_2(t)$ is considered to “cause” ${x}_1(t)$ if the addition of the past values of ${x}_2(t)$ in the model of ${x}_1(t)$ reduces the variance of the prediction error${\epsilon}_1$, when compared with a model that includes only the previous observations of ${x}_1(t)$. Then, assuming that ${x}_1(t)$ and ${x}_2(t)$ are covariance stationary (they have unchanging mean and variance), the magnitude of the interaction is estimated by

$$ {F}_{2\to 1}=\ln\ \left(\frac{\operatorname{var}\left({\epsilon}_{1\mathrm{R}}\right)}{\operatorname{var}\left({\epsilon}_{1\mathrm{U}}\right)}\right), $$

where ${\epsilon}_{1\mathrm{R}}$ is derived from the model, ${x}_1(t)$ is predicted only from its past values (i.e., by omitting ${\mathrm{A}}_{12}$ from the first equation for all coefficients $j$), and ${\epsilon}_{1\mathrm{U}}$ is derived from the full model. We obtained the directed networks by evaluating the pairwise $F$-statistic for all pairs of brain regions.

### Participants

Demographic and clinical characteristics of the participants are shown in Table 1. At baseline, participants with PD met the standard diagnostic criteria for PD, were diagnosed within 2 years of the screening visit, were entirely untreated, had an Hoehn and Yahr (Hoehn and Yahr 1967) stage of I or II, and were required to have a dopamine transporter deficit on DaTSCAN imaging for neurobiological confirmation of a PD diagnosis. Inclusion criteria for healthy controls consisted of not having neurologic dysfunction, no first-degree family member with PD, and a Montreal Cognitive Assessment (MoCA) score > 26. Motor symptoms were assessed using the unified Parkinson’s disease rating scale (UPDRS) and olfactory function was evaluated using the smell identification test (UPSIT). In addition, all participants completed several cognitive tests that assessed visuospatial functions (15-item version of the Benton’s judgment of line orientation test), verbal memory (immediate recall and delayed recall of the Hopkins verbal learning test-revised, HVLT-R), executive functions (the letter number sequencing test, semantic and phonemic fluency tests), and attention (symbol digit modalities test, SDMT). The total levodopa-equivalent doses were calculated for all participants with PD. The cognitive and motor assessments were performed by PD participants while on medication. The classification of MCI was performed according to the guidelines of the MDS Task Force for the level II diagnosis of PD-MCI (Litvan et al. 2012). Participants were classified as having MCI if they showed impairment in 2 or more tests or items within the same cognitive domain or in 2 or more domains. Impairment was defined as a score below 2.0 SD for the individual continuous tests, or a score below the maximum for the ordinal and categorical items, based on previous recommendations made by the MDS Task Force criteria for PD dementia (Dubois et al. 2007) and similarly to our previous studies (Pereira et al. 2012, 2014).

**Table 1:** Characteristics of the sample

	CTR (*n* = 15)	PD (*n* = 95)	CTR versus PD (*P*-value)
Age (years)	72.1 (8.3)	68.0 (10.5)	0.15
Sex (%male)	86.7%	68.4%	0.13
Education (years)	16.7 (2.3)	15.3 (2.9)	0.06
UPDRS-III scores	1.2 (1.4)	21.3 (10.7)	<0.001
HY stage (1–2)	—	68–27	—
LEDD (%medicated)	—	67.4%	—
LEDD (dose)	—	405.3 (207.0)	—
Cognitive status (%MCI)	—	20%	—
UPSIT	35.2 (3.14)	21.61 (8.63)	<0.001
HVLT-R Immediate Recall	8.8 (2.51)	8.55 (2.95)	0.77
HVLT-R Delayed Recall	11.60 (0.83)	11.20 (1.77)	0.44
Benton’s judgment of line orientation test	12.47 (2.17)	12.80 (1.81)	0.55
LNS	10.47 (2.85)	10.25 (2.71)	0.80
MoCA	27.67 (1.40)	26.88 (2.80)	0.30
SDMT	45.80 (10.35)	40.01 (11.03)	0.07
Semantic fluency	51.80 (10.60)	49.47 (11.01)	0.46

Notes: Means are followed by SD in parenthesis. Permutation tests with 10 000 permutations were used to compare groups for age, sex, education, LEDD dose, Hoehn and Yahr stage, and different test scores. CTR, controls; UPDRS-III, Unified Parkinson’s disease rating scale–Part III; HY stage, Hoehn and Yahr stage; LEDD, levodopa equivalent dose; UPSIT, smell identification test; HVLT-R TR and HVLT-R DR, total immediate recall and delayed recall of the Hopkins verbal learning test-revised; Benton, Benton’s judgment of line orientation test; LNS, letter number sequencing test; SF, semantic fluency tests; MoCA, Montreal Cognitive Assessment.

### Image Acquisition

All participants were scanned on a 3 Tesla Siemens scanner using an echo planar functional MRI sequence with the following parameters: 212 time points, repetition time = 2400 ms, echo time = 25 ms, field of view = 222 mm, flip angle = 80°, and 3.3-mm isotropic voxels. During the scanning session, participants were instructed to keep their eyes open, rest quietly and to not fall asleep.

### Image Preprocessing

All images were preprocessed using the statistical parametric mapping software (SPM12, https://www.fil.ion.ucl.ac.uk/spm/). Briefly, after removing the first 5 volumes, all images were realigned and slice-time corrected. Then, the six rigid motion parameters as well as the white matter and cerebrospinal fluid signals were regressed from all images, which were subsequently normalized to MNI space and band-pass filtered (0.01–0.08 Hz). The mean time series of each brain region included in the 200-node Craddock atlas were extracted for each participant. Only participants with a functional MRI scan that passed quality control before and after image preprocessing were included; in particular, we only included participants whose motion parameters did not exceed a single voxel size of 3 mm.

### Definition of Graph Measures

All graph measures were calculated using the Brain Analysis using Graph Theory software (http://braph.org/) (Mijalkov et al. 2017). In the case of directed binary networks, the in-degree of a node is defined as the number of inward edges going into a node. The out-degree of a node is the number of outward edges originating from a node. Denoting the network adjacency matrix with $A$ and its elements as ${a}_{ij}$, the in- and out-degrees of a node *i* are expressed as

$$ {d}_i^{\mathrm{in}}=\sum_{j\ne i}{a}_{ji}, $$

$$ {d}_i^{\mathrm{out}}=\sum_{j\ne i}{a}_{ij}. $$

The degree of a node is expressed as the sum of the node’s respective in- and out-degrees

$$ {d}_i={d}_i^{\mathrm{in}}+{d}_i^{\mathrm{out}}\ . $$

A direct path between two nodes $i$ and $j$ is the sequence of directed edges that need to be traversed in order to reach $j$ starting from $i$. The directed distance $\overrightarrow{D_{ij}}$ is the number of edges contained in the shortest directed path from $i$ to $j$. For a given node $i$, ${D}_{\mathrm{max}}(i)$ can be defined as the maximal distance between $i$ and any other node. Then, the network diameter, ${D}_{\mathrm{ntw}}$, is defined as the largest maximal distance of all nodes, expressed as

$$ {D}_{\mathrm{ntw}}=\max \left(\ {D}_{\mathrm{max}}(i),{D}_{\mathrm{max}}(j),{D}_{\mathrm{max}}(k)\dots \dots \right), $$

where the maximization is performed over the maximal distances of all nodes in the network.

The regional out-global efficiency of a node $i$, denoted by ${e}_{\mathrm{out}}(i)$, is defined as the average inverse distance from $i$ to all other nodes in the network, when considering only directed paths originating from $i$. Analogously, the regional in-global efficiency of node $i$, ${e}_{\mathrm{in}}(i),$ is the average of the inverse distance to $i$ from all other nodes in the network over directed paths ending at $i$. The global counterparts of these measures in a network with $N$ nodes can be calculated as the average of the regional out- and in-efficiency of all nodes

$$ {E}_{\mathrm{in}}=\frac{1}{N}\sum_{i\in N}{e}_{\mathrm{in}}(i)=\frac{1}{N}\sum_{i\in N}\frac{\sum_{j\in N,j\ne i}\overrightarrow{D_{ji}^{-1}}}{n-1}, $$

$$ {E}_{\mathrm{out}}=\frac{1}{N}\sum_{i\in N}{e}_{\mathrm{out}}(i)=\frac{1}{N}\sum_{i\in N}\frac{\sum_{j\in N,j\ne i}\overrightarrow{D_{ij}^{-1}}}{n-1}. $$

We furthermore calculated the regional in- and out- local efficiency of a node $i$ defined as the corresponding global efficiency measure evaluated on the subgraph consisting of nodes that are neighbors of $i$. The in- and out-local efficiency of the network, $L{E}_{\mathrm{in}}$ and $L{E}_{\mathrm{out}}$, respectively, is calculated by averaging the corresponding measures over all nodes in the network. We defined the network’s total global efficiency ($E$) and local efficiency ($LE$) as the mean of the in- and out-efficiency measures:

$$ E=\frac{1}{2}\ \left({E}_{\mathrm{in}}+{E}_{\mathrm{out}}\right), $$

$$ LE=\frac{1}{2}\ \left({LE}_{\mathrm{in}}+{LE}_{\mathrm{out}}\right). $$

The clustering coefficient ${C}_i$, of node $i$, reflects the fraction of the neighbors of $i$ that are also connected with each other. It can be calculated as the fraction of completed triangles that are present around $i$. In directed networks, we consider a triangle to be completed if its constituent edges form a cycle in either direction. Therefore, we calculate the clustering coefficient as

$$ {C}_i=\frac{{\left({A}^3\right)}_{ii}}{d_{\mathrm{in}}{d}_{\mathrm{out}}-\overset{\leftrightarrow }{d_i}}, $$

where ${d}_{\mathrm{in}}$ and ${d}_{\mathrm{out}}$ are the in- and out-degree, respectively, and $\overset{\leftrightarrow }{d_i}$ is the number of bilateral edges between *i* and its neighbors

$$ \overset{\leftrightarrow }{d_i}=\sum_{j\ne i}{a}_{ij}{a}_{ji}={A}_{ii}^2. $$

The transitivity indicates the number of triangles present within the complete network. As such, it is calculated as

$$ T=\frac{3\times \mathrm{total}\ \mathrm{number}\ \mathrm{of}\ \mathrm{triangles}\ }{d_{\mathrm{tot}}\left({d}_{\mathrm{tot}}-1\right)-2\times \mathit{\operatorname{diag}}\left({A}^2\right)}, $$

where again we consider a triangle to be completed only if the three directed edges form a cycle, and $\mathit{\operatorname{diag}}({A}^2)$ is the sum of the diagonal elements in the ${A}^2$ matrix.

The modularity quantifies the degree at which a given network can be subdivided into clearly separated communities that have large density of within-community edges and small number of between community edges. Modularity was calculated using Louvain algorithm, using $\gamma =1$ (Blondel et al. 2008). To evaluate the topology of the weighted networks, we have used the generalizations for degree, global efficiency, local efficiency, clustering coefficient, transitivity, and modularity as outlined by Rubinov and Sporns (2010).

### Area under the Curve Analysis

We used AUC analysis to evaluate the differences in the nodal directed connectivity patterns between PD and control groups. This analysis takes into account the complete density range and, therefore, is less sensitive to the thresholding process (Fornito et al. 2013). Each curve represents the changes in the corresponding nodal network measure as a function of the network density for a given brain region. We first obtained an estimate of the AUC by numerically integrating the nodal values over the whole density range; this resulted in a single numerical value for each network measure and each brain region across the range of densities. Then, the between-group differences in the nodal measures were assessed by comparing the corresponding AUC values for all brain regions.

### Statistical Analysis

The statistical significance of the differences between PD participants and controls was assessed by performing nonparametric permutation tests with 10 000 permutations, which were considered significant for a two-tailed test of the null hypothesis at $p<0.05$. Additionally, we assessed the regional network results by calculating the area under the curve (AUC) for each regional measure across the whole density range; these results were adjusted for multiple comparisons by applying false discovery rate (FDR) corrections at $q<0.05$ using the Benjamini–Hochberg procedure (Benjamini and Hochberg 1995) to control for the number of regions. Nonparametric permutation tests with 10 000 permutations were also used to assess between-group differences in demographic and clinical variables. All analyses included age, sex, and the 6 rigid-body motion parameters as covariates.

## Results

### Average Group Networks Show a Different Behavior across Different Temporal Lags

**Figure 2:**
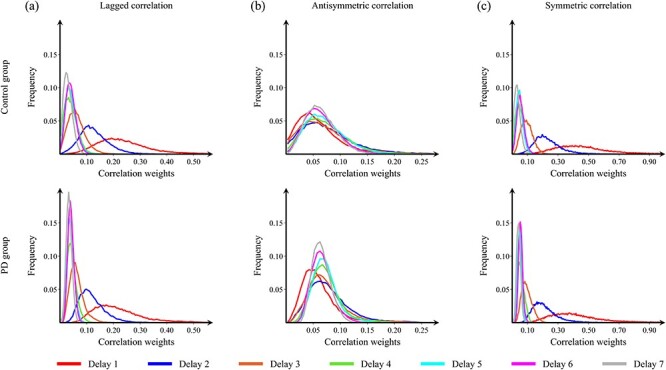
Connectivity strength distribution at different temporal lags. Histograms show the distribution of connectivity strengths of the average adjacency matrices for controls (top row) and PD participants (bottom row) as a function of different temporal lags. The individual connectivity matrices were calculated using (*a*) lagged analyses, (*b*) anti-symmetric analyses, and (*c*) symmetric analyses. Only the lags used in the analysis are shown in this figure.

We calculated group-representative adjacency matrices at different temporal lags by averaging the weighted, participant-specific adjacency matrices. The histograms of the connection weights are shown in Figure 2 for the lagged (Fig. 2*a*), antisymmetric (Fig. 2*b*), and symmetric (Fig. 2*c*) correlations as a function of different temporal lags. Figure 2 shows a general decrease of the strength of directed connectivity in PD participants at all lags when compared with healthy controls. Furthermore, in PD participants, we observed that the connectivity strength distribution becomes narrower with increasing temporal lags for all analyses. This observation indicates that, with higher temporal lags, more nodes have similar functional connectivity strength. Therefore, large temporal lags are unsuitable for the analysis of between-group topological differences because they cannot capture any variations in the directional flow in the network, restricting our analysis to small temporal lags in the range $1\hbox{--} 7$. These results are further supported by the evaluation of network diameter for all participant-specific adjacency matrices at different temporal lags (Supplementary Fig. 2).

### Differences between Groups in Global Network Topology

**Figure 3:**
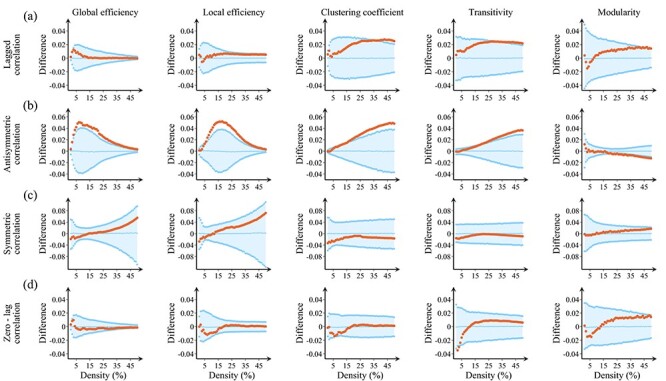
Differences between controls and PD participants in global network measures. Plots showing the differences between controls and participants with PD (calculated as PD group—control group) in the global efficiency, local efficiency, clustering coefficient, transitivity, and modularity using (*a*) lagged correlation, (*b*) anti-symmetric correlation, (*c*) symmetric correlation, and (*d*) zero-lag correlation methods. The plots show the upper and lower bounds of the 95% confidence intervals (CI) in blue, and the differences in the network measures between groups in orange circles as a function of network density. The differences are considered statistically significant if they fall outside the CIs. The results shown were obtained for temporal lag$1$.

To assess the ability of these methods to detect global network changes between participants with PD and controls, we calculated the global efficiency, local efficiency, clustering coefficient, transitivity, and modularity (Fig. 3, left to right columns). The antisymmetric correlation method showed widespread significant differences between PD participants and controls in network measures; this entails that the differences are contained in the antisymmetric part of the lagged correlation matrix. These differences consisted of increases in the clustering coefficient and transitivity in the PD participants compared with controls at higher network densities (clustering coefficient: $16\hbox{--} 50\%$; transitivity: $20\hbox{--} 50\%$). The global and local efficiency also showed differences between PD participants and controls, being increased in PD participants across most network densities (global efficiency: $2\hbox{--} 50\%$; local efficiency: $6\hbox{--} 50\%$). Finally, we also found significant decreases in the modularity in PD participants compared with controls, which were only present at higher network densities ($21\hbox{--} 50\%$). In contrast, the undirected and lagged correlation methods did not show significant differences in any of the global network measures between PD participants and controls. Figure 3 summarizes the results obtained for the temporal lag$1$; the corresponding results for lags $2\hbox{--} 7$ are shown in Supplementary Figures 3–8. The plots of the corresponding measures as a function of density, calculated by the antisymmetric correlation method at various temporal lags, are shown in Supplementary Figures 9 and 10. They show similar patterns for both controls and PD participants and exhibit monotonous changes across the density range, demonstrating that the above differences reflect changes in network topology rather than a potential mismatch of the number of antisymmetric connections in both groups or changes in the overall functional connectivity strength (van den Heuvel et al. 2017).

### Differences between Groups in Nodal Network Topology

**Figure 4:**
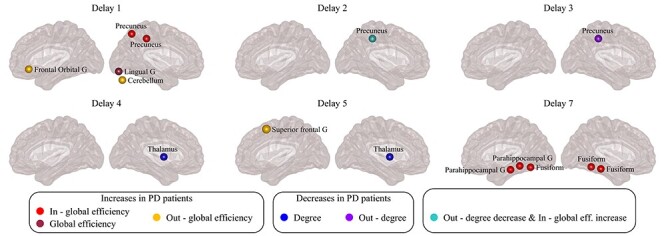
Differences between controls and PD participants in nodal network measures. Visual display of the nodes that show significant differences between controls and participants with PD in network measures using the anti-symmetric correlation method. Differences between groups were evaluated using nonparametric permutation tests. Only regions that show significant differences after correcting for multiple comparisons (FDR at $q=0.05$) are plotted. See also Supplementary Figures 11–16.

Furthermore, using the antisymmetric correlation method, we also identified directed connectivity changes in several brain regions in PD participants compared with controls (Fig. 4, see also Supplementary Figures 11–16 and Supplementary Table 1). Specifically, we found significant increases in the in-global efficiency in the precuneus (at various temporal lags), the fusiform and parahippocampal gyrus (lag 7) as well as significant increases in the global efficiency in the lingual gyrus (lag 1) in PD participants. Moreover, we found increases in the out-global efficiency of the frontal orbital gyrus and cerebellum (lag 1) and in the superior frontal gyrus (lag 5). Finally, we also found decreases in the overall connectivity of the thalamus (lags 4 and 5) and in the outflow connectivity of the precuneus (lag 3) in PD participants. The other three analysis methods were not able to identify any significant between-group differences in nodal measures, even before correcting for multiple comparisons.

### Correlation Analysis with Clinical Measures in PD Participants

All global network measures were significantly associated with the UPDRS-III motor scores and executive scores (Letter-Number sequencing test) across all lags. In addition, the clustering and transitivity also correlated with executive scores (SDMT) at lag $1$, whereas global efficiency correlated with memory (Hopkins verbal learning test) at lag $5$. Global and local efficiency, clustering and transitivity correlated with visuospatial scores (Benton’s judgment of line orientation test) at lag $7$. The best results that remained significant after adjusting for multiple comparisons to control for the different densities (FDR, $q=0.05$) are summarized in Supplementary Material (Supplementary Figs 17–21 and Supplementary Tables 2–18).

Regarding the nodal network measures, we only assessed the correlation between regions showing significant between-group differences (AUC analysis) with clinical measures. After correcting the results for the number of cognitive tests, the out-global efficiency of the cerebellum was significantly associated with UPDRS-III motor scores at lag 1 ($P-\mathrm{value}=0.001$; $r=-0.32$) in PD participants. The out-degree of the precuneus was significantly associated with olfactory function (UPSIT smell identification test) at lag $2$ ($P-\mathrm{value}=0.004$; $r=-0.30$) and the degree of the thalamus correlated with visuospatial scores (Benton’s judgment of line orientation test) at lag $4$ ($P-\mathrm{value}<0.001$; $r=-0.36$) in PD. No correlations were found between global and nodal network measures with clinical measures in the control group.

### Effect of Dopaminergic Medication on Functional Network Topology

**Figure 5:**
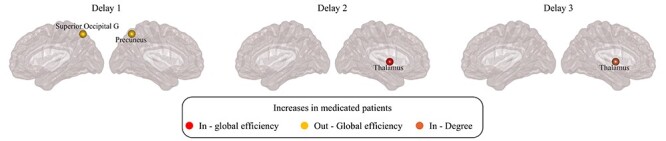
Differences between medicated and nonmedicated participants with PD in nodal network measures. Plots showing the differences between medicated and nonmedicated participants with PD in nodal network measures evaluated using AUC analysis, at different temporal lags, in the case of antisymmetric correlation. Differences between groups were evaluated using nonparametric permutation test. Only regions that show significant differences after correcting for multiple comparisons (FDR at $q=0.05$) are plotted.

To evaluate the effect of levodopa-equivalent doses on functional network organization, we compared the networks of medicated participants to those who were not receiving medication (details about the two subgroups are shown in Supplementary Table 19). We did not find any differences in the global network topology between these groups. Regarding nodal topology, there were significant increases in medicated participants versus nonmedicated ones in the out-global efficiency of precuneus and superior occipital gyrus at lag $1$, and in the in-global efficiency and in-degree of thalamus at lags 2 and 3, respectively (Fig. 5). Of note, none of these results overlapped with the results of the main analyses.

### Influence of Mild Cognitive Impairment on Functional Network Topology

Due to previous evidence showing that PD participants with MCI show more widespread network changes compared with cognitively normal participants (Baggio et al. 2014, Gorges et al. 2015), we performed an additional analysis to compare these two groups (participant characteristics for both subgroups are shown in Supplementary Table 20). Only one significant difference was found in the cerebellum, which showed significant degree decreases in participants with MCI at lag $3$ compared with cognitively normal participants.

### Alternative Thresholding Approaches Reveal Similar Between-Group Differences in Global and Nodal Network Measures

We also assessed whether similar results could be obtained in the comparisons of PD and control groups using an alternative thresholding method. In this method, we retained the weights of the individual edges after binarizing the individual participant directed networks across the density range 1$\hbox{--}$50%. Regarding global network topology, the weighted analysis confirmed our earlier results by identifying significant increases in the global and local efficiency, clustering and transitivity in the participants with PD across a wide range of densities and at different temporal lags (Supplementary Figs 22 and 23). Regarding the nodal measures, we found that all regions identified with our earlier analysis showed similar significant increases or decreases at the same temporal lags. However, only a subset of them remained significant after controlling for multiple comparisons (FDR, *q* = 0.05), as shown in Supplementary Figs 24–29. The other three analysis methods did not identify any significant differences between groups.

### Alternative Methods of Directed Functional Connectivity Do Not Show Differences between PD Participants and Controls

We also calculated the whole-brain directed functional networks using the Granger causality method (“Methods: Granger causality”) and assessed the between-group differences in global and regional topology. Granger causality is an alternative approach that has been used to estimate the casual relation between the brain regions and directed information flow in the network based on temporal lags (Ghasemi and Mahloojifar 2013; Seth 2005; Wu et al. 2012). These analyses did not show any significant between-group differences in the global measures for the different model parameters (Supplementary Figs 30 and 31). Similarly, no significant nodal differences between groups were found at any density.

## Discussion

In this study, we propose a new method to analyze directed functional connectivity that uses the information stored in the temporal lags between the activation of brain regions. To our knowledge, there are currently no methods that allow assessing directed functional connectivity across the entire brain and studying the corresponding topological changes at multiple temporal lags. Our antisymmetric correlation method was developed to address this gap, showing that whole-brain directed connectivity is useful to characterize the connectomes of participants with PD by detecting widespread functional alterations that were not identifiable by conventional zero-lag methods or alternative methods of directed connectivity. In addition, we found that the changes identified by the antisymmetric correlation method remained significant with different tresholding methods and were associated with motor, executive, and memory deficits in PD participants, suggesting that they are clinically meaningful. Altogether, our findings indicate that the directional flow in brain activation signals contains exclusive information that is not captured by other methods, and could potentially be used as a new marker of functional network changes in PD.

Functional connectivity describes the statistical dependencies in the activation patterns between brain regions and is closely associated with behavior and cognitive functions (van den Heuvel and Pol 2010). Such statistical dependencies can be quantified using measures derived from graph theory, which typically consider two regions to be connected if the Pearson correlation between their activation signals is strong. However, this method is hindered by the fact that it only captures linear, simultaneous, and undirected dependencies between brain regions. There is evidence showing that brain activity is organized within multiple temporal functional modes (Mitra et al. 2014, 2015a). Therefore, the relationship between brain regions is not always linear and there are often delays between their activation signals (Ghosh et al. 2008; Jafri et al. 2008; Mijalkov et al. 2020), which results in directed activation patterns, with some regions being sources of activation, whereas others are destinations of activations (Mitra et al. 2014, 2015a). This lag organization is highly reproducible (Mitra et al. 2015a; Raut et al. 2020) and it can be altered in some disorders such as autism (Mitra et al. 2015b; Raatikainen et al. 2020), epilepsy (Bandt et al. 2019), schizophrenia (Jafri et al. 2008; White et al. 2010), or narcolepsy (Järvelä et al. 2020). Thus, capturing the information stored in these temporal delays or lags is crucial to obtain a more accurate characterization of the brain’s functional connectivity. While dynamic variations in these lagged patterns have been examined using ultrafast magnetic resonance encephalography scans, the lag structure in functional connectivity derived from functional MRI scans has been mainly assessed by interpolating a single temporal lag of maximal correlation over the course of the complete scan. In contrast to this approach, our antisymmetric correlation method evaluates the whole-brain functional connectivity at multiple temporal lags. This can provide new insights on the communication pathways between different brain areas and allows assessing the pathways derived from different temporal lags, which can change in the presence of neurodegenerative pathologies.

To demonstrate that the antisymmetric correlation method is useful to characterize functional connectivity, we tested its performance on a cohort of participants with PD and healthy controls. Our method detected an abnormal global topology in the functional connectomes of the PD group, characterized by increases in global efficiency, local efficiency, clustering, and transitivity, as well as decreases in the modularity when compared with healthy controls. The increases in global efficiency can be interpreted in light of previous studies showing that brain networks with a random organization have shorter network paths and greater global efficiency (Stam 2014). In addition to PD (Fang et al. 2017; Lopes et al. 2017; Tuovinen et al. 2018), this phenomenon has been shown to also occur in the networks of participants with schizophrenia (Fornito et al. 2012; van den Heuvel and Fornito 2014; Ganella et al. 2017) and Alzheimer’s disease (Tijms et al. 2013), being associated with executive impairment and other cognitive deficits (Stam 2014). On the other hand, the increases of clustering and transitivity in the networks of PD participants indicate an increase in the number of directed cyclic connections within local neighborhoods. This formation of closed triangles between neighboring regions increases the segregation and fragmentation of the functional networks, which has also been reported in some studies in participants with PD (Göttlich et al. 2013; Baggio et al. 2014). These changes were accompanied by lower modularity, suggesting that the fragmentation occurring in the networks of PD participants did not result in well-defined communities, which is normally regarded as a sign of brain pathology (Meunier et al. 2010). Thus, our findings show that the changes occurring in participants with PD reflect both increased integration and segregation in the directed functional networks. These changes were associated with worse performance on various clinical and cognitive tests measuring motor function, executive abilities, memory, attention, and visuospatial functions, suggesting that changes in global directed activation patterns can be an indicator of worse clinical progression in PD.

In addition to global network changes, we also observed alterations in the topology of specific brain regions. For instance, the precuneus showed an increase in the in-global efficiency and a decrease in the out-degree, which were associated with olfactory deficits. These findings are in line with previous evidence showing that the precuneus is a brain hub that plays an important role in memory, attention, and other cognitive functions (Cavanna and Trimble 2006). Several studies have shown changes in the functional connectivity patterns of the precuneus in PD participants (Delaveau et al. 2010; van Eimeren et al. 2009; Göttlich et al. 2013; Fang et al. 2017). Our findings offer an additional insight into the nature of these alterations. In particular, they indicate a specific shift to an increased number of in-coming connections accompanied by a decrease in the number of outgoing connections. This imbalance between in- and out-connectivity could possibly alter the role of the precuneus in the PD participants’ networks, making it an inefficient hub. Furthermore, this abnormal local topology could result in changes in the connectivity patterns within DMN and its strong connections with the olfactory system (Karunanayaka et al. 2017), leading to deficits in memory and loss of smell commonly experienced by PD participants.

Moreover, an increase of the in-global efficiency was observed in the fusiform gyrus. Similar changes have been previously observed in the connectivity of the fusiform gyrus in PD, which could lead to deficits in visual processing functions and decreased performance in verbal fluency tasks (Birn et al. 2010; Cardoso et al. 2010). We also observed an increase in the in-global efficiency in the parahippocampal gyrus and increase in the global efficiency of the lingual gyrus. Both regions have previously been shown to exhibit altered functional connectivity in PD participants (Sheng et al. 2014; Wen et al. 2016; Zhang et al. 2019).

Furthermore, the cerebellum showed an increase of the out-global efficiency in PD participants, which was significantly associated with motor scores. The cerebellum can affect motor and cognitive functions through its connections to cortical areas and the basal ganglia (Middleton and Strick 2000). Many studies have found an increase in the functional connectivity of the cerebellum in PD participants as a potentially compensatory mechanism, (Wu and Hallett 2005, 2013; Yang et al. 2013; Wen et al. 2016), in agreement with our results.

Finally, in our study, the superior frontal gyrus also showed an increased outflow connectivity in PD participants, while the thalamus displayed a decreased overall connectivity, which correlated with visuospatial deficits. Such changes in the functional activity of the frontal cortex have been associated with deficits in executive functions in participants with PD, for example, working memory, cognitive flexibility, and problem solving. Due to its strong connections with the striatum, these deficits have also been linked with dysfunction in the frontostriatal networks (Owen 2004; Parker et al. 2013). Being a part of the basal ganglia thalamo-cortical network, the thalamus carries information from the basal ganglia to the cerebral cortex, making it an important hub in functional brain networks (Hwang et al. 2017). As such, the thalamus plays an important role in many functions, such as motor abilities, visually guided actions, learning, and memory (Saalmann and Kastner 2011; Wolff and Vann 2019). Thus, our results provide further support to the role of the thalamus in contributing to functional abnormalities in the networks of PD participants and the various motor and nonmotor deficits they present.

There is ample evidence showing the existence of temporal lags in the activation signals between connected brain regions, which reflect the topological connectivity distance between them (Ghosh et al. 2008; Deco et al. 2009). An advantage of the antisymmetric correlation method is its ability to calculate functional connectomes at different temporal lags and, therefore, analyze functional connectivity at multiple connectivity scales. This allows investigating the topology of the network from regions connected by direct connections (small lags) to regions connected by indirect connections (large lags). Although we found a uniform global topology across all lags, the changes in regional topology varied substantially between different connectivity scales. These results suggest that, in participants with PD, the general efficiency in information transfer is maintained at multiple scales by conserving the global topological properties of the functional network. However, as different sets of brain regions co-activate at different temporal lags, the local topology of the regions varies with the value of the lags. As a result, abnormal regional changes are shown in distinct regions at different temporal lags in PD participants compared with controls, suggesting a high lag dependence on the nodal connectivity patterns in PD participants. The connectivity values of these different regions were associated with worse performance in motor and cognitive tests, suggesting that motor and cognitive deficits in participants with PD may be associated with brain connectivity changes occurring at different connectivity scales.

In order to assess which temporal scales were most relevant for our analysis, we plotted the connectivity weight profiles of the average connectivity matrices for both control and PD groups. For large delays, the connection weight histograms of both groups were narrow. This shows that large temporal lags are unable to capture variations in the directed activation flow in the network, instead assigning similar weights to a large number of connections. Therefore, in order to be able to capture this functional variation, we restricted our analysis to small temporal lags in the range $1\hbox{--} 7$. These conclusions were supported by the values calculated for the network diameter and in agreement with previous studies that showed that an optimum autoregressive model to analyze directed functional connectivity in PD is the one that includes seven time points (Ghasemi and Mahloojifar 2013).

While our findings demonstrate widespread functional connectivity changes in PD that can only be identified by the directed antisymmetric correlation networks, several studies have shown functional changes in undirected networks in PD participants from the PPMI database. While this apparent disagreement could stem from the inherent functional heterogeneity in participants with PD (Badea et al. 2017), such differences could be due to the use of different subsamples (Baggio et al. 2015b) which have different degrees of cognitive impairment (Lebedev et al. 2014; Chen et al. 2020) and medication (Sreenivasan et al. 2019) or evaluation of different network measures as well as different thresholding procedures (Prajapati and Emerson 2021).

Although the current study has several strengths, some limitations should also be recognized, which present opportunities for future work. First of all, our sample size was small, particularly the control group, which only included 15 individuals. Unfortunately, the PPMI cohort did not have a larger number of control participants with functional MRI data that we could use for the data analyses at the time we downloaded the functional MRI images. Another limitation is the fact that we did not apply multiple comparison corrections across the different temporal lags. This is due to the fact that our study was exploratory and our sample was relatively small so correcting the results across all lags would have led to a very stringent *P*-value (the corrections across 200 regions and 7 lags would require correcting for 1400 tests). Therefore, although we tested the antisymmetric correlations on a well-characterized sample of participants with PD, our results should be interpreted with caution, and they should be replicated in larger and independent cohorts that would allow applying more stringent corrections. Furthermore, several participants in the current cohort underwent functional MRI while on medication, which has previously been shown to influence brain connectivity (Palmer et al. 2009; Wu et al. 2009; Delaveau et al. 2010). In this study, we assessed the effects of medication on our results by performing correlation analyses between the levodopa-equivalent doses and the topological graph measures, as well as comparing the networks of medicated and nonmedicated PD groups. Our analyses showed that there was no association between medication doses and topological measures, and there were no differences in the global measures between the medicated and nonmedicated participants. The only significant results that were observed in medicated compared with nonedicated groups were an increase of the out-global efficiency in the superior occipital gyrus, superior parietal lobule, and precuneus at lag of $1$, a decrease in the in-degree of precuneus at lag $2$, and increases in the in-global efficiency and the in-degree of thalamus at lags $2$ and $3$. Since these regions did not overlap with the measures or regions that showed differences between PD and controls group in our main analysis, most likely they did not influence our results. In addition, it has also been demonstrated that PD participants with mild cognitive impairment have a different functional connectivity pattern when compared with cognitively normal participants (Baggio et al. 2014; Amboni et al. 2015; Baggio et al. 2015a; Lopes et al. 2017). As $20\%$ of the PD participants in our study were diagnosed with MCI, we also performed an additional analysis to compare them with the cognitively normal participants with PD. We found no topological differences in the directed functional connectomes between the two groups, suggesting that the presence of MCI also did not affect the main results. This result is in contrast with previous studies showing that the presence of MCI has an effect on network topology in participants with PD (Baggio et al. 2014; Lopes et al. 2017). This discrepancy is probably associated with the differences in clinical characteristics between our sample and the cohorts used in previous studies.

Despite these limitations, in this study, we show that the information stored in the temporal activation lags can be used to assess the directed connections between all the brain regions of the functional connectome. Our findings show that these directed connections can detect specific topological changes in participants with PD at multiple connectivity scales, offering increased sensitivity to PD-related changes compared with undirected methods. These findings suggest that our method could potentially be used to improve the diagnosis of PD or identify participants with worse disease progression.

## Supplementary Material

Mijalkovetal_SupplementaryMaterial_bhab237

## Notes

Data used in the preparation of this article were obtained from the Parkinson’s Progression Markers Initiative (PPMI) database (www.ppmi-info.org/data). For up-to-date information on the study, visit www.ppmi-info.org. PPMI—a public-private partnership—is funded by the Michael J. Fox Foundation for Parkinson’s Research and funding partners, including Abbvie, Allergan, Amathus, Avid, Biogen, Biolegend, Bristol-Myers-Squibb, Celgene, Denali, GE Healthcare, Genentech, Glaxo-Smith-Kline, Golub Capital, Handl, Insitro, Janssen, Lilly, Lundbeck, Merck, Meso Scale Discovery, Neurocrine, Pfizer, Piramal, Prevail, Roche, Sanofi Genzyme, Servier, Takeda, Teva, UCB, Verily, and Voyager.
