# Supplementary material for: Directed Brain Connectivity Identifies Widespread Functional Network Abnormalities in Parkinson’s Disease
Source: Cereb Cortex. 2021 Jul 30;32(3):593–607. doi: 10.1093/cercor/bhab237 (PMC8805861; doi:10.1093/cercor/bhab237)
Supplement: Mijalkovetal_SupplementaryMaterial_bhab237 [file mijalkovetal_supplementarymaterial_bhab237.zip › Mijalkovetal_SupplementaryMaterial_bhab237.pdf]

Directed brain connectivity identifies widespread functional network changes in Parkinson's disease

Supplementary information

Mite Mijalkov,[1,*] Giovanni Volpe,[2] and Joana B. Pereira[1,3,*]

[1] *Department of Neurobiology, Care Sciences and Society, Karolinska Institutet, Stockholm, Sweden*

[2] *Department of Physics, Goteborg University, Goteborg, Sweden*

[3] *Memory Research Unit, Department of Clinical Sciences Malmö, Lund University, Lund, Sweden*

[*] Corresponding authors: Email: mite.mijalkov@ki.se // joana.pereira@ki.se. Address: KI, Dept. NVS, division of clinical geriatrics, Neo 7th floor, Blickagången 16, 141 83 Huddinge, Sweden.

I. CORRELATION BETWEEN MATRICES OBTAINED WITH ZERO-LAG AND SYMMETRIC CORRELATION

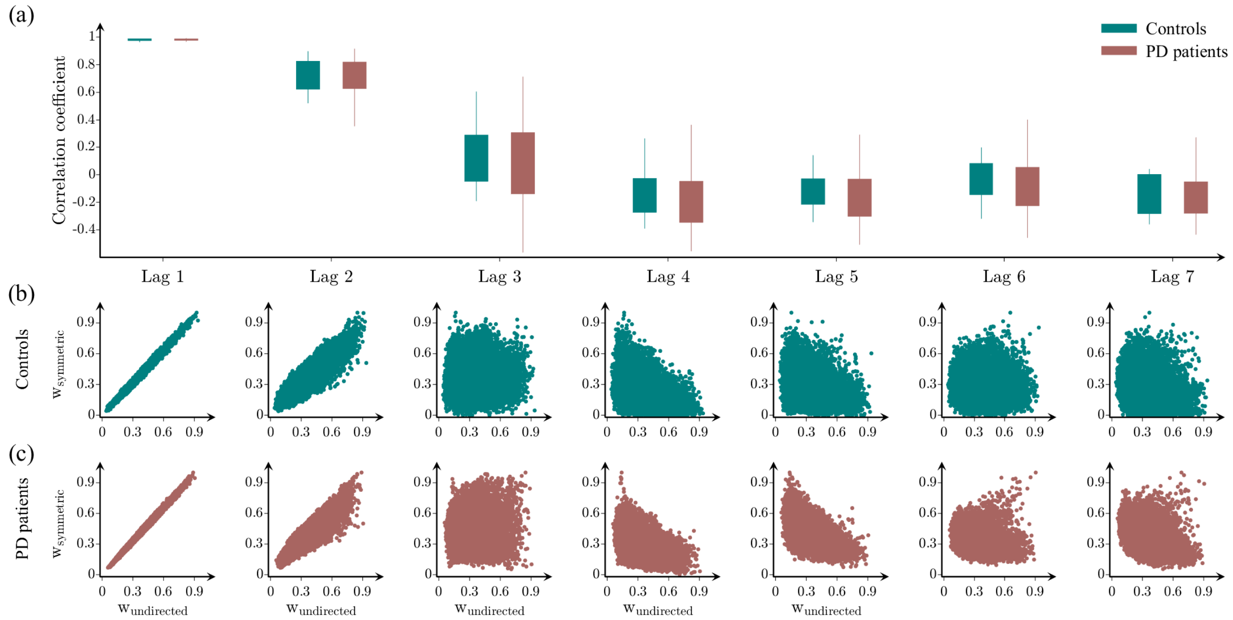

FIG. S1. **Correlation between zero-lag and symmetric correlation.** (a) Correlation coefficients between zero-lag and symmetric correlations as a function of the temporal lag at which symmetric correlations were calculated. We show the values obtained for controls (green boxes) and for PD patients (brown boxes); results are averages for all subjects within the particular group. The bottom and the top edges of the boxplots denote the 25th and 75th percentiles of the data, respectively. The whiskers extend to the largest and smallest data points that are not considered outliers. The strength of the normalized functional connections for symmetric vs zero-lag correlation for lags 1-7 (left to right) for the average connectivity matrices of (b) controls and (c) PD patients.

II. NETWORK DIAMETER PROVIDES AN ESTIMATION OF THE MAXIMUM TEMPORAL LAG

Different lags allow assessing functional activation between brain regions at different scales of topological connection distance. For example, lag 1 represents the network of regions connected via direct connections (i.e. connected by 1 edge), lag 2 represents the regions connected via indirect connections of length 2 (i.e. connected via 2 edges), etc. Therefore, we obtain an estimate of the temporal lags that can capture these different scales of topological connectivity by calculating the network diameter, i.e. the number of edges on the shortest path between the two nodes that are topologically most distant. We calculated the diameter for each participant-specific binary undirected matrices at various network densities (Supplementary Figure S2). We observe high network diameter at low densities, since the small number of edges in the network results in most nodes being connected through long paths. As the network density increases, nodes become connected through additional paths and shortcuts that are introduced in the network; hence the network diameter decreases. In the moderate density range, most participants from both groups have diameter values in the range 3-7, which suggests that the different scales of topological connectivity can be appropriately modelled by small temporal lags in that range, in agreement with Figure 2 in the main text.

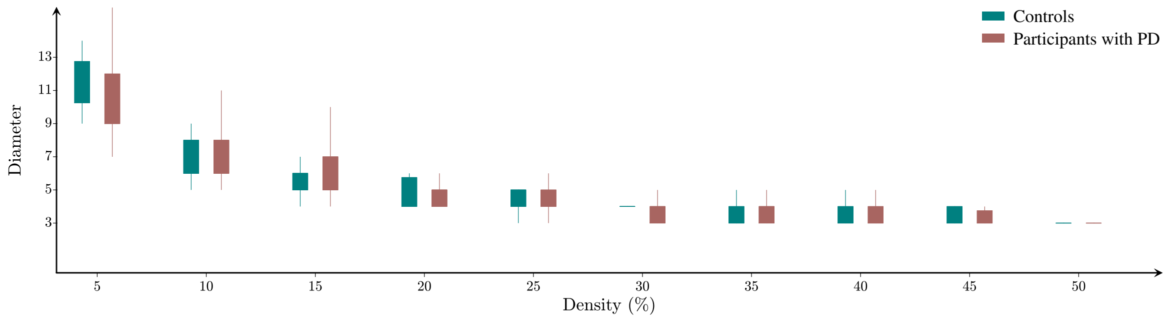

FIG. S2. **Network diameter.** Network diameter for controls (green boxes) and participants with PD (brown boxes) as a function of network density. The diameter for evaluated on the undirected binary zero-lag correlation matrices. The bottom and the top edges of the boxplots denote the 25th and 75th percentiles of the data, respectively. The whiskers extend to the largest and smallest data points that are not considered outliers.

III. DIFFERENCES IN GLOBAL TOPOLOGY

A. Lag 2

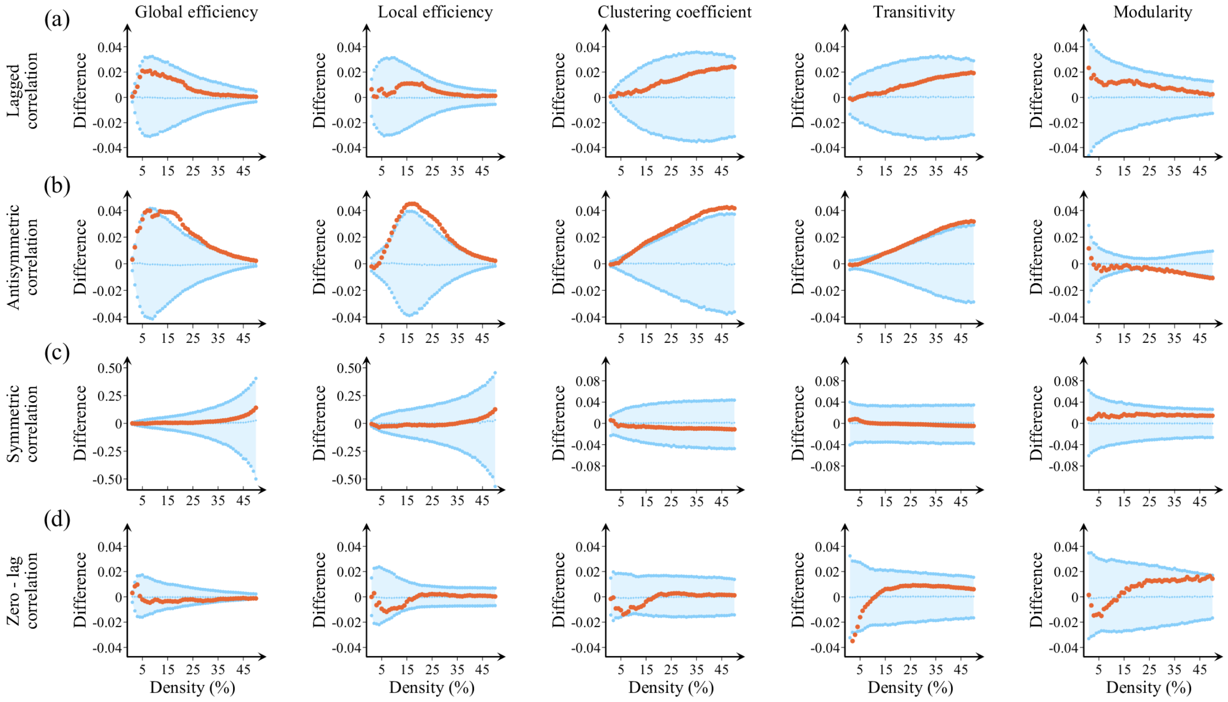

FIG. S3. **Differences between controls and PD patients in global network measures.** Plots showing the differences between controls and PD patients in the global efficiency, local efficiency, clustering coefficient, transitivity and modularity in the case of (a) lagged correlation, (b) anti-symmetric correlation, (c) symmetric correlation and (d) zero-lag correlation. The plots show the upper and lower bounds of the 95% confidence intervals (CI) in blue, and the differences in the network measures between groups in orange circles as a function of network density. The differences are considered statistically significant if they fall outside the CIs.

B. Lag 3

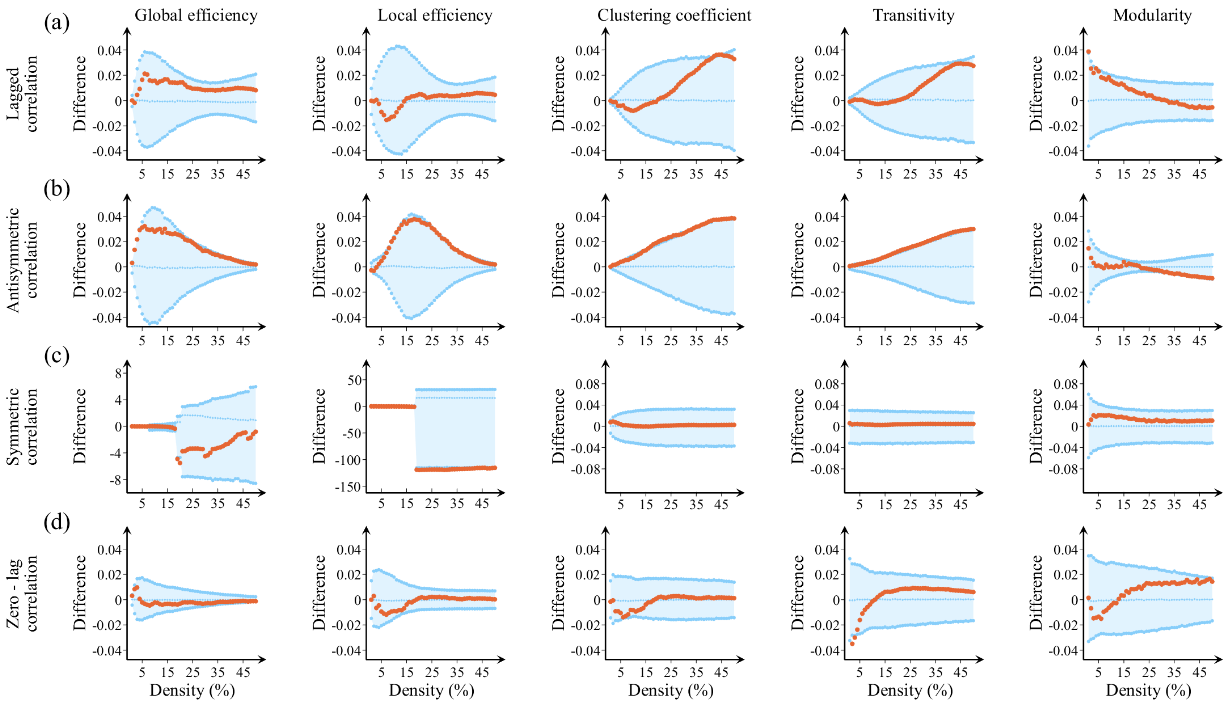

FIG. S4. **Differences between controls and PD patients in global network measures.** Plots showing the differences between controls and PD patients in the global efficiency, local efficiency, clustering coefficient, transitivity and modularity in the case of (a) lagged correlation, (b) anti-symmetric correlation, (c) symmetric correlation and (d) zero-lag correlation. The plots show the upper and lower bounds of the 95% confidence intervals (CI) in blue, and the differences in the network measures between groups in orange circles as a function of network density. The differences are considered statistically significant if they fall outside the CIs.

C. Lag 4

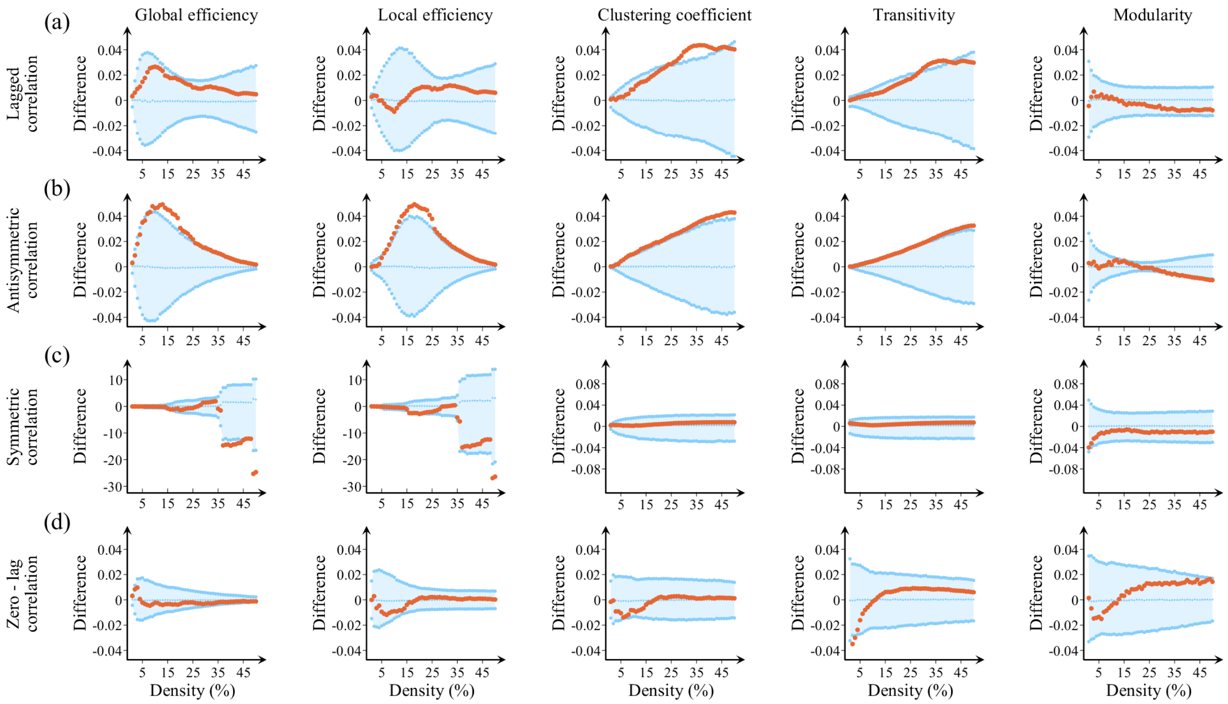

FIG. S5. **Differences between controls and PD patients in global network measures.** Plots showing the differences between controls and PD patients in the global efficiency, local efficiency, clustering coefficient, transitivity and modularity in the case of (a) lagged correlation, (b) anti-symmetric correlation, (c) symmetric correlation and (d) zero-lag correlation. The plots show the upper and lower bounds of the 95% confidence intervals (CI) in blue, and the differences in the network measures between groups in orange circles as a function of network density. The differences are considered statistically significant if they fall outside the CIs.

D. Lag 5

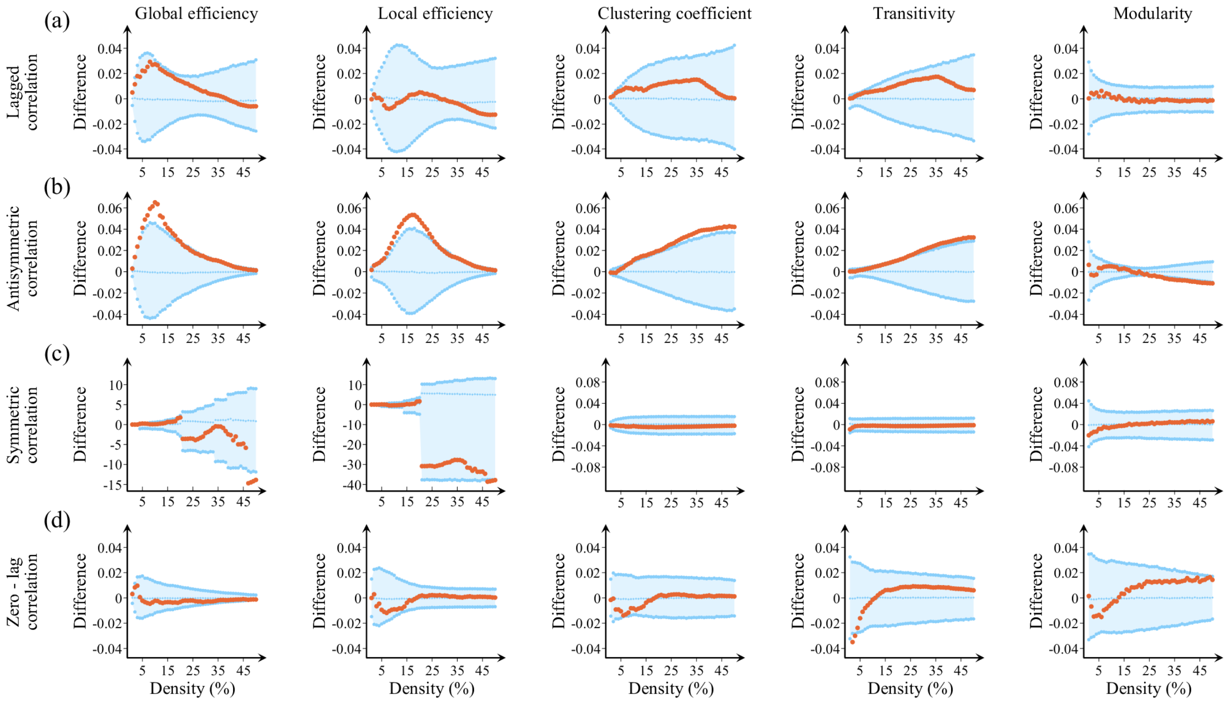

FIG. S6. **Differences between controls and PD patients in global network measures.** Plots showing the differences between controls and PD patients in the global efficiency, local efficiency, clustering coefficient, transitivity and modularity in the case of (a) lagged correlation, (b) anti-symmetric correlation, (c) symmetric correlation and (d) zero-lag correlation. The plots show the upper and lower bounds of the 95% confidence intervals (CI) in blue, and the differences in the network measures between groups in orange circles as a function of network density. The differences are considered statistically significant if they fall outside the CIs.

E. Lag 6

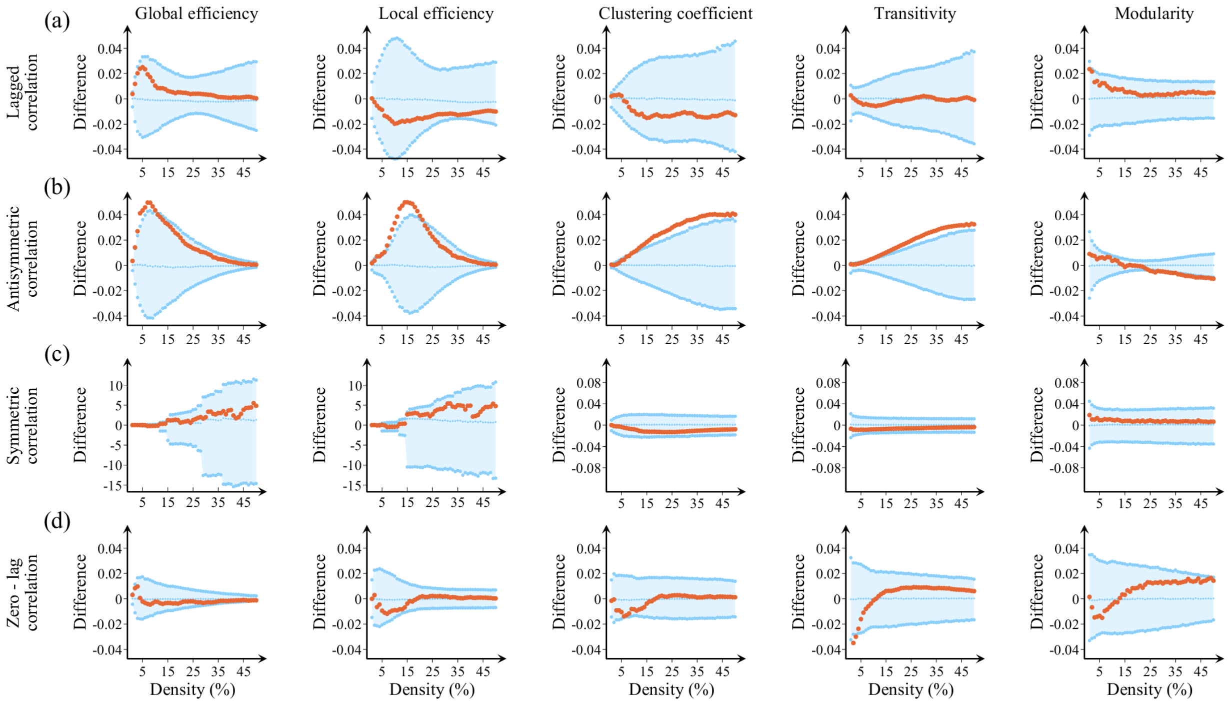

FIG. S7. **Differences between controls and PD patients in global network measures.** Plots showing the differences between controls and PD patients in the global efficiency, local efficiency, clustering coefficient, transitivity and modularity in the case of (a) lagged correlation, (b) anti-symmetric correlation, (c) symmetric correlation and (d) zero-lag correlation. The plots show the upper and lower bounds of the 95% confidence intervals (CI) in blue, and the differences in the network measures between groups in orange circles as a function of network density. The differences are considered statistically significant if they fall outside the CIs.

F. Lag 7

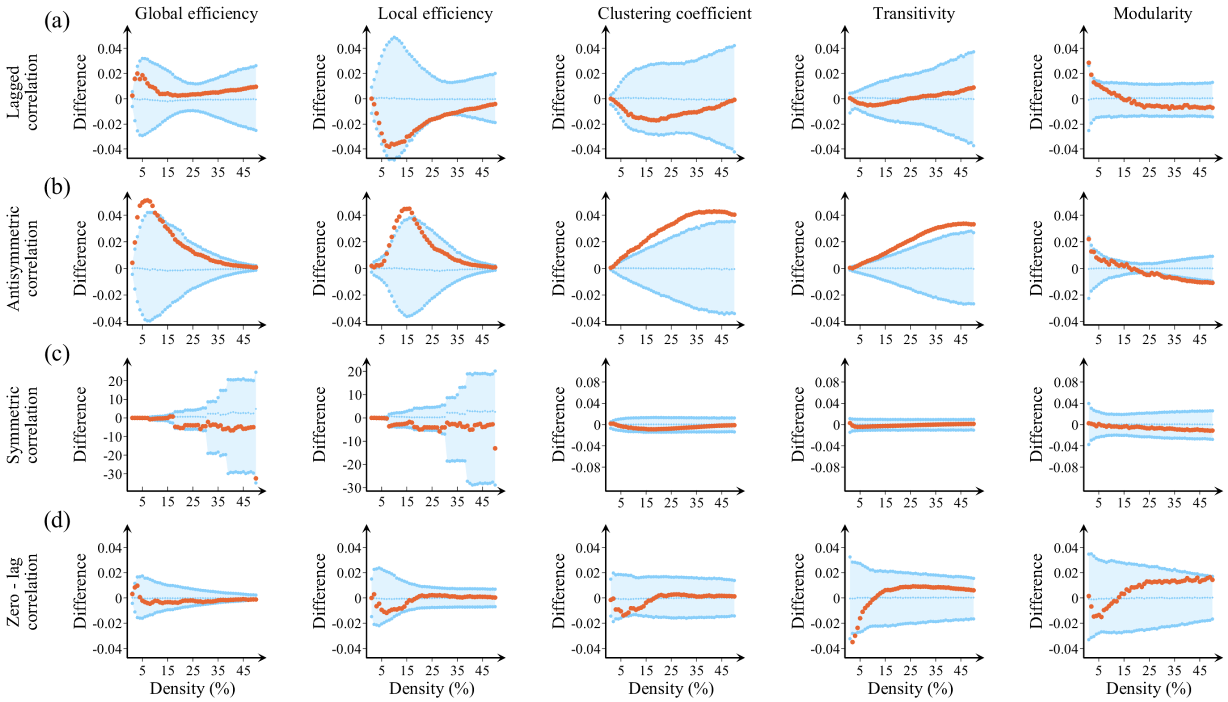

FIG. S8. **Differences between controls and PD patients in global network measures.** Plots showing the differences between controls and PD patients in the global efficiency, local efficiency, clustering coefficient, transitivity and modularity in the case of (a) lagged correlation, (b) anti-symmetric correlation, (c) symmetric correlation and (d) zero-lag correlation. The plots show the upper and lower bounds of the 95% confidence intervals (CI) in blue, and the differences in the network measures between groups in orange circles as a function of network density. The differences are considered statistically significant if they fall outside the CIs.

IV. GLOBAL MEASURES AS A FUNCTION OF DENSITY: ANTI-SYMMETRIC CORRELATION

A. Temporal lags 1 to 4

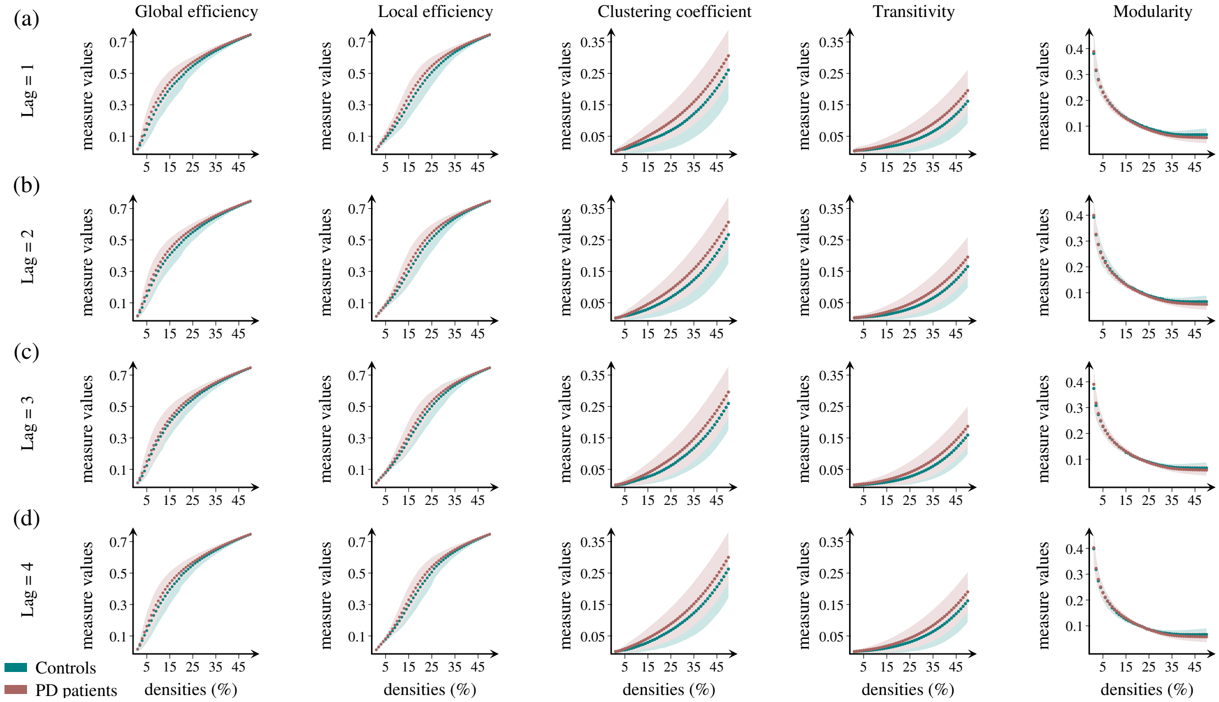

FIG. S9. **Global measures as a function of density.** Plots showing the measure values for controls and PD patients in the global efficiency, local efficiency, clustering coefficient, transitivity and modularity for temporal lags of (a) 1, (b) 2, (c) 3 and (d) 4 for the anti-symmetric correlation method. The shaded areas indicate the standard deviation of the corresponding group.

B. Temporal lags 5 to 7

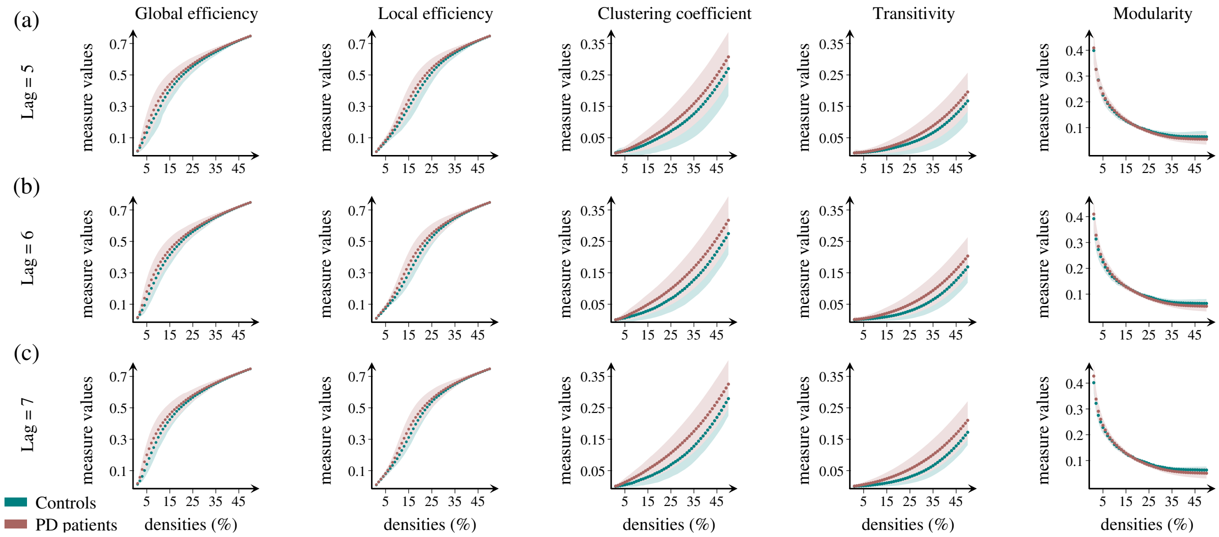

FIG. S10. **Global measures as a function of density.** Plots showing the measure values for controls and PD patients in the global efficiency, local efficiency, clustering coefficient, transitivity and modularity for temporal lags of (a) 5, (b) 6 and (c) 7 for the anti-symmetric correlation method. The shaded areas indicate the standard deviation of the corresponding group.

V. DIFFERENCES IN NODAL TOPOLOGY: AREA UNDER THE CURVE (AUC) ANALYSIS

A. Lag 1

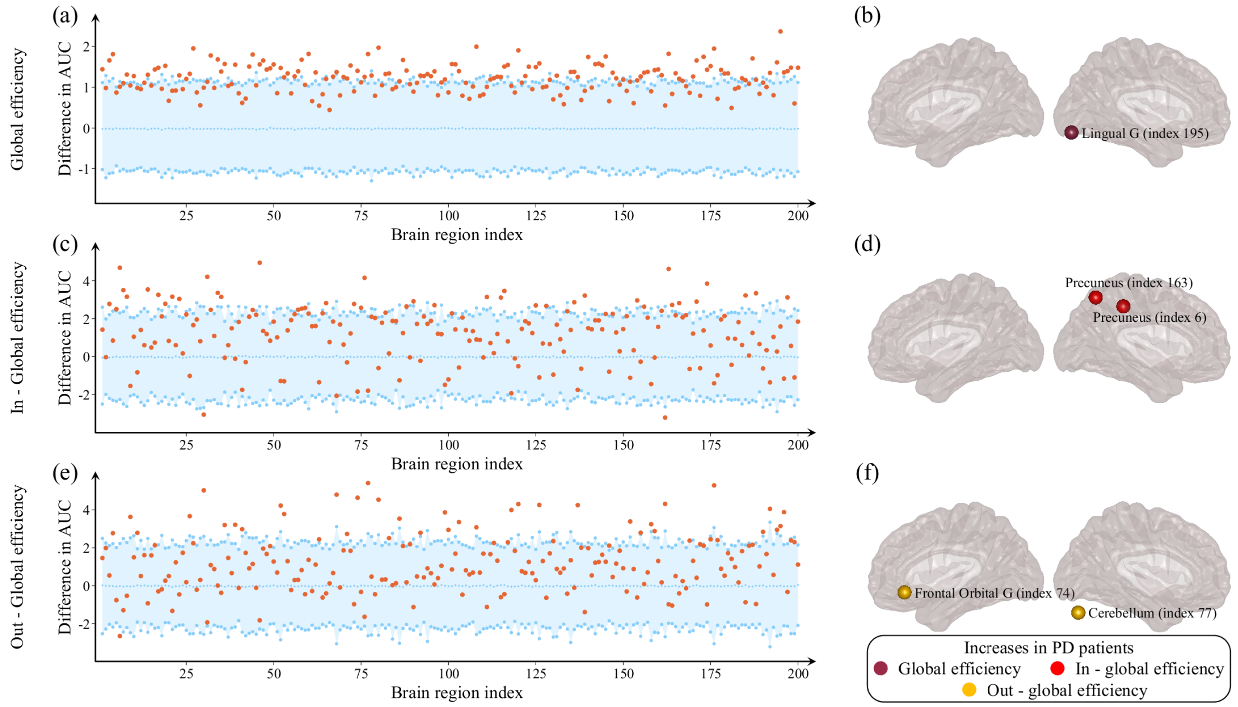

FIG. S11. **Differences between controls and PD patients in nodal network measures using AUC analysis for lag 1.** Plots showing differences in the areas under the curve between controls and PD patients in the (a) global efficiency, (b) in - global efficiency and (c) out - global efficiency for all brain regions in the density range 1% - 50%. The plots show the upper and lower bounds of the 95% confidence intervals (CI) in blue, and the differences in the network measures between groups in orange circles as a function of network density. The differences are considered statistically significant if they fall outside the CIs. (b, d, f) Regions that showed significant between-group differences in the corresponding measures after correction for multiple comparisons (FDR, $q < 0.05$).

B. Lag 2

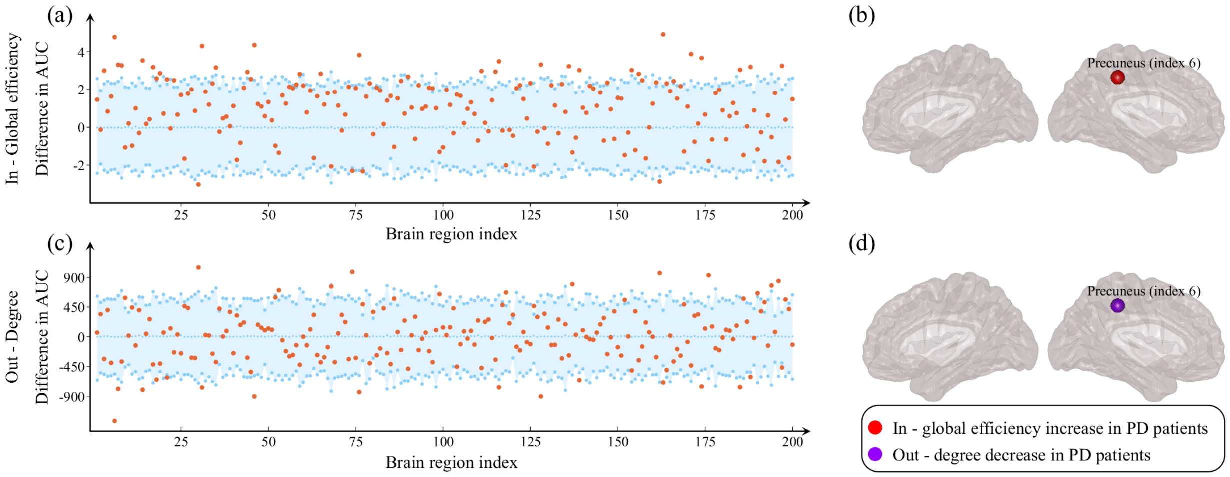

FIG. S12. **Differences between controls and PD patients in nodal network measures using AUC analysis for lag 2.** Plots showing differences in the areas under the curve between controls and PD patients in the (a) in - global efficiency and (c) out - degree for all brain regions in the density range 1% - 50%. The plots show the upper and lower bounds of the 95% confidence intervals (CI) in blue, and the differences in the network measures between groups in orange circles as a function of network density. The differences are considered statistically significant if they fall outside the CIs. (b, d) Regions that showed significant between-group differences in the corresponding measures after correction for multiple comparisons (FDR, $q < 0.05$).

C. Lag 3

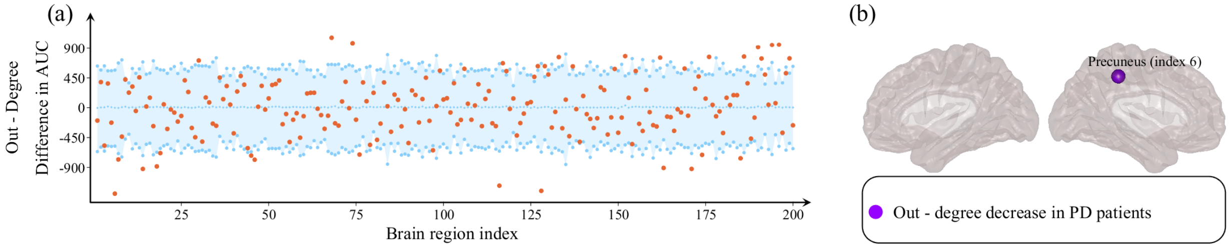

FIG. S13. **Differences between controls and PD patients in nodal network measures using AUC analysis for lag 3.** Plots showing differences in the areas under the curve between controls and PD patients in the (a) out - degree for all brain regions in the density range 1% - 50%. The plots show the upper and lower bounds of the 95% confidence intervals (CI) in blue, and the differences in the network measures between groups in orange circles as a function of network density. The differences are considered statistically significant if they fall outside the CIs. (b) Regions that showed significant between-group differences in the corresponding measures after correction for multiple comparisons (FDR, $q < 0.05$).

D. Lag 4

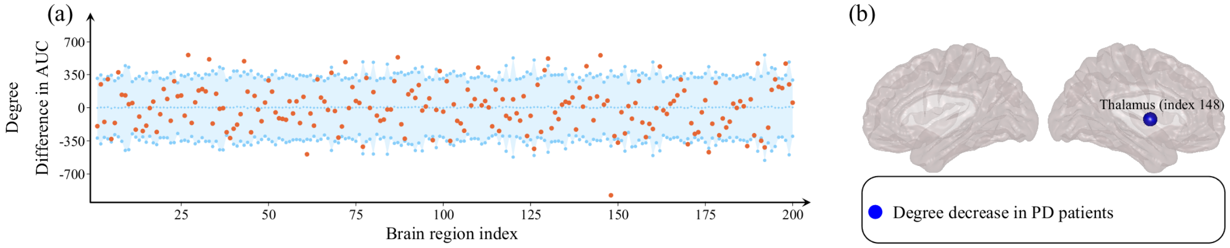

FIG. S14. **Differences between controls and PD patients in nodal network measures using AUC analysis for lag 4.** Plots showing differences in the areas under the curve between controls and PD patients in the (a) degree for all brain regions in the density range 1% - 50%. The plots show the upper and lower bounds of the 95% confidence intervals (CI) in blue, and the differences in the network measures between groups in orange circles as a function of network density. The differences are considered statistically significant if they fall outside the CIs. (b) Regions that showed significant between-group differences in the corresponding measures after correction for multiple comparisons (FDR, $q < 0.05$).

E. Lag 5

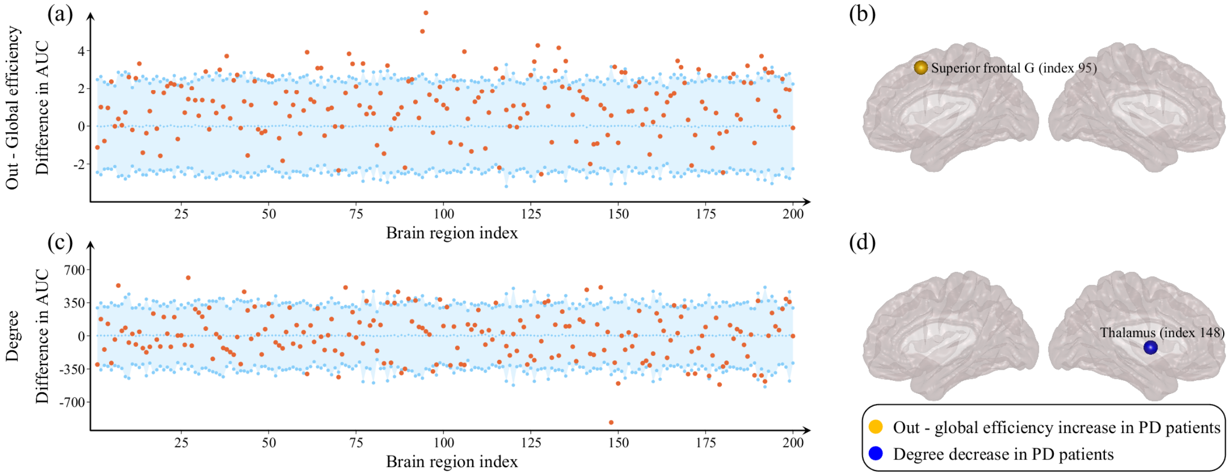

FIG. S15. **Differences between controls and PD patients in nodal network measures using AUC analysis for lag 5.** Plots showing differences in the areas under the curve between controls and PD patients in the (a) out - global efficiency and (c) degree for all brain regions in the density range 1% - 50%. The plots show the upper and lower bounds of the 95% confidence intervals (CI) in blue, and the differences in the network measures between groups in orange circles as a function of network density. The differences are considered statistically significant if they fall outside the CIs. (b, d) Regions that showed significant between-group differences in the corresponding measures after correction for multiple comparisons (FDR, $q < 0.05$).

F. Lag 7

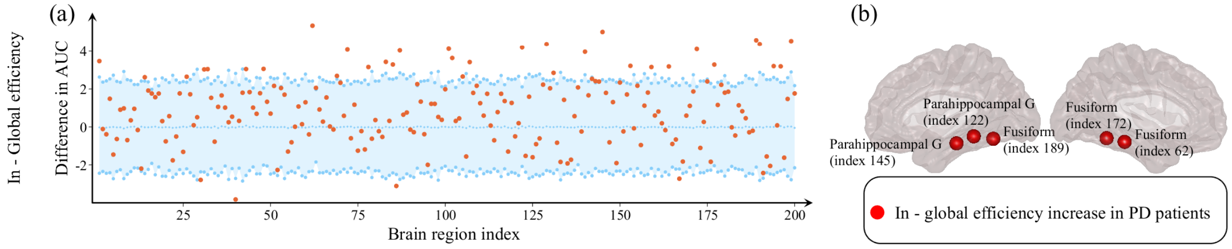

FIG. S16. **Differences between controls and PD patients in nodal network measures using AUC analysis for lag 7.** Plots showing differences in the areas under the curve between controls and PD patients in the (a) in - global efficiency for all brain regions in the density range 1% - 50%. The plots show the upper and lower bounds of the 95% confidence intervals (CI) in blue, and the differences in the network measures between groups in orange circles as a function of network density. The differences are considered statistically significant if they fall outside the CIs. (b) Regions that showed significant between-group differences in the corresponding measures after correction for multiple comparisons (FDR, $q < 0.05$).

G. List of brain regions

TABLE S1: Name and the corresponding index for all brain regions. The 200 brain regions were derived from the Craddock atlas.

Index	Name
Brain region index: 1	Inferior Occipital Gyrus
Brain region index: 2	Angular Gyrus
Brain region index: 3	Precuneus
Brain region index: 4	Insula
Brain region index: 5	Anterior cingulate
Brain region index: 6	Precuneus
Brain region index: 7	Angular Gyrus
Brain region index: 8	Postcentral Gyrus
Brain region index: 9	Cerebellum
Brain region index: 10	Cerebellum
Brain region index: 11	Middle temporal Gyrus
Brain region index: 12	Frontal pole
Brain region index: 13	Medial Frontal Gyrus
Brain region index: 14	Angular Gyrus
Brain region index: 15	Putamen
Brain region index: 16	Inferior Occipital Gyrus
Brain region index: 17	Precentral
Brain region index: 18	Thalamus
Brain region index: 19	Precuneus
Brain region index: 20	Insula
Brain region index: 21	Postcentral Gyrus
Brain region index: 22	Anterior cingulate
Brain region index: 23	Middle frontal Gyrus
Brain region index: 24	Superior temporal Gyrus

Continued on next page

TABLE S1 – continued from previous page

Brain region index: 25	Middle frontal Gyrus
Brain region index: 26	Inferior Occipital Gyrus
Brain region index: 27	Parahippocampal Gyrus
Brain region index: 28	Postcentral Gyrus
Brain region index: 29	Cingulate Gyrus
Brain region index: 30	Brainstem
Brain region index: 31	Superior Parietal Lobule
Brain region index: 32	Temporal pole
Brain region index: 33	Supramarginal Gyrus
Brain region index: 34	Precentral
Brain region index: 35	Insula
Brain region index: 36	Cerebellum
Brain region index: 37	Thalamus
Brain region index: 38	Middle frontal Gyrus
Brain region index: 39	Middle temporal Gyrus
Brain region index: 40	Anterior cingulate
Brain region index: 41	Cerebellum
Brain region index: 42	Frontal pole
Brain region index: 43	Temporal pole
Brain region index: 44	Occipital pole
Brain region index: 45	Thalamus
Brain region index: 46	Posterior Cingulate
Brain region index: 47	Caudate
Brain region index: 48	Parahippocampal Gyrus
Brain region index: 49	Middle temporal Gyrus
Brain region index: 50	Superior frontal Gyrus
Brain region index: 51	Anterior cingulate
Brain region index: 52	Brainstem

Continued on next page

TABLE S1 – continued from previous page

Brain region index: 53	Frontal Orbital Gyrus
Brain region index: 54	Cuneus
Brain region index: 55	Anterior cingulate
Brain region index: 56	Angular Gyrus
Brain region index: 57	Frontal Orbital Gyrus
Brain region index: 58	Precuneus
Brain region index: 59	Insula
Brain region index: 60	Postcentral Gyrus
Brain region index: 61	Middle frontal Gyrus
Brain region index: 62	Fusiform
Brain region index: 63	Fusiform
Brain region index: 64	Middle frontal Gyrus
Brain region index: 65	Superior Parietal Lobule
Brain region index: 66	Superior temporal Gyrus
Brain region index: 67	Putamen
Brain region index: 68	Midbrain
Brain region index: 69	Middle temporal Gyrus
Brain region index: 70	Lingual Gyrus
Brain region index: 71	Frontal Orbital Gyrus
Brain region index: 72	Middle temporal Gyrus
Brain region index: 73	Precentral
Brain region index: 74	Frontal Orbital Gyrus
Brain region index: 75	Frontal pole
Brain region index: 76	Posterior Cingulate
Brain region index: 77	Cerebellum
Brain region index: 78	Temporal pole
Brain region index: 79	Anterior cingulate
Brain region index: 80	Cerebellum

Continued on next page

TABLE S1 – continued from previous page

Brain region index: 81	Cuneus
Brain region index: 82	Angular Gyrus
Brain region index: 83	Superior temporal Gyrus
Brain region index: 84	Caudate
Brain region index: 85	Inferior Occipital Gyrus
Brain region index: 86	Brainstem
Brain region index: 87	Fusiform
Brain region index: 88	Postcentral Gyrus
Brain region index: 89	Lingual Gyrus
Brain region index: 90	Precentral
Brain region index: 91	Medial Frontal Gyrus
Brain region index: 92	Amygdala
Brain region index: 93	Supramarginal Gyrus
Brain region index: 94	Caudate
Brain region index: 95	Superior frontal Gyrus
Brain region index: 96	Postcentral Gyrus
Brain region index: 97	Superior Occipital Gyrus
Brain region index: 98	Precentral
Brain region index: 99	Inferior Temporal Gyrus
Brain region index: 100	Inferior Temporal Gyrus
Brain region index: 101	Inferior Temporal Gyrus
Brain region index: 102	Inferior Occipital Gyrus
Brain region index: 103	Cerebellum
Brain region index: 104	Frontal pole
Brain region index: 105	Lingual Gyrus
Brain region index: 106	Middle frontal Gyrus
Brain region index: 107	Middle temporal Gyrus
Brain region index: 108	Lingual Gyrus

Continued on next page

TABLE S1 – continued from previous page

Brain region index: 109	Frontal pole
Brain region index: 110	Temporal pole
Brain region index: 111	Precentral
Brain region index: 112	Frontal Orbital Gyrus
Brain region index: 113	Middle frontal Gyrus
Brain region index: 114	Superior Occipital Gyrus
Brain region index: 115	Precentral
Brain region index: 116	Postcentral Gyrus
Brain region index: 117	Middle temporal Gyrus
Brain region index: 118	Cerebellum
Brain region index: 119	Inferior Frontal Gyrus
Brain region index: 120	Cerebellum
Brain region index: 121	Insula
Brain region index: 122	Parahippocampal Gyrus
Brain region index: 123	Paracentral lobule
Brain region index: 124	Frontal pole
Brain region index: 125	Middle frontal Gyrus
Brain region index: 126	Cerebellum
Brain region index: 127	Middle frontal Gyrus
Brain region index: 128	Inferior Parietal Gyrus
Brain region index: 129	Superior temporal Gyrus
Brain region index: 130	Midbrain
Brain region index: 131	Inferior Occipital Gyrus
Brain region index: 132	Superior Parietal Gyrus
Brain region index: 133	Superior frontal Gyrus
Brain region index: 134	Precentral
Brain region index: 135	Caudate
Brain region index: 136	Superior Occipital Gyrus

Continued on next page

TABLE S1 – continued from previous page

Brain region index: 137	Insula
Brain region index: 138	Fusiform
Brain region index: 139	Frontal pole
Brain region index: 140	Middle temporal Gyrus
Brain region index: 141	Inferior Frontal Gyrus
Brain region index: 142	Middle Occipital Gyrus
Brain region index: 143	Inferior Occipital Gyrus
Brain region index: 144	Inferior Frontal Gyrus
Brain region index: 145	Parahippocampal Gyrus
Brain region index: 146	Superior temporal Gyrus
Brain region index: 147	Precuneus
Brain region index: 148	Thalamus
Brain region index: 149	Medial Frontal Gyrus
Brain region index: 150	Inferior Occipital Gyrus
Brain region index: 151	Middle frontal Gyrus
Brain region index: 152	Cerebellum
Brain region index: 153	Superior temporal Gyrus
Brain region index: 154	Postcentral Gyrus
Brain region index: 155	Parahippocampal Gyrus
Brain region index: 156	Superior Parietal Lobule
Brain region index: 157	Postcentral Gyrus
Brain region index: 158	Occipital pole
Brain region index: 159	Fusiform
Brain region index: 160	Anterior cingulate
Brain region index: 161	Superior frontal Gyrus
Brain region index: 162	Cerebellum
Brain region index: 163	Precuneus
Brain region index: 164	Inferior Frontal Gyrus

Continued on next page

TABLE S1 – continued from previous page

Brain region index: 165	Precentral
Brain region index: 166	Angular Gyrus
Brain region index: 167	Inferior Frontal Gyrus
Brain region index: 168	Middle frontal Gyrus
Brain region index: 169	Inferior Frontal Gyrus
Brain region index: 170	Superior Occipital Gyrus
Brain region index: 171	Superior Parietal Lobule
Brain region index: 172	Fusiform
Brain region index: 173	Superior frontal Gyrus
Brain region index: 174	Precuneus
Brain region index: 175	Inferior Occipital cortex
Brain region index: 176	Cerebellum
Brain region index: 177	Lingual Gyrus
Brain region index: 178	Putamen
Brain region index: 179	Lingual Gyrus
Brain region index: 180	Postcentral Gyrus
Brain region index: 181	Temporal pole
Brain region index: 182	Supplementary motor cortex
Brain region index: 183	Frontal pole
Brain region index: 184	Insula
Brain region index: 185	Transverse Temporal Gyrus
Brain region index: 186	Superior frontal Gyrus
Brain region index: 187	Superior frontal Gyrus
Brain region index: 188	Superior Parietal Lobule
Brain region index: 189	Fusiform
Brain region index: 190	Brainstem
Brain region index: 191	Superior frontal Gyrus
Brain region index: 192	Cerebellum

Continued on next page

TABLE S1 – continued from previous page

Brain region index: 193	Superior frontal Gyrus
Brain region index: 194	Midbrain
Brain region index: 195	Lingual Gyrus
Brain region index: 196	Insula
Brain region index: 197	Precuneus
Brain region index: 198	Fusiform
Brain region index: 199	Brainstem
Brain region index: 200	Transverse Temporal Gyrus

VI. CORRELATION ANALYSIS WITH CLINICAL MEASURES IN PD PATIENTS

A. Significant correlations between UPDRS-III motor scores and global measures: Lags 1-4

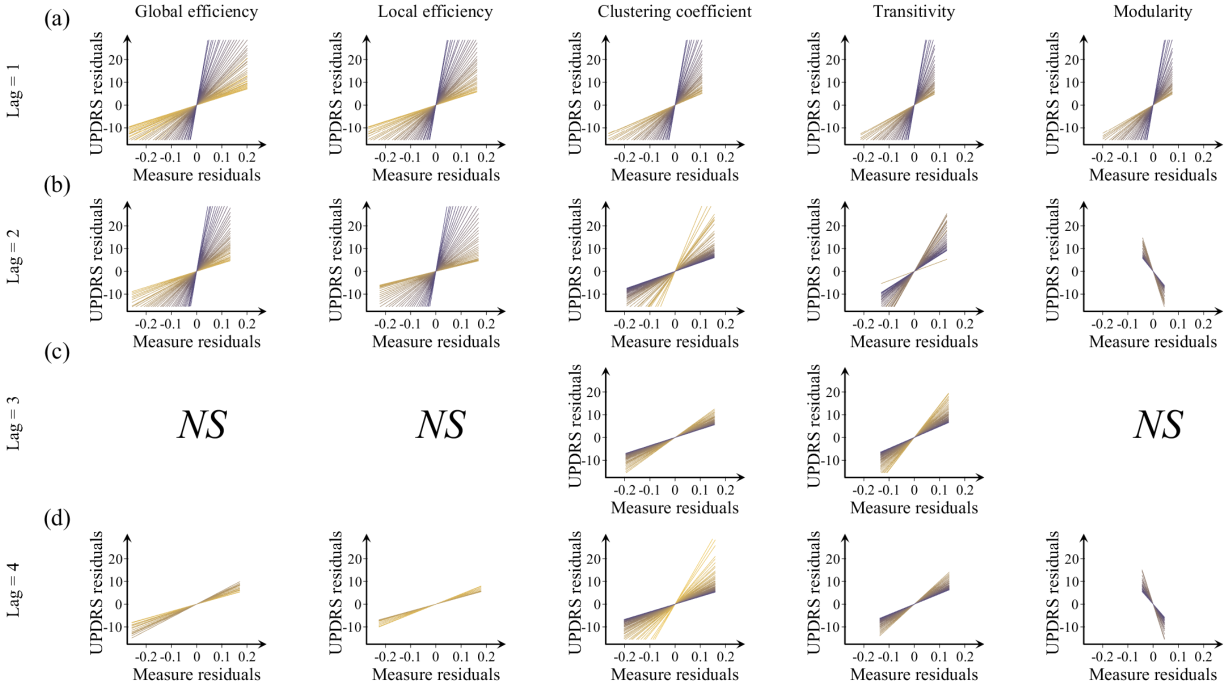

FIG. S17. **Correlation between UPDRS-III motor scores and global measures for PD group: Lags 1-4.** Plots showing the best line fit between UPDRS-III scores and global measures for PD group for lag of (a) 1, (b) 2, (c) 3 and (d) 4. The original values were regressed with age, sex and motion parameters as covariates; the residuals of the regression were used to calculate the best line fit. Only the fits that were statistically significant ($p < 0.05$) are shown. The different lines represent fits at different densities; the results for small densities are shown in brighter yellow. NS: no significant fits between the UPDRS-III motor scores and global measure were found at the specified lag.

B. Significant correlations between UPDRS-III motor scores and global measures: Lags 5-7

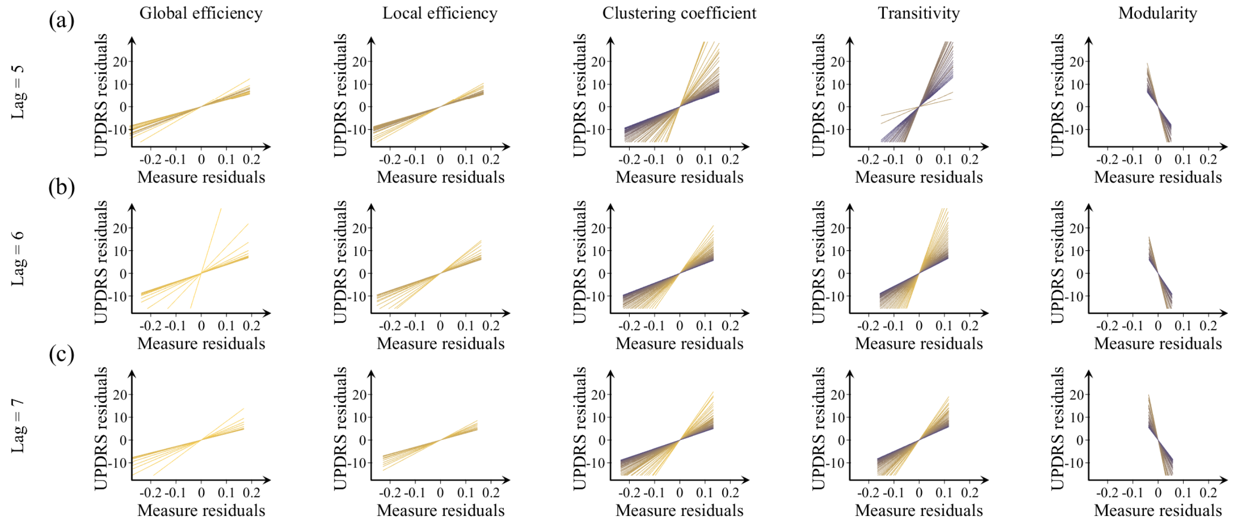

FIG. S18. **Correlation between UPDRS-III motor scores and global measures for PD group: Lags 5-7.** Plots showing the best line fit between UPDRS-III scores and global measures for PD group for lag of (a) 5, (b) 6 and (c) 7. The original values were regressed with age, sex and motion parameters as covariates; the residuals of the regression were used to calculate the best line fit. Only the fits that were statistically significant ($p < 0.05$) are shown. The different lines represent fits at different densities; the results for small densities are shown in brighter yellow.

C. Significant correlations between Letter Number Sequencing test scores and global measures: Lags 1-4

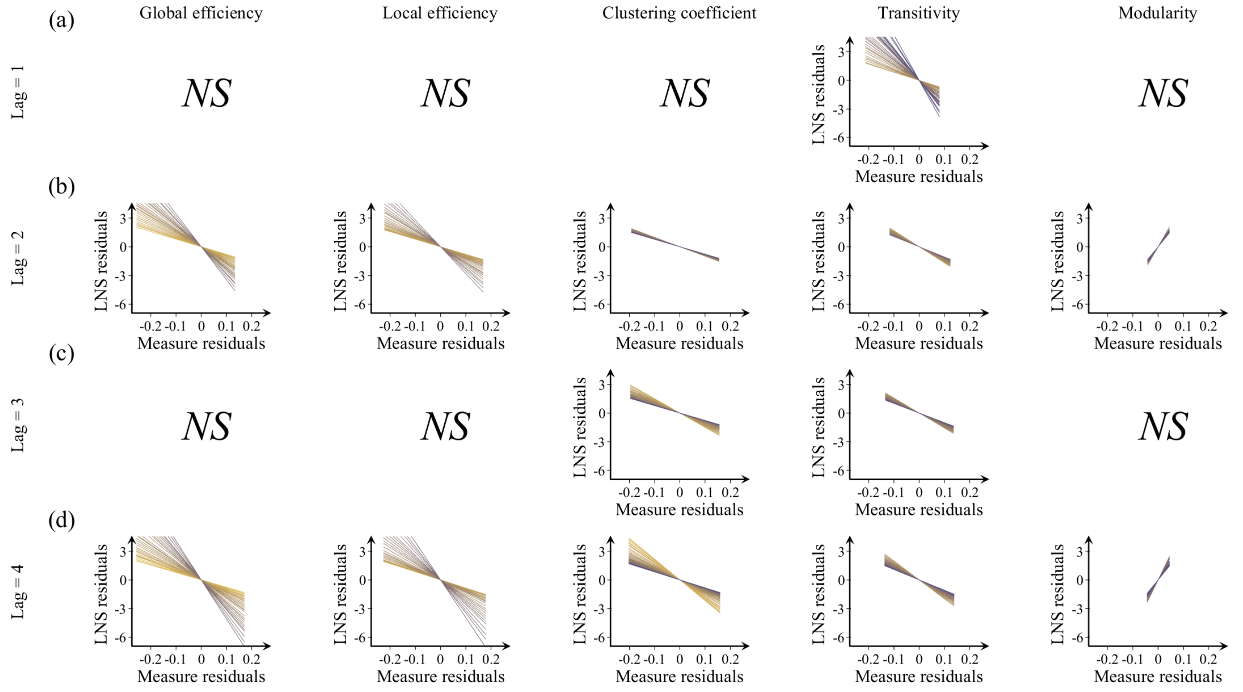

FIG. S19. **Correlation between Letter Number Sequencing test scores and global measures for PD group: Lags 1-4.** Plots showing the best line fit between LNS test scores and global measures for PD group for lag of (a) 1, (b) 2, (c) 3 and (d) 4. The original values were regressed with age, sex and motion parameters as covariates; the residuals of the regression were used to calculate the best line fit. Only the fits that were statistically significant ($p < 0.05$) are shown. The different lines represent fits at different densities; the results for small densities are shown in brighter yellow. NS: no significant fits between the test scores and global measure were found at the specified lag.

D. Significant correlations between Letter Number Sequencing test scores and global measures: Lags 5-7

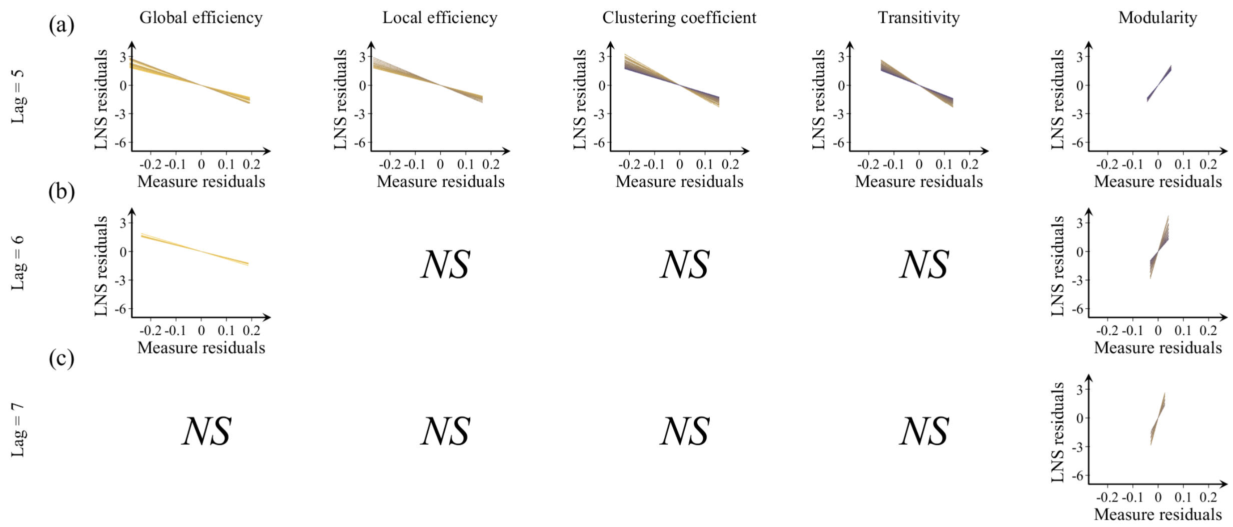

FIG. S20. **Correlation between Letter Number Sequencing test scores and global measures for PD group: Lags 5-7.** Plots showing the best line fit between LNS test scores and global measures for PD group for lag of (a) 5, (b) 6 and (c) 7. The original values were regressed with age, sex and motion parameters as covariates; the residuals of the regression were used to calculate the best line fit. Only the fits that were statistically significant ($p < 0.05$) are shown. The different lines represent fits at different densities; the results for small densities are shown in brighter yellow. NS: no significant fits between the test scores and global measure were found at the specified lag.

E. Significant correlations between multiple test scores and global measures

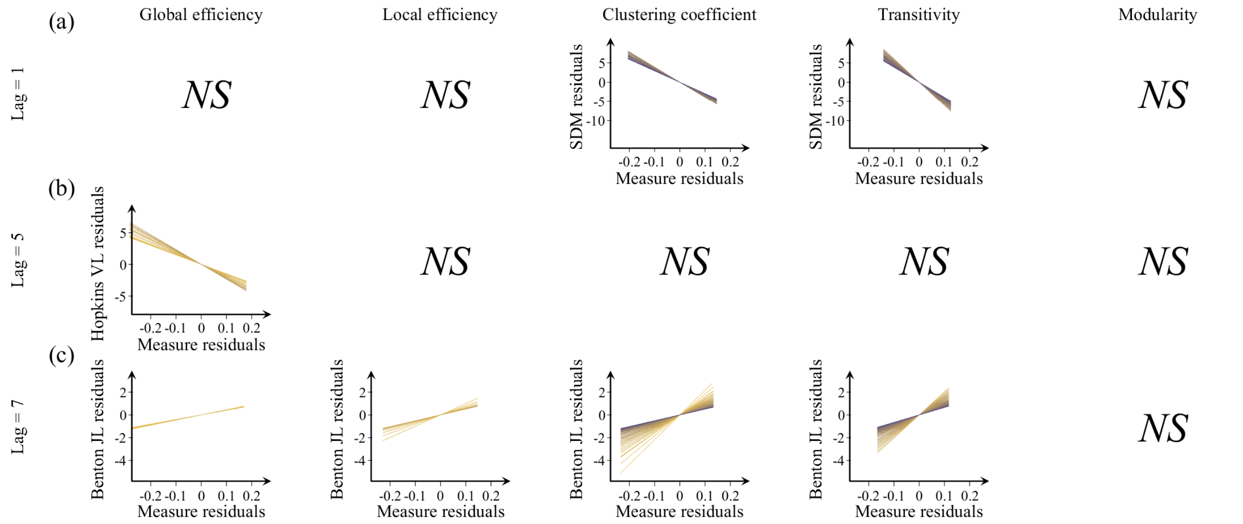

FIG. S21. **Correlation between multiple test scores and global measures for PD group.** Plots showing the best line fit between test scores and global measures for PD group for: (a) Symbol Digit Modalities test scores and measures at lag of 1, (b) Hopkins verbal learning test and measures at lag of 5 and (c) Benton's judgement of line orientation test scores and measures at lag of 7. The original values were regressed with age, sex and motion parameters as covariates; the residuals of the regression were used to calculate the best line fit. Only the fits that were statistically significant ($p < 0.05$) are shown. The different lines represent fits at different densities; the results for small densities are shown in brighter yellow. NS: no significant fits between the test scores and global measure were found at the specified lag.

TABLE S2: Correlation analysis between various global measures calculated at lag of 1 and the UPDRS-III motor scores.

Global efficiency					
**density**	**coefficient**	**p-value**	**density**	**coefficient**	**p-value**
2.000	0.330	0.001	27.000	0.279	0.007
3.000	0.325	0.002	28.000	0.268	0.010
4.000	0.341	0.001	29.000	0.269	0.010
5.000	0.343	0.001	30.000	0.270	0.010
6.000	0.319	0.002	31.000	0.263	0.012
7.000	0.310	0.003	32.000	0.262	0.012
8.000	0.297	0.004	33.000	0.257	0.014
9.000	0.286	0.006	34.000	0.255	0.015
10.000	0.288	0.006	35.000	0.256	0.014
11.000	0.283	0.007	36.000	0.259	0.013
12.000	0.283	0.007	37.000	0.261	0.013
13.000	0.285	0.006	38.000	0.257	0.014
14.000	0.298	0.004	39.000	0.254	0.015
15.000	0.301	0.004	40.000	0.257	0.014
16.000	0.297	0.004	41.000	0.253	0.015
17.000	0.291	0.005	42.000	0.252	0.016
18.000	0.290	0.005	43.000	0.248	0.018
19.000	0.290	0.005	44.000	0.252	0.016
20.000	0.286	0.006	45.000	0.238	0.023
21.000	0.282	0.007	46.000	0.249	0.017
22.000	0.284	0.006	47.000	0.256	0.014
23.000	0.275	0.008	48.000	0.255	0.015
24.000	0.273	0.009	49.000	0.262	0.012
25.000	0.278	0.008	50.000	0.257	0.014

Continued on next page

TABLE S2 – continued from previous page

26.000	0.282	0.007			

Local efficiency

density	coefficient	p-value	density	coefficient	p-value
10.000	0.215	0.041	31.000	0.268	0.010
11.000	0.234	0.026	32.000	0.269	0.010
12.000	0.245	0.019	33.000	0.264	0.011
13.000	0.256	0.014	34.000	0.264	0.011
14.000	0.280	0.007	35.000	0.265	0.011
15.000	0.290	0.005	36.000	0.268	0.010
16.000	0.281	0.007	37.000	0.269	0.010
17.000	0.271	0.009	38.000	0.265	0.011
18.000	0.272	0.009	39.000	0.258	0.013
19.000	0.273	0.009	40.000	0.260	0.013
20.000	0.271	0.009	41.000	0.256	0.014
21.000	0.267	0.011	42.000	0.255	0.015
22.000	0.267	0.011	43.000	0.250	0.017
23.000	0.259	0.013	44.000	0.252	0.016
24.000	0.258	0.014	45.000	0.239	0.022
25.000	0.267	0.011	46.000	0.249	0.017
26.000	0.270	0.010	47.000	0.256	0.014
27.000	0.268	0.010	48.000	0.254	0.015
28.000	0.261	0.013	49.000	0.261	0.012
29.000	0.266	0.011	50.000	0.257	0.014
30.000	0.270	0.010			

Clustering coefficient

density	coefficient	p-value	density	coefficient	p-value
16.000	0.295	0.005	34.000	0.279	0.008

Continued on next page

TABLE S2 – continued from previous page

17.000	0.288	0.006	35.000	0.277	0.008
18.000	0.291	0.005	36.000	0.280	0.007
19.000	0.287	0.006	37.000	0.279	0.007
20.000	0.284	0.006	38.000	0.278	0.008
21.000	0.280	0.007	39.000	0.278	0.008
22.000	0.279	0.007	40.000	0.280	0.007
23.000	0.277	0.008	41.000	0.279	0.007
24.000	0.278	0.008	42.000	0.278	0.008
25.000	0.283	0.007	43.000	0.277	0.008
26.000	0.283	0.007	44.000	0.278	0.008
27.000	0.282	0.007	45.000	0.275	0.008
28.000	0.277	0.008	46.000	0.276	0.008
29.000	0.280	0.007	47.000	0.277	0.008
30.000	0.282	0.007	48.000	0.278	0.008
31.000	0.277	0.008	49.000	0.280	0.007
32.000	0.278	0.008	50.000	0.279	0.007
33.000	0.276	0.008			
**Transitivity**					
**density**	**coefficient**	**p-value**	**density**	**coefficient**	**p-value**
20.000	0.266	0.011	36.000	0.282	0.007
21.000	0.267	0.011	37.000	0.281	0.007
22.000	0.269	0.010	38.000	0.280	0.007
23.000	0.270	0.010	39.000	0.282	0.007
24.000	0.270	0.010	40.000	0.283	0.007
25.000	0.276	0.008	41.000	0.282	0.007
26.000	0.278	0.008	42.000	0.281	0.007
27.000	0.279	0.007	43.000	0.280	0.007

Continued on next page

TABLE S2 – continued from previous page

28.000	0.277	0.008	44.000	0.281	0.007
29.000	0.280	0.007	45.000	0.278	0.008
30.000	0.281	0.007	46.000	0.279	0.007
31.000	0.277	0.008	47.000	0.280	0.007
32.000	0.278	0.008	48.000	0.281	0.007
33.000	0.277	0.008	49.000	0.282	0.007
34.000	0.281	0.007	50.000	0.281	0.007
35.000	0.281	0.007			
**Modularity**					
**density**	**coefficient**	**p-value**	**density**	**coefficient**	**p-value**
21.000	-0.248	0.018	36.000	-0.231	0.028
22.000	-0.305	0.003	37.000	-0.245	0.019
23.000	-0.344	0.001	38.000	-0.239	0.022
24.000	-0.265	0.011	39.000	-0.261	0.013
25.000	-0.371	0.000	40.000	-0.265	0.011
26.000	-0.322	0.002	41.000	-0.267	0.010
27.000	-0.323	0.002	42.000	-0.271	0.009
28.000	-0.260	0.013	43.000	-0.275	0.008
29.000	-0.281	0.007	44.000	-0.273	0.009
30.000	-0.319	0.002	45.000	-0.272	0.009
31.000	-0.254	0.015	46.000	-0.272	0.009
32.000	-0.303	0.004	47.000	-0.273	0.009
33.000	-0.263	0.012	48.000	-0.277	0.008
34.000	-0.249	0.017	49.000	-0.279	0.007
35.000	-0.254	0.015	50.000	-0.277	0.008

TABLE S3: Correlation analysis between various global measures calculated at lag of 2 and the UPDRS-III motor scores.

Global efficiency					
**density**	**coefficient**	**p-value**	**density**	**coefficient**	**p-value**
12.000	0.262	0.012	32.000	0.263	0.012
13.000	0.263	0.012	33.000	0.264	0.011
14.000	0.264	0.011	34.000	0.257	0.014
15.000	0.271	0.009	35.000	0.260	0.013
16.000	0.268	0.010	36.000	0.263	0.012
17.000	0.266	0.011	37.000	0.258	0.014
18.000	0.270	0.010	38.000	0.257	0.014
19.000	0.283	0.007	39.000	0.254	0.015
20.000	0.271	0.009	40.000	0.251	0.016
21.000	0.271	0.009	41.000	0.250	0.017
22.000	0.277	0.008	42.000	0.252	0.016
23.000	0.280	0.007	43.000	0.248	0.018
24.000	0.278	0.008	44.000	0.249	0.017
25.000	0.276	0.008	45.000	0.247	0.018
26.000	0.275	0.008	46.000	0.243	0.020
27.000	0.270	0.010	47.000	0.245	0.019
28.000	0.265	0.011	48.000	0.241	0.021
29.000	0.264	0.011	49.000	0.237	0.024
30.000	0.272	0.009	50.000	0.246	0.019
31.000	0.267	0.011			
**Local efficiency**					
**density**	**coefficient**	**p-value**	**density**	**coefficient**	**p-value**
12.000	0.207	0.049	32.000	0.258	0.014
13.000	0.218	0.038	33.000	0.263	0.012

Continued on next page

TABLE S3 – continued from previous page

14.000	0.220	0.036	34.000	0.259	0.013
15.000	0.229	0.029	35.000	0.260	0.013
16.000	0.227	0.031	36.000	0.263	0.012
17.000	0.227	0.030	37.000	0.259	0.013
18.000	0.227	0.031	38.000	0.257	0.014
19.000	0.234	0.026	39.000	0.255	0.015
20.000	0.231	0.028	40.000	0.253	0.016
21.000	0.231	0.028	41.000	0.253	0.016
22.000	0.241	0.021	42.000	0.255	0.015
23.000	0.248	0.018	43.000	0.250	0.017
24.000	0.253	0.016	44.000	0.251	0.017
25.000	0.254	0.015	45.000	0.249	0.017
26.000	0.255	0.015	46.000	0.243	0.020
27.000	0.254	0.015	47.000	0.244	0.020
28.000	0.254	0.015	48.000	0.242	0.021
29.000	0.255	0.015	49.000	0.237	0.024
30.000	0.263	0.012	50.000	0.246	0.019
31.000	0.261	0.012			
**Clustering coefficient**					
**density**	**coefficient**	**p-value**	**density**	**coefficient**	**p-value**
11.000	0.261	0.012	31.000	0.281	0.007
12.000	0.269	0.010	32.000	0.279	0.007
13.000	0.279	0.007	33.000	0.282	0.007
14.000	0.275	0.008	34.000	0.281	0.007
15.000	0.265	0.011	35.000	0.282	0.007
16.000	0.256	0.014	36.000	0.282	0.007
17.000	0.259	0.013	37.000	0.279	0.007

Continued on next page

TABLE S3 – continued from previous page

18.000	0.264	0.012	38.000	0.280	0.007
19.000	0.261	0.012	39.000	0.281	0.007
20.000	0.263	0.012	40.000	0.281	0.007
21.000	0.264	0.011	41.000	0.283	0.007
22.000	0.265	0.011	42.000	0.285	0.006
23.000	0.271	0.009	43.000	0.284	0.006
24.000	0.276	0.008	44.000	0.284	0.006
25.000	0.277	0.008	45.000	0.284	0.006
26.000	0.282	0.007	46.000	0.282	0.007
27.000	0.281	0.007	47.000	0.282	0.007
28.000	0.279	0.007	48.000	0.283	0.007
29.000	0.278	0.008	49.000	0.283	0.007
30.000	0.282	0.007	50.000	0.283	0.006
**Transitivity**					
**density**	**coefficient**	**p-value**	**density**	**coefficient**	**p-value**
21.000	0.276	0.008	36.000	0.289	0.005
22.000	0.277	0.008	37.000	0.288	0.006
23.000	0.283	0.007	38.000	0.288	0.006
24.000	0.285	0.006	39.000	0.290	0.005
25.000	0.286	0.006	40.000	0.290	0.005
26.000	0.289	0.005	41.000	0.290	0.005
27.000	0.290	0.005	42.000	0.292	0.005
28.000	0.286	0.006	43.000	0.291	0.005
29.000	0.287	0.006	44.000	0.292	0.005
30.000	0.290	0.005	45.000	0.291	0.005
31.000	0.289	0.006	46.000	0.289	0.005
32.000	0.287	0.006	47.000	0.289	0.005

Continued on next page

TABLE S3 – continued from previous page

33.000	0.289	0.005	48.000	0.290	0.005
34.000	0.289	0.005	49.000	0.290	0.005
35.000	0.290	0.005	50.000	0.290	0.005
**Modularity**					
**density**	**coefficient**	**p-value**	**density**	**coefficient**	**p-value**
28.000	-0.271	0.009	40.000	-0.257	0.014
29.000	-0.277	0.008	41.000	-0.266	0.011
30.000	-0.332	0.001	42.000	-0.276	0.008
31.000	-0.314	0.002	43.000	-0.275	0.008
32.000	-0.295	0.005	44.000	-0.275	0.008
33.000	-0.279	0.007	45.000	-0.278	0.008
34.000	-0.284	0.006	46.000	-0.274	0.008
35.000	-0.267	0.010	47.000	-0.275	0.008
36.000	-0.267	0.011	48.000	-0.275	0.008
37.000	-0.270	0.010	49.000	-0.278	0.008
38.000	-0.255	0.015	50.000	-0.280	0.007
39.000	-0.251	0.016			

TABLE S4: Correlation analysis between various global measures calculated at lag of 3 and the UPDRS-III motor scores.

Clustering coefficient					
**density**	**coefficient**	**p-value**	**density**	**coefficient**	**p-value**
16.000	0.261	0.012	38.000	0.280	0.007
17.000	0.265	0.011	39.000	0.281	0.007
18.000	0.264	0.012	40.000	0.283	0.007
19.000	0.268	0.010	41.000	0.283	0.007
20.000	0.268	0.010	42.000	0.281	0.007
21.000	0.267	0.010	43.000	0.281	0.007
22.000	0.267	0.011	44.000	0.281	0.007
23.000	0.270	0.010	45.000	0.284	0.006
24.000	0.272	0.009	46.000	0.286	0.006
25.000	0.274	0.009	47.000	0.284	0.006
26.000	0.271	0.009	48.000	0.285	0.006
27.000	0.270	0.010	49.000	0.284	0.006
28.000	0.274	0.009	50.000	0.283	0.007
37.000	0.279	0.008			

Transitivity					
**density**	**coefficient**	**p-value**	**density**	**coefficient**	**p-value**
14.000	0.248	0.018	33.000	0.309	0.003
15.000	0.260	0.013	34.000	0.308	0.003
16.000	0.265	0.011	35.000	0.307	0.003
17.000	0.272	0.009	36.000	0.304	0.003
18.000	0.278	0.008	37.000	0.302	0.004
19.000	0.286	0.006	38.000	0.302	0.004
20.000	0.288	0.006	39.000	0.303	0.004
21.000	0.290	0.005	40.000	0.303	0.003

Continued on next page

TABLE S4 – continued from previous page

22.000	0.293	0.005	41.000	0.302	0.004
23.000	0.295	0.004	42.000	0.300	0.004
24.000	0.299	0.004	43.000	0.299	0.004
25.000	0.302	0.004	44.000	0.299	0.004
26.000	0.300	0.004	45.000	0.300	0.004
27.000	0.300	0.004	46.000	0.301	0.004
28.000	0.304	0.003	47.000	0.299	0.004
29.000	0.305	0.003	48.000	0.299	0.004
30.000	0.307	0.003	49.000	0.298	0.004
31.000	0.308	0.003	50.000	0.296	0.004
32.000	0.311	0.003			

TABLE S5: Correlation analysis between various global measures calculated at lag of 4 and the UPDRS-III motor scores.

**Global efficiency**					
**density**	**coefficient**	**p-value**	**density**	**coefficient**	**p-value**
9.000	0.292	0.005	17.000	0.243	0.020
10.000	0.286	0.006	18.000	0.238	0.023
11.000	0.273	0.009	19.000	0.241	0.022
12.000	0.286	0.006	20.000	0.241	0.021
13.000	0.275	0.008	21.000	0.231	0.028
14.000	0.263	0.012	22.000	0.226	0.031
15.000	0.250	0.017	23.000	0.218	0.038
16.000	0.244	0.020	25.000	0.220	0.036
**Local efficiency**					
**density**	**coefficient**	**p-value**	**density**	**coefficient**	**p-value**
10.000	0.241	0.021	16.000	0.263	0.012
11.000	0.268	0.010	17.000	0.257	0.014
12.000	0.279	0.007	18.000	0.251	0.016
13.000	0.284	0.006	19.000	0.243	0.020
14.000	0.281	0.007	20.000	0.239	0.023
15.000	0.269	0.010	21.000	0.223	0.034
**Clustering**					
**density**	**coefficient**	**p-value**	**density**	**coefficient**	**p-value**
6.000	0.247	0.018	29.000	0.274	0.009
7.000	0.270	0.010	30.000	0.273	0.009
8.000	0.277	0.008	31.000	0.273	0.009
9.000	0.252	0.016	32.000	0.271	0.009
10.000	0.261	0.012	33.000	0.275	0.008
11.000	0.260	0.013	34.000	0.274	0.009

Continued on next page

TABLE S5 – continued from previous page

12.000	0.259	0.013	35.000	0.270	0.010
13.000	0.269	0.010	36.000	0.274	0.009
14.000	0.264	0.012	37.000	0.272	0.009
15.000	0.271	0.009	38.000	0.273	0.009
16.000	0.275	0.008	39.000	0.271	0.009
17.000	0.272	0.009	40.000	0.270	0.010
18.000	0.273	0.009	41.000	0.270	0.010
19.000	0.276	0.008	42.000	0.271	0.009
20.000	0.277	0.008	43.000	0.266	0.011
21.000	0.273	0.009	44.000	0.266	0.011
22.000	0.275	0.008	45.000	0.265	0.011
23.000	0.275	0.008	46.000	0.263	0.012
24.000	0.274	0.009	47.000	0.264	0.011
25.000	0.280	0.007	48.000	0.259	0.013
26.000	0.279	0.007	49.000	0.264	0.012
27.000	0.278	0.008	50.000	0.260	0.013
28.000	0.273	0.009			
**Transitivity**					
**density**	**coefficient**	**p-value**	**density**	**coefficient**	**p-value**
23.000	0.302	0.004	37.000	0.293	0.005
24.000	0.303	0.004	38.000	0.294	0.005
25.000	0.305	0.003	39.000	0.292	0.005
26.000	0.306	0.003	40.000	0.291	0.005
27.000	0.306	0.003	41.000	0.290	0.005
28.000	0.302	0.004	42.000	0.290	0.005
29.000	0.302	0.004	43.000	0.286	0.006
30.000	0.300	0.004	44.000	0.285	0.006

Continued on next page

TABLE S5 – continued from previous page

31.000	0.301	0.004	45.000	0.283	0.007
32.000	0.298	0.004	46.000	0.280	0.007
33.000	0.300	0.004	47.000	0.280	0.007
34.000	0.297	0.004	48.000	0.275	0.008
35.000	0.293	0.005	49.000	0.278	0.008
36.000	0.295	0.005	50.000	0.275	0.008

Modularity

density	coefficient	p-value	density	coefficient	p-value
32.000	-0.335	0.001	42.000	-0.287	0.006
33.000	-0.349	0.001	43.000	-0.275	0.008
34.000	-0.323	0.002	44.000	-0.279	0.007
35.000	-0.309	0.003	45.000	-0.277	0.008
36.000	-0.297	0.004	46.000	-0.273	0.009
37.000	-0.294	0.005	47.000	-0.266	0.011
38.000	-0.276	0.008	48.000	-0.261	0.012
39.000	-0.289	0.005	49.000	-0.262	0.012
40.000	-0.291	0.005	50.000	-0.258	0.014
41.000	-0.290	0.005			

TABLE S6: Correlation analysis between various global measures calculated at lag of 5 and the UPDRS-III motor scores.

Global efficiency					
**density**	**coefficient**	**p-value**	**density**	**coefficient**	**p-value**
3.000	0.353	0.001	12.000	0.251	0.016
4.000	0.352	0.001	13.000	0.239	0.022
5.000	0.343	0.001	14.000	0.229	0.029
6.000	0.326	0.002	15.000	0.258	0.014
7.000	0.319	0.002	16.000	0.257	0.014
8.000	0.302	0.004	17.000	0.261	0.013
9.000	0.289	0.005	18.000	0.256	0.014
10.000	0.272	0.009	19.000	0.245	0.019
11.000	0.261	0.012	20.000	0.246	0.019
**Local efficiency**					
**density**	**coefficient**	**p-value**	**density**	**coefficient**	**p-value**
8.000	0.224	0.033	18.000	0.279	0.007
9.000	0.253	0.015	19.000	0.260	0.013
10.000	0.282	0.007	20.000	0.256	0.014
11.000	0.303	0.003	21.000	0.250	0.017
12.000	0.302	0.004	22.000	0.245	0.019
13.000	0.300	0.004	23.000	0.241	0.021
14.000	0.302	0.004	24.000	0.234	0.026
15.000	0.304	0.003	25.000	0.232	0.027
16.000	0.297	0.004	26.000	0.222	0.034
17.000	0.293	0.005	27.000	0.220	0.036
**Clustering**					
**density**	**coefficient**	**p-value**	**density**	**coefficient**	**p-value**
10.000	0.287	0.006	31.000	0.308	0.003

Continued on next page

TABLE S6 – continued from previous page

11.000	0.294	0.005	32.000	0.309	0.003
12.000	0.277	0.008	33.000	0.310	0.003
13.000	0.293	0.005	34.000	0.311	0.003
14.000	0.298	0.004	35.000	0.310	0.003
15.000	0.303	0.004	36.000	0.310	0.003
16.000	0.298	0.004	37.000	0.307	0.003
17.000	0.309	0.003	38.000	0.309	0.003
18.000	0.304	0.003	39.000	0.308	0.003
19.000	0.298	0.004	40.000	0.306	0.003
20.000	0.302	0.004	41.000	0.307	0.003
21.000	0.305	0.003	42.000	0.305	0.003
22.000	0.305	0.003	43.000	0.304	0.003
23.000	0.304	0.003	44.000	0.303	0.004
24.000	0.306	0.003	45.000	0.302	0.004
25.000	0.307	0.003	46.000	0.299	0.004
26.000	0.305	0.003	47.000	0.301	0.004
27.000	0.309	0.003	48.000	0.304	0.003
28.000	0.310	0.003	49.000	0.301	0.004
29.000	0.310	0.003	50.000	0.298	0.004
30.000	0.308	0.003			
**Transitivity**					
**density**	**coefficient**	**p-value**	**density**	**coefficient**	**p-value**
23.000	0.353	0.001	37.000	0.335	0.001
24.000	0.352	0.001	38.000	0.337	0.001
25.000	0.353	0.001	39.000	0.334	0.001
26.000	0.351	0.001	40.000	0.332	0.001
27.000	0.352	0.001	41.000	0.330	0.001

Continued on next page

TABLE S6 – continued from previous page

28.000	0.351	0.001	42.000	0.327	0.002
29.000	0.350	0.001	43.000	0.325	0.002
30.000	0.348	0.001	44.000	0.323	0.002
31.000	0.346	0.001	45.000	0.322	0.002
32.000	0.346	0.001	46.000	0.318	0.002
33.000	0.344	0.001	47.000	0.320	0.002
34.000	0.344	0.001	48.000	0.321	0.002
35.000	0.341	0.001	49.000	0.318	0.002
36.000	0.340	0.001	50.000	0.315	0.002

**Modularity**					
**density**	**coefficient**	**p-value**	**density**	**coefficient**	**p-value**
25.000	-0.304	0.003	38.000	-0.340	0.001
26.000	-0.286	0.006	39.000	-0.326	0.002
27.000	-0.270	0.010	40.000	-0.315	0.002
28.000	-0.273	0.009	41.000	-0.305	0.003
29.000	-0.308	0.003	42.000	-0.306	0.003
30.000	-0.307	0.003	43.000	-0.313	0.003
31.000	-0.363	0.000	44.000	-0.312	0.003
32.000	-0.360	0.000	45.000	-0.308	0.003
33.000	-0.305	0.003	46.000	-0.311	0.003
34.000	-0.340	0.001	47.000	-0.312	0.003
35.000	-0.340	0.001	48.000	-0.313	0.002
36.000	-0.347	0.001	49.000	-0.313	0.003
37.000	-0.316	0.002	50.000	-0.307	0.003

TABLE S7: Correlation analysis between various global measures calculated at lag of 6 and the UPDRS-III motor scores.

Global efficiency					
**density**	**coefficient**	**p-value**	**density**	**coefficient**	**p-value**
1.000	0.307	0.003	6.000	0.357	0.001
2.000	0.368	0.000	7.000	0.353	0.001
3.000	0.392	0.000	8.000	0.347	0.001
4.000	0.368	0.000	9.000	0.331	0.001
5.000	0.363	0.000	10.000	0.321	0.002
**Local efficiency**					
**density**	**coefficient**	**p-value**	**density**	**coefficient**	**p-value**
7.000	0.255	0.015	14.000	0.342	0.001
8.000	0.308	0.003	15.000	0.338	0.001
9.000	0.335	0.001	16.000	0.327	0.002
10.000	0.342	0.001	17.000	0.317	0.002
11.000	0.344	0.001	18.000	0.306	0.003
12.000	0.341	0.001	19.000	0.299	0.004
13.000	0.353	0.001	20.000	0.291	0.005
**Clustering**					
**density**	**coefficient**	**p-value**	**density**	**coefficient**	**p-value**
9.000	0.325	0.002	30.000	0.332	0.001
10.000	0.316	0.002	31.000	0.329	0.001
11.000	0.315	0.002	32.000	0.330	0.001
12.000	0.313	0.003	33.000	0.329	0.001
13.000	0.318	0.002	34.000	0.330	0.001
14.000	0.320	0.002	35.000	0.326	0.002
15.000	0.325	0.002	36.000	0.328	0.001
16.000	0.326	0.002	37.000	0.328	0.001

Continued on next page

TABLE S7 – continued from previous page

17.000	0.329	0.001	38.000	0.329	0.001
18.000	0.325	0.002	39.000	0.328	0.001
19.000	0.329	0.001	40.000	0.326	0.002
20.000	0.329	0.001	41.000	0.324	0.002
21.000	0.334	0.001	42.000	0.325	0.002
22.000	0.331	0.001	43.000	0.326	0.002
23.000	0.332	0.001	44.000	0.322	0.002
24.000	0.330	0.001	45.000	0.322	0.002
25.000	0.328	0.002	46.000	0.324	0.002
26.000	0.332	0.001	47.000	0.322	0.002
27.000	0.330	0.001	48.000	0.323	0.002
28.000	0.335	0.001	49.000	0.326	0.002
29.000	0.335	0.001	50.000	0.324	0.002
**Transitivity**					
**density**	**coefficient**	**p-value**	**density**	**coefficient**	**p-value**
9.000	0.348	0.001	30.000	0.341	0.001
10.000	0.350	0.001	31.000	0.339	0.001
11.000	0.348	0.001	32.000	0.341	0.001
12.000	0.345	0.001	33.000	0.339	0.001
13.000	0.343	0.001	34.000	0.339	0.001
14.000	0.342	0.001	35.000	0.335	0.001
15.000	0.344	0.001	36.000	0.337	0.001
16.000	0.347	0.001	37.000	0.337	0.001
17.000	0.346	0.001	38.000	0.338	0.001
18.000	0.347	0.001	39.000	0.337	0.001
19.000	0.347	0.001	40.000	0.336	0.001
20.000	0.346	0.001	41.000	0.335	0.001

Continued on next page

TABLE S7 – continued from previous page

21.000	0.349	0.001	42.000	0.335	0.001
22.000	0.346	0.001	43.000	0.336	0.001
23.000	0.347	0.001	44.000	0.332	0.001
24.000	0.345	0.001	45.000	0.332	0.001
25.000	0.342	0.001	46.000	0.334	0.001
26.000	0.344	0.001	47.000	0.333	0.001
27.000	0.341	0.001	48.000	0.334	0.001
28.000	0.344	0.001	49.000	0.337	0.001
29.000	0.343	0.001	50.000	0.335	0.001

**Modularity**					
**density**	**coefficient**	**p-value**	**density**	**coefficient**	**p-value**
25.000	-0.312	0.003	38.000	-0.325	0.002
26.000	-0.337	0.001	39.000	-0.326	0.002
27.000	-0.376	0.000	40.000	-0.310	0.003
28.000	-0.295	0.005	41.000	-0.308	0.003
29.000	-0.278	0.008	42.000	-0.316	0.002
30.000	-0.347	0.001	43.000	-0.316	0.002
31.000	-0.286	0.006	44.000	-0.308	0.003
32.000	-0.286	0.006	45.000	-0.314	0.002
33.000	-0.312	0.003	46.000	-0.319	0.002
34.000	-0.294	0.005	47.000	-0.321	0.002
35.000	-0.284	0.006	48.000	-0.329	0.001
36.000	-0.312	0.003	49.000	-0.329	0.001
37.000	-0.311	0.003	50.000	-0.328	0.001

TABLE S8: Correlation analysis between various global measures calculated at lag of 7 and the UPDRS-III motor scores.

**Global efficiency**					
**density**	**coefficient**	**p-value**	**density**	**coefficient**	**p-value**
2.000	0.280	0.007	7.000	0.263	0.012
3.000	0.301	0.004	8.000	0.263	0.012
4.000	0.313	0.003	9.000	0.239	0.022
5.000	0.310	0.003	10.000	0.246	0.019
6.000	0.287	0.006	11.000	0.233	0.026
**Local efficiency**					
**density**	**coefficient**	**p-value**	**density**	**coefficient**	**p-value**
8.000	0.213	0.042	13.000	0.262	0.012
9.000	0.232	0.027	14.000	0.264	0.012
10.000	0.259	0.013	15.000	0.252	0.016
11.000	0.268	0.010	16.000	0.246	0.019
12.000	0.266	0.011	17.000	0.240	0.022
**Clustering**					
**density**	**coefficient**	**p-value**	**density**	**coefficient**	**p-value**
6.000	0.238	0.023	29.000	0.286	0.006
7.000	0.241	0.022	30.000	0.287	0.006
8.000	0.267	0.010	31.000	0.288	0.006
9.000	0.261	0.013	32.000	0.287	0.006
10.000	0.266	0.011	33.000	0.288	0.006
11.000	0.269	0.010	34.000	0.287	0.006
12.000	0.266	0.011	35.000	0.288	0.006
13.000	0.274	0.009	36.000	0.286	0.006
14.000	0.269	0.010	37.000	0.283	0.007
15.000	0.261	0.012	38.000	0.285	0.006

Continued on next page

TABLE S8 – continued from previous page

16.000	0.269	0.010	39.000	0.285	0.006
17.000	0.261	0.013	40.000	0.286	0.006
18.000	0.269	0.010	41.000	0.285	0.006
19.000	0.274	0.009	42.000	0.283	0.007
20.000	0.282	0.007	43.000	0.286	0.006
21.000	0.287	0.006	44.000	0.283	0.007
22.000	0.287	0.006	45.000	0.287	0.006
23.000	0.287	0.006	46.000	0.283	0.007
24.000	0.286	0.006	47.000	0.283	0.007
25.000	0.287	0.006	48.000	0.283	0.007
26.000	0.288	0.006	49.000	0.283	0.006
27.000	0.283	0.007	50.000	0.283	0.007
28.000	0.287	0.006			
**Transitivity**					
**density**	**coefficient**	**p-value**	**density**	**coefficient**	**p-value**
10.000	0.212	0.043	31.000	0.287	0.006
11.000	0.218	0.038	32.000	0.287	0.006
12.000	0.216	0.039	33.000	0.289	0.005
13.000	0.231	0.028	34.000	0.289	0.005
14.000	0.239	0.022	35.000	0.291	0.005
15.000	0.237	0.024	36.000	0.290	0.005
16.000	0.248	0.018	37.000	0.287	0.006
17.000	0.247	0.018	38.000	0.290	0.005
18.000	0.255	0.015	39.000	0.290	0.005
19.000	0.263	0.012	40.000	0.292	0.005
20.000	0.272	0.009	41.000	0.291	0.005
21.000	0.277	0.008	42.000	0.289	0.005

Continued on next page

TABLE S8 – continued from previous page

22.000	0.278	0.008	43.000	0.293	0.005
23.000	0.278	0.008	44.000	0.290	0.005
24.000	0.278	0.008	45.000	0.294	0.005
25.000	0.281	0.007	46.000	0.291	0.005
26.000	0.282	0.007	47.000	0.291	0.005
27.000	0.279	0.007	48.000	0.292	0.005
28.000	0.285	0.006	49.000	0.293	0.005
29.000	0.285	0.006	50.000	0.293	0.005
30.000	0.286	0.006			
**Modularity**					
**density**	**coefficient**	**p-value**	**density**	**coefficient**	**p-value**
23.000	-0.312	0.003	37.000	-0.301	0.004
24.000	-0.411	0.000	38.000	-0.303	0.004
25.000	-0.383	0.000	39.000	-0.316	0.002
26.000	-0.367	0.000	40.000	-0.310	0.003
27.000	-0.340	0.001	41.000	-0.300	0.004
28.000	-0.352	0.001	42.000	-0.293	0.005
29.000	-0.372	0.000	43.000	-0.295	0.005
30.000	-0.375	0.000	44.000	-0.296	0.004
31.000	-0.351	0.001	45.000	-0.295	0.005
32.000	-0.334	0.001	46.000	-0.292	0.005
33.000	-0.331	0.001	47.000	-0.291	0.005
34.000	-0.322	0.002	48.000	-0.291	0.005
35.000	-0.318	0.002	49.000	-0.295	0.005
36.000	-0.307	0.003	50.000	-0.295	0.005

TABLE S9: Correlation analysis between various global measures calculated at lag of 1 and the letter number sequencing test scores.

Transitivity					
**density**	**coefficient**	**p-value**	**density**	**coefficient**	**p-value**
20.000	-0.229	0.029	36.000	-0.248	0.018
21.000	-0.230	0.028	37.000	-0.247	0.018
22.000	-0.234	0.026	38.000	-0.248	0.018
23.000	-0.236	0.024	39.000	-0.249	0.017
24.000	-0.234	0.025	40.000	-0.250	0.017
25.000	-0.239	0.023	41.000	-0.247	0.018
26.000	-0.237	0.024	42.000	-0.246	0.019
27.000	-0.239	0.023	43.000	-0.245	0.019
28.000	-0.241	0.022	44.000	-0.244	0.020
29.000	-0.242	0.021	45.000	-0.242	0.021
30.000	-0.242	0.021	46.000	-0.242	0.021
31.000	-0.245	0.019	47.000	-0.242	0.021
32.000	-0.244	0.020	48.000	-0.243	0.020
33.000	-0.248	0.018	49.000	-0.244	0.020
34.000	-0.246	0.019	50.000	-0.245	0.019
35.000	-0.248	0.018			

TABLE S10: Correlation analysis between various global measures calculated at lag of 2 and the letter number sequencing test scores.

Global efficiency					
**density**	**coefficient**	**p-value**	**density**	**coefficient**	**p-value**
12.000	-0.259	0.013	25.000	-0.249	0.017
13.000	-0.264	0.011	26.000	-0.245	0.019
14.000	-0.260	0.013	27.000	-0.242	0.021
15.000	-0.262	0.012	28.000	-0.247	0.018
16.000	-0.263	0.012	29.000	-0.247	0.018
17.000	-0.266	0.011	30.000	-0.240	0.022
18.000	-0.262	0.012	31.000	-0.238	0.023
19.000	-0.263	0.012	32.000	-0.234	0.026
20.000	-0.265	0.011	33.000	-0.233	0.026
21.000	-0.265	0.011	34.000	-0.234	0.025
22.000	-0.265	0.011	35.000	-0.228	0.030
23.000	-0.257	0.014	36.000	-0.228	0.030
24.000	-0.257	0.014	37.000	-0.228	0.030
**Local efficiency**					
**density**	**coefficient**	**p-value**	**density**	**coefficient**	**p-value**
11.000	-0.235	0.025	25.000	-0.258	0.013
12.000	-0.247	0.018	26.000	-0.252	0.016
13.000	-0.274	0.009	27.000	-0.248	0.018
14.000	-0.276	0.008	28.000	-0.249	0.017
15.000	-0.287	0.006	29.000	-0.250	0.017
16.000	-0.284	0.006	30.000	-0.239	0.023
17.000	-0.287	0.006	31.000	-0.235	0.025
18.000	-0.286	0.006	32.000	-0.232	0.027
19.000	-0.285	0.006	33.000	-0.231	0.027

Continued on next page

TABLE S10 – continued from previous page

20.000	-0.286	0.006	34.000	-0.231	0.027
21.000	-0.285	0.006	35.000	-0.228	0.030
22.000	-0.280	0.007	36.000	-0.227	0.030
23.000	-0.270	0.010	37.000	-0.227	0.031
24.000	-0.265	0.011			

Clustering

density	coefficient	p-value	density	coefficient	p-value
28.000	-0.231	0.028	40.000	-0.251	0.016
29.000	-0.231	0.028	41.000	-0.252	0.016
30.000	-0.230	0.028	42.000	-0.256	0.014
31.000	-0.234	0.026	43.000	-0.257	0.014
32.000	-0.235	0.025	44.000	-0.257	0.014
33.000	-0.237	0.024	45.000	-0.259	0.013
34.000	-0.239	0.022	46.000	-0.260	0.013
35.000	-0.240	0.022	47.000	-0.263	0.012
36.000	-0.244	0.020	48.000	-0.265	0.011
37.000	-0.246	0.019	49.000	-0.266	0.011
38.000	-0.248	0.018	50.000	-0.265	0.011
39.000	-0.249	0.017			

Transitivity

density	coefficient	p-value	density	coefficient	p-value
22.000	-0.208	0.048	37.000	-0.249	0.017
23.000	-0.211	0.045	38.000	-0.251	0.017
24.000	-0.213	0.042	39.000	-0.253	0.015
25.000	-0.219	0.037	40.000	-0.256	0.014
26.000	-0.223	0.034	41.000	-0.257	0.014
27.000	-0.226	0.031	42.000	-0.261	0.012

Continued on next page

TABLE S10 – continued from previous page

28.000	-0.230	0.028	43.000	-0.261	0.012
29.000	-0.232	0.027	44.000	-0.261	0.012
30.000	-0.234	0.026	45.000	-0.263	0.012
31.000	-0.237	0.024	46.000	-0.263	0.012
32.000	-0.238	0.023	47.000	-0.266	0.011
33.000	-0.240	0.022	48.000	-0.268	0.010
34.000	-0.243	0.020	49.000	-0.269	0.010
35.000	-0.245	0.019	50.000	-0.268	0.010
36.000	-0.247	0.018			

**Modularity**					
**density**	**coefficient**	**p-value**	**density**	**coefficient**	**p-value**
34.000	0.236	0.024	43.000	0.266	0.011
35.000	0.219	0.037	44.000	0.268	0.010
36.000	0.238	0.023	45.000	0.266	0.011
37.000	0.242	0.021	46.000	0.272	0.009
38.000	0.237	0.024	47.000	0.275	0.008
39.000	0.251	0.016	48.000	0.281	0.007
40.000	0.255	0.015	49.000	0.283	0.007
41.000	0.257	0.014	50.000	0.281	0.007
42.000	0.269	0.010			

TABLE S11: Correlation analysis between various global measures calculated at lag of 3 and the letter number sequencing test scores.

Clustering					
**density**	**coefficient**	**p-value**	**density**	**coefficient**	**p-value**
16.000	-0.211	0.045	38.000	-0.255	0.015
17.000	-0.222	0.034	39.000	-0.257	0.014
18.000	-0.224	0.033	40.000	-0.257	0.014
19.000	-0.225	0.032	41.000	-0.260	0.013
20.000	-0.227	0.030	42.000	-0.265	0.011
21.000	-0.230	0.029	43.000	-0.262	0.012
22.000	-0.230	0.028	44.000	-0.263	0.012
23.000	-0.239	0.022	45.000	-0.266	0.011
24.000	-0.244	0.020	46.000	-0.267	0.010
25.000	-0.245	0.019	47.000	-0.267	0.010
26.000	-0.244	0.020	48.000	-0.267	0.011
27.000	-0.242	0.021	49.000	-0.267	0.010
28.000	-0.244	0.020	50.000	-0.269	0.010
37.000	-0.255	0.015			
**Transitivity**					
**density**	**coefficient**	**p-value**	**density**	**coefficient**	**p-value**
25.000	-0.224	0.033	38.000	-0.254	0.015
26.000	-0.225	0.032	39.000	-0.256	0.014
27.000	-0.229	0.029	40.000	-0.257	0.014
28.000	-0.233	0.026	41.000	-0.259	0.013
29.000	-0.235	0.025	42.000	-0.265	0.011
30.000	-0.238	0.023	43.000	-0.262	0.012
31.000	-0.240	0.022	44.000	-0.263	0.012
32.000	-0.242	0.021	45.000	-0.266	0.011

Continued on next page

TABLE S11 – continued from previous page

33.000	-0.243	0.020	46.000	-0.267	0.011
34.000	-0.248	0.018	47.000	-0.268	0.010
35.000	-0.249	0.017	48.000	-0.268	0.010
36.000	-0.250	0.017	49.000	-0.268	0.010
37.000	-0.254	0.015	50.000	-0.270	0.010

TABLE S12: Correlation analysis between various global measures calculated at lag of 4 and the letter number sequencing test scores.

Global efficiency					
**density**	**coefficient**	**p-value**	**density**	**coefficient**	**p-value**
9.000	-0.308	0.003	25.000	-0.276	0.008
10.000	-0.303	0.004	26.000	-0.276	0.008
11.000	-0.312	0.003	27.000	-0.268	0.010
12.000	-0.312	0.003	28.000	-0.266	0.011
13.000	-0.319	0.002	29.000	-0.271	0.009
14.000	-0.314	0.002	30.000	-0.272	0.009
15.000	-0.305	0.003	31.000	-0.267	0.011
16.000	-0.303	0.004	32.000	-0.264	0.012
17.000	-0.302	0.004	33.000	-0.261	0.013
18.000	-0.294	0.005	34.000	-0.253	0.015
19.000	-0.291	0.005	35.000	-0.249	0.017
20.000	-0.285	0.006	36.000	-0.243	0.020
21.000	-0.274	0.009	37.000	-0.246	0.019
22.000	-0.279	0.007	38.000	-0.243	0.020
23.000	-0.279	0.007	39.000	-0.242	0.021
24.000	-0.280	0.007			
**Local efficiency**					
**density**	**coefficient**	**p-value**	**density**	**coefficient**	**p-value**
11.000	-0.227	0.030	26.000	-0.281	0.007
12.000	-0.250	0.017	27.000	-0.270	0.010
13.000	-0.283	0.007	28.000	-0.267	0.011
14.000	-0.301	0.004	29.000	-0.269	0.010
15.000	-0.309	0.003	30.000	-0.272	0.009
16.000	-0.313	0.003	31.000	-0.268	0.010

Continued on next page

17.000	-0.312	0.003	32.000	-0.267	0.011
18.000	-0.309	0.003	33.000	-0.264	0.012
19.000	-0.304	0.003	34.000	-0.256	0.014
20.000	-0.303	0.003	35.000	-0.253	0.015
21.000	-0.292	0.005	36.000	-0.249	0.017
22.000	-0.298	0.004	37.000	-0.253	0.016
23.000	-0.296	0.004	38.000	-0.249	0.017
24.000	-0.292	0.005	39.000	-0.246	0.019
25.000	-0.286	0.006	40.000	-0.251	0.017

Clustering

density	coefficient	p-value	density	coefficient	p-value
11.000	-0.213	0.043	31.000	-0.274	0.008
12.000	-0.224	0.033	32.000	-0.275	0.008
13.000	-0.236	0.024	33.000	-0.277	0.008
14.000	-0.242	0.021	34.000	-0.276	0.008
15.000	-0.251	0.016	35.000	-0.274	0.009
16.000	-0.254	0.015	36.000	-0.275	0.008
17.000	-0.263	0.012	37.000	-0.276	0.008
18.000	-0.257	0.014	38.000	-0.275	0.008
19.000	-0.260	0.013	39.000	-0.279	0.007
20.000	-0.257	0.014	40.000	-0.280	0.007
21.000	-0.255	0.015	41.000	-0.281	0.007
22.000	-0.265	0.011	42.000	-0.280	0.007
23.000	-0.269	0.010	43.000	-0.281	0.007
24.000	-0.270	0.010	44.000	-0.281	0.007
25.000	-0.267	0.010	45.000	-0.279	0.007
26.000	-0.272	0.009	46.000	-0.280	0.007

Continued on next page

TABLE S12 – continued from previous page

27.000	-0.269	0.010	47.000	-0.280	0.007
28.000	-0.268	0.010	48.000	-0.281	0.007
29.000	-0.273	0.009	49.000	-0.280	0.007
30.000	-0.274	0.009	50.000	-0.282	0.007

Transitivity

density	coefficient	p-value	density	coefficient	p-value
23.000	-0.254	0.015	37.000	-0.278	0.008
24.000	-0.257	0.014	38.000	-0.277	0.008
25.000	-0.257	0.014	39.000	-0.281	0.007
26.000	-0.263	0.012	40.000	-0.281	0.007
27.000	-0.263	0.012	41.000	-0.282	0.007
28.000	-0.263	0.012	42.000	-0.281	0.007
29.000	-0.268	0.010	43.000	-0.283	0.007
30.000	-0.269	0.010	44.000	-0.284	0.006
31.000	-0.271	0.009	45.000	-0.282	0.007
32.000	-0.271	0.009	46.000	-0.283	0.007
33.000	-0.274	0.009	47.000	-0.283	0.007
34.000	-0.275	0.008	48.000	-0.284	0.006
35.000	-0.274	0.008	49.000	-0.283	0.007
36.000	-0.277	0.008	50.000	-0.285	0.006

Modularity

density	coefficient	p-value	density	coefficient	p-value
32.000	0.227	0.030	42.000	0.280	0.007
33.000	0.233	0.026	43.000	0.285	0.006
34.000	0.256	0.014	44.000	0.290	0.005
35.000	0.252	0.016	45.000	0.290	0.005
36.000	0.269	0.010	46.000	0.287	0.006

Continued on next page

TABLE S12 – continued from previous page

37.000	0.275	0.008	47.000	0.286	0.006
38.000	0.275	0.008	48.000	0.288	0.006
39.000	0.288	0.006	49.000	0.287	0.006
40.000	0.281	0.007	50.000	0.283	0.007
41.000	0.284	0.006			

TABLE S13: Correlation analysis between various global measures calculated at lag of 5 and the letter number sequencing test scores.

Global efficiency					
**density**	**coefficient**	**p-value**	**density**	**coefficient**	**p-value**
3.000	-0.214	0.042	12.000	-0.268	0.010
4.000	-0.211	0.045	13.000	-0.262	0.012
5.000	-0.235	0.025	14.000	-0.262	0.012
6.000	-0.247	0.018	15.000	-0.242	0.021
7.000	-0.281	0.007	16.000	-0.246	0.019
8.000	-0.275	0.008	17.000	-0.257	0.014
9.000	-0.276	0.008	18.000	-0.248	0.018
10.000	-0.276	0.008	19.000	-0.243	0.020
11.000	-0.273	0.009	20.000	-0.235	0.025
**Local efficiency**					
**density**	**coefficient**	**p-value**	**density**	**coefficient**	**p-value**
14.000	-0.236	0.024	21.000	-0.259	0.013
15.000	-0.239	0.022	22.000	-0.258	0.014
16.000	-0.247	0.018	23.000	-0.249	0.017
17.000	-0.259	0.013	24.000	-0.249	0.017
18.000	-0.263	0.012	25.000	-0.248	0.018
19.000	-0.265	0.011	26.000	-0.245	0.019
20.000	-0.260	0.013	27.000	-0.242	0.021
**Clustering**					
**density**	**coefficient**	**p-value**	**density**	**coefficient**	**p-value**
17.000	-0.214	0.041	34.000	-0.256	0.014
18.000	-0.225	0.032	35.000	-0.256	0.014
19.000	-0.222	0.034	36.000	-0.257	0.014
20.000	-0.227	0.030	37.000	-0.255	0.015

Continued on next page

TABLE S13 – continued from previous page

21.000	-0.231	0.028	38.000	-0.259	0.013
22.000	-0.234	0.026	39.000	-0.260	0.013
23.000	-0.230	0.028	40.000	-0.260	0.013
24.000	-0.236	0.024	41.000	-0.260	0.013
25.000	-0.245	0.019	42.000	-0.258	0.013
26.000	-0.245	0.019	43.000	-0.260	0.013
27.000	-0.247	0.018	44.000	-0.257	0.014
28.000	-0.246	0.019	45.000	-0.259	0.013
29.000	-0.250	0.017	46.000	-0.260	0.013
30.000	-0.254	0.015	47.000	-0.260	0.013
31.000	-0.256	0.014	48.000	-0.260	0.013
32.000	-0.256	0.014	49.000	-0.260	0.013
33.000	-0.255	0.015	50.000	-0.260	0.013

Transitivity

density	coefficient	p-value	density	coefficient	p-value
23.000	-0.216	0.039	37.000	-0.250	0.017
24.000	-0.221	0.035	38.000	-0.254	0.015
25.000	-0.229	0.029	39.000	-0.255	0.015
26.000	-0.230	0.028	40.000	-0.256	0.014
27.000	-0.233	0.026	41.000	-0.257	0.014
28.000	-0.233	0.026	42.000	-0.256	0.014
29.000	-0.238	0.023	43.000	-0.258	0.014
30.000	-0.241	0.021	44.000	-0.257	0.014
31.000	-0.245	0.019	45.000	-0.258	0.014
32.000	-0.246	0.019	46.000	-0.260	0.013
33.000	-0.247	0.018	47.000	-0.260	0.013
34.000	-0.248	0.018	48.000	-0.261	0.012

Continued on next page

TABLE S13 – continued from previous page

35.000	-0.249	0.017	49.000	-0.261	0.013
36.000	-0.250	0.017	50.000	-0.260	0.013

**Modularity**					
**density**	**coefficient**	**p-value**	**density**	**coefficient**	**p-value**
39.000	0.248	0.018	45.000	0.262	0.012
40.000	0.249	0.017	46.000	0.264	0.011
41.000	0.259	0.013	47.000	0.265	0.011
42.000	0.267	0.011	48.000	0.264	0.011
43.000	0.264	0.012	49.000	0.261	0.013
44.000	0.264	0.011	50.000	0.261	0.012

TABLE S14: Correlation analysis between various global measures calculated at lag of 6 and the letter number sequencing test scores.

**Global efficiency**					
**density**	**coefficient**	**p-value**	**density**	**coefficient**	**p-value**
4.000	-0.235	0.025	8.000	-0.259	0.013
5.000	-0.239	0.022	9.000	-0.264	0.012
6.000	-0.242	0.021	10.000	-0.261	0.012
7.000	-0.253	0.016			
**Modularity**					
**density**	**coefficient**	**p-value**	**density**	**coefficient**	**p-value**
25.000	0.235	0.025	35.000	0.259	0.013
26.000	0.304	0.003	36.000	0.275	0.008
27.000	0.242	0.021	37.000	0.268	0.010
28.000	0.234	0.026	38.000	0.258	0.014
29.000	0.253	0.015	39.000	0.248	0.018
30.000	0.334	0.001	40.000	0.246	0.019
31.000	0.288	0.006	41.000	0.234	0.025
32.000	0.299	0.004	42.000	0.231	0.028
33.000	0.277	0.008	43.000	0.227	0.031
34.000	0.274	0.009			

TABLE S15: Correlation analysis between various global measures calculated at lag of 7 and the letter number sequencing test scores.

**Modularity**					
**density**	**coefficient**	**p-value**	**density**	**coefficient**	**p-value**
24.000	0.312	0.003	29.000	0.305	0.003
25.000	0.287	0.006	30.000	0.297	0.004
26.000	0.332	0.001	31.000	0.274	0.009
27.000	0.312	0.003	32.000	0.274	0.009
28.000	0.269	0.010	33.000	0.254	0.015

TABLE S16: Correlation analysis between various global measures calculated at lag of 1 and the symbol digit modalities test scores.

Clustering					
**density**	**coefficient**	**p-value**	**density**	**coefficient**	**p-value**
28.000	-0.222	0.034	40.000	-0.241	0.021
29.000	-0.225	0.032	41.000	-0.236	0.024
30.000	-0.226	0.031	42.000	-0.237	0.024
31.000	-0.230	0.028	43.000	-0.238	0.023
32.000	-0.228	0.029	44.000	-0.238	0.023
33.000	-0.229	0.029	45.000	-0.239	0.023
34.000	-0.229	0.029	46.000	-0.239	0.022
35.000	-0.229	0.029	47.000	-0.240	0.022
36.000	-0.231	0.027	48.000	-0.243	0.020
37.000	-0.233	0.026	49.000	-0.242	0.021
38.000	-0.235	0.025	50.000	-0.244	0.020
39.000	-0.238	0.023			

Transitivity					
**density**	**coefficient**	**p-value**	**density**	**coefficient**	**p-value**
25.000	-0.215	0.041	38.000	-0.241	0.021
26.000	-0.216	0.040	39.000	-0.245	0.019
27.000	-0.219	0.037	40.000	-0.247	0.018
28.000	-0.223	0.034	41.000	-0.242	0.021
29.000	-0.228	0.030	42.000	-0.242	0.021
30.000	-0.228	0.030	43.000	-0.242	0.021
31.000	-0.232	0.027	44.000	-0.243	0.020
32.000	-0.232	0.027	45.000	-0.244	0.020
33.000	-0.234	0.026	46.000	-0.245	0.019
34.000	-0.235	0.025	47.000	-0.245	0.019

Continued on next page

TABLE S16 – continued from previous page

35.000	-0.236	0.025	48.000	-0.248	0.018
36.000	-0.239	0.023	49.000	-0.248	0.018
37.000	-0.240	0.022	50.000	-0.250	0.017

TABLE S17: Correlation analysis between various global measures calculated at lag of 5 and the Hopkins verbal learning test-revised test scores.

**Global efficiency**					
**density**	**coefficient**	**p-value**	**density**	**coefficient**	**p-value**
7.000	-0.221	0.035	14.000	-0.234	0.025
8.000	-0.224	0.033	15.000	-0.225	0.032
9.000	-0.217	0.038	16.000	-0.227	0.030
10.000	-0.224	0.033	17.000	-0.217	0.039
11.000	-0.227	0.031	18.000	-0.211	0.045
12.000	-0.232	0.027	19.000	-0.210	0.045
13.000	-0.237	0.024			

TABLE S18: Correlation analysis between various global measures calculated at lag of 7 and the Benton's judgment of line orientation test scores.

Global efficiency					
**density**	**coefficient**	**p-value**	**density**	**coefficient**	**p-value**
5.000	0.221	0.035	9.000	0.236	0.025
6.000	0.219	0.037	10.000	0.235	0.025
7.000	0.227	0.031	11.000	0.229	0.029
8.000	0.226	0.031			
**Local efficiency**					
**density**	**coefficient**	**p-value**	**density**	**coefficient**	**p-value**
8.000	0.232	0.027	13.000	0.257	0.014
9.000	0.236	0.025	14.000	0.256	0.015
10.000	0.243	0.020	15.000	0.260	0.013
11.000	0.242	0.021	16.000	0.269	0.010
12.000	0.244	0.020	17.000	0.276	0.008
**Clustering**					
**density**	**coefficient**	**p-value**	**density**	**coefficient**	**p-value**
7.000	0.230	0.028	29.000	0.236	0.024
8.000	0.220	0.036	30.000	0.239	0.023
9.000	0.212	0.044	31.000	0.239	0.023
10.000	0.227	0.031	32.000	0.243	0.020
11.000	0.222	0.035	33.000	0.238	0.023
12.000	0.226	0.032	34.000	0.237	0.024
13.000	0.228	0.030	35.000	0.241	0.022
14.000	0.228	0.029	36.000	0.242	0.021
15.000	0.233	0.026	37.000	0.246	0.019
16.000	0.232	0.027	38.000	0.245	0.019
17.000	0.235	0.025	39.000	0.243	0.020

Continued on next page

TABLE S18 – continued from previous page

18.000	0.234	0.026	40.000	0.245	0.019
19.000	0.242	0.021	41.000	0.247	0.018
20.000	0.241	0.021	42.000	0.247	0.018
21.000	0.243	0.020	43.000	0.248	0.018
22.000	0.246	0.019	44.000	0.249	0.018
23.000	0.242	0.021	45.000	0.248	0.018
24.000	0.241	0.021	46.000	0.247	0.019
25.000	0.234	0.025	47.000	0.247	0.018
26.000	0.239	0.023	48.000	0.249	0.017
27.000	0.239	0.023	49.000	0.250	0.017
28.000	0.234	0.025	50.000	0.252	0.016
**Transitivity**					
**density**	**coefficient**	**p-value**	**density**	**coefficient**	**p-value**
13.000	0.219	0.037	32.000	0.246	0.019
14.000	0.224	0.032	33.000	0.243	0.020
15.000	0.227	0.030	34.000	0.243	0.020
16.000	0.231	0.027	35.000	0.246	0.019
17.000	0.234	0.025	36.000	0.245	0.019
18.000	0.236	0.024	37.000	0.248	0.018
19.000	0.241	0.021	38.000	0.248	0.018
20.000	0.243	0.020	39.000	0.246	0.019
21.000	0.244	0.020	40.000	0.248	0.018
22.000	0.248	0.018	41.000	0.249	0.017
23.000	0.245	0.019	42.000	0.249	0.017
24.000	0.244	0.020	43.000	0.249	0.017
25.000	0.242	0.021	44.000	0.249	0.017
26.000	0.245	0.019	45.000	0.249	0.018

Continued on next page

TABLE S18 – continued from previous page

27.000	0.245	0.019	46.000	0.248	0.018
28.000	0.243	0.020	47.000	0.248	0.018
29.000	0.244	0.020	48.000	0.250	0.017
30.000	0.244	0.020	49.000	0.250	0.017
31.000	0.244	0.020	50.000	0.251	0.016

VII. PARTICIPANTS - DIVISION INTO SUBGROUPS BASED ON MEDICATION STATUS.

	**MED (n = 64)**	**non-MED (n = 31)**	**MED vs non-MED (p value)**
**Age (years)**	68.7 (10.4)	66.6 (10.6)	0.38
**Gender (% male)**	76.6%	51.6%	0.02
**Education (years)**	14.7 (2.8)	16.5 (2.6)	0.01
**UPDRS-III test scores**	22.0 (11.3)	19.7 (9.2)	0.33
**HY stage (1-2)**	16 - 48	11 - 20	–
**LEDD (dose)**	405.3 (207.0)	–	–
**Cognitive status (% MCI)**	20.3%	19.4%	–

TABLE S19. **Characteristics of the sample - division into subgroups based on the medication status**. Means are followed by standard deviation in parentesis. Permutation tests with 10000 permutations were used to compare groups for age, gender, education and UPDRS-III scores. MED, Parkinson's disease patients on levodopa medication; non-MED, Parkinson's disease patients off levodopa medication; UPDRS-III, Unified Parkinson's disease rating scale–Part III; HY stage, Hoehn and Yahr stage; LEDD, levodopa equivalent dose; MCI, mild cognitive impairment.

VIII. PARTICIPANTS - DIVISION INTO SUBGROUPS BASED ON COGNITIVE STATUS.

	**PDCN (n = 76)**	**PDMCI (n = 19)**	**PDCN vs PDMCI (p value)**
**Age (years)**	67.3 (10.6)	70.9 (9.4)	0.18
**Gender (% male)**	64.5%	84.2%	0.11
**Education (years)**	15.4 (2.9)	14.8 (2.9)	0.48
**UPDRS-III test scores**	20.7 (10.5)	23.7 (11.2)	0.28
**HY stage (1-2)**	22 - 54	5 - 14	–
**LEDD (dose)**	396.4 (199.2)	440.3 (240.9)	–
**LEDD (% medicated)**	67.1%	68.4%	–

TABLE S20. **Characteristics of the sample - division into subgroups based on cognitive status**. Means are followed by standard deviation in parentesis. Permutation tests with 10000 permutations were used to compare groups for age, gender, education and UPDRS-III scores. PDCN, Parkinson's disease cognitively normal; PDMCI, Parkinson's disease with mild cognitive impairment; UPDRS-III, Unified Parkinson's disease rating scale–Part III; HY stage, Hoehn and Yahr stage; LEDD, levodopa equivalent dose.

IX. WEIGHTED NETWORK ANALYSIS: DIFFERENCES IN GLOBAL TOPOLOGY

A. Lags 1-4

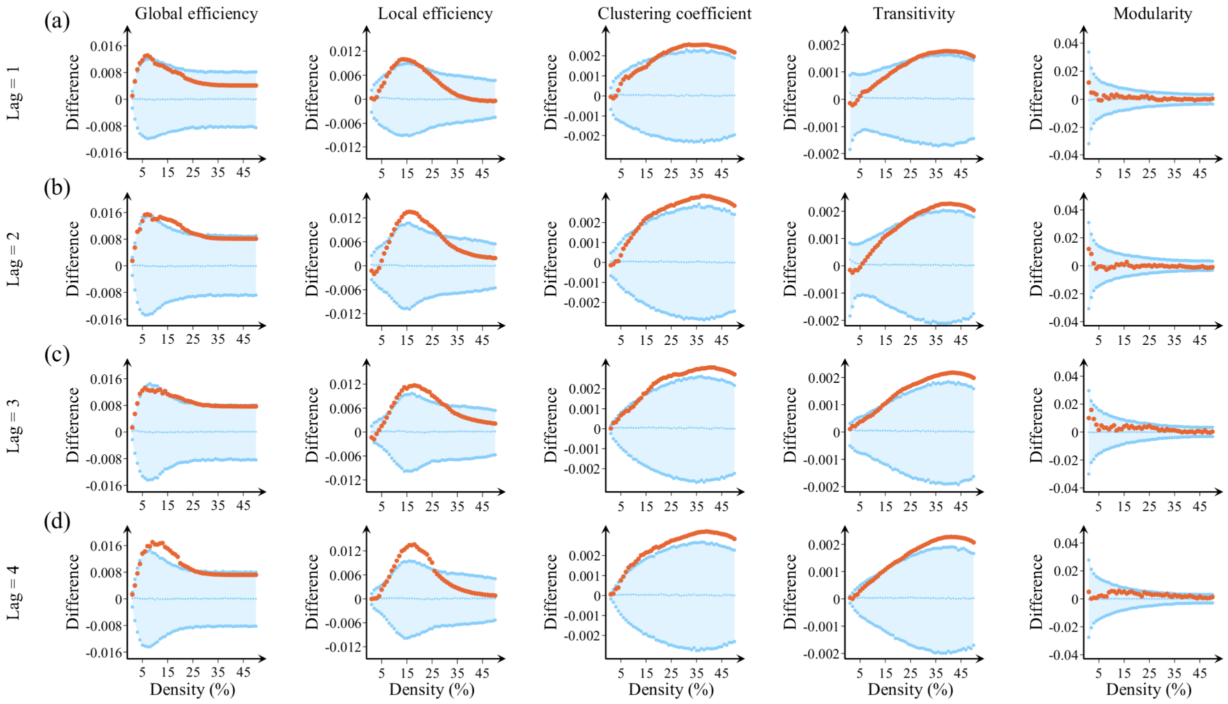

FIG. S22. **Differences between controls and participants with PD in global network measures.** Plots showing the differences between controls and participants with PD in the global efficiency, local efficiency, clustering coefficient, transitivity and modularity for lags of (a) 1, (b) 2, (c) 3 and (d) 4. The networks were calculated using the anti-symmetric correlations using an alternative thresholding method where the edge weights were retained after binarization. The plots show the upper and lower bounds of the 95% confidence intervals (CI) in blue, and the differences in the network measures between groups in orange circles as a function of network density. The differences are considered statistically significant if they fall outside the CIs.

B. Lags 5-7

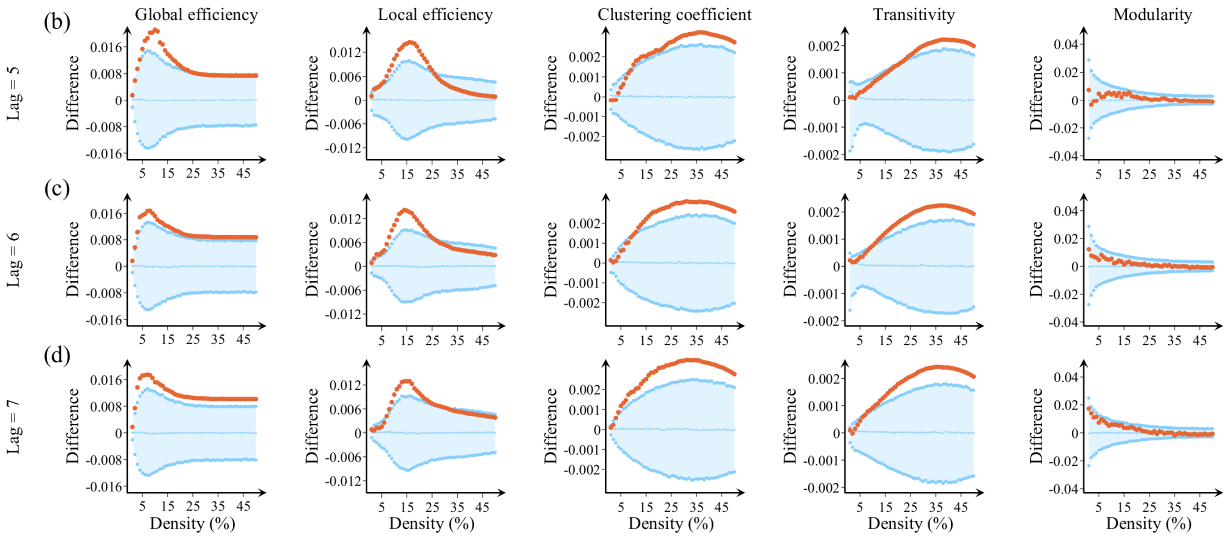

FIG. S23. **Differences between controls and participants with PD in global network measures.** Plots showing the differences between controls and participants with PD in the global efficiency, local efficiency, clustering coefficient, transitivity and modularity for lags of (a) 5, (b) 6 and (c) 7. The networks were calculated using the anti-symmetric correlations using an alternative thresholding method where the edge weights were retained after binarization. The plots show the upper and lower bounds of the 95% confidence intervals (CI) in blue, and the differences in the network measures between groups in orange circles as a function of network density. The differences are considered statistically significant if they fall outside the CIs.

X. WEIGHTED NETWORK ANALYSIS: DIFFERENCES IN NODAL TOPOLOGY

A. Lag 1

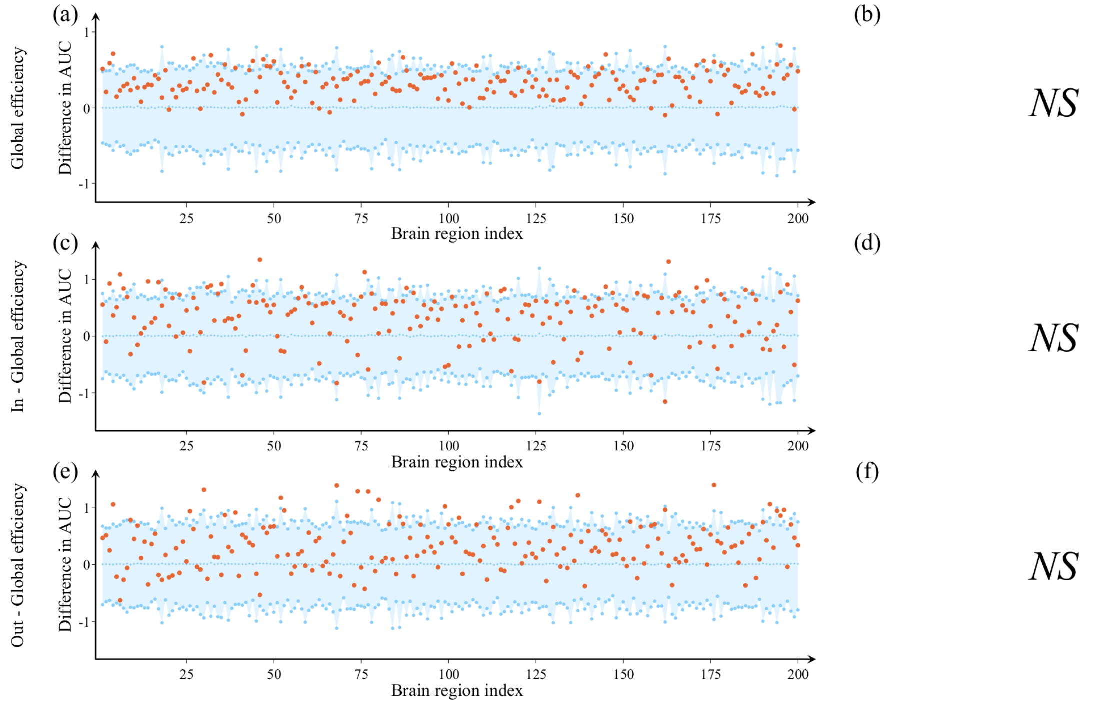

FIG. S24. **Differences between controls and participants with PD in nodal network measures using AUC analysis for lag 1.** Plots showing differences in the areas under the curve between controls and PD patients in the (a) global efficiency, (b) in - global efficiency and (c) out - global efficiency for all brain regions in the density range 1% - 50%. The plots show the upper and lower bounds of the 95% confidence intervals (CI) in blue, and the differences in the network measures between groups in orange circles as a function of network density. The differences are considered statistically significant if they fall outside the CIs. (b, d, f) Regions that showed significant between-group differences in the corresponding measures after correction for multiple comparisons (FDR, $q < 0.05$). NS: no brain regions remained significant after correction for multiple comparisons. The networks were calculated using the anti-symmetric correlations using an alternative thresholding method where the edge weights were retained after binarization.

B. Lag 2

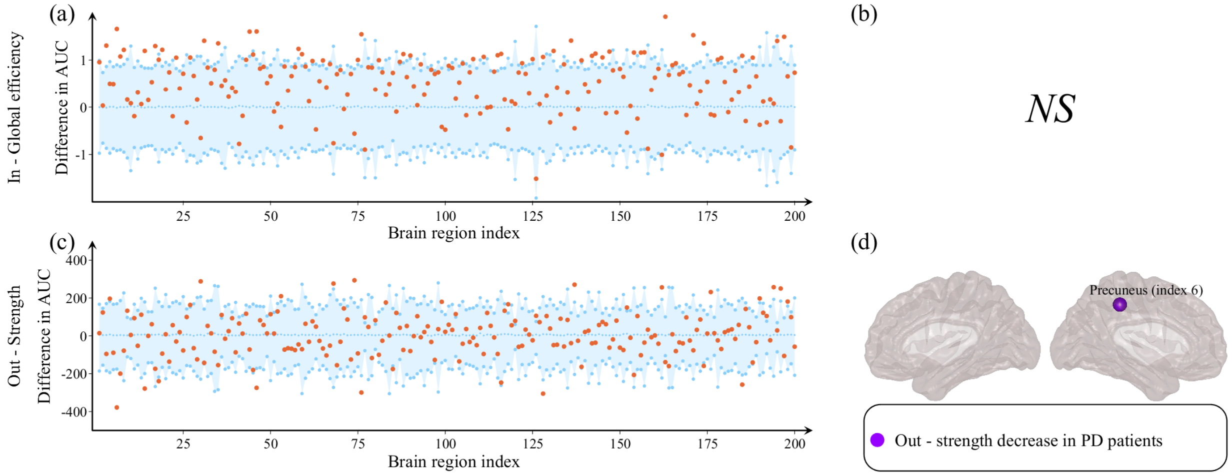

FIG. S25. **Differences between controls and participants with PD in nodal network measures using AUC analysis for lag 2.** Plots showing differences in the areas under the curve between controls and PD patients in the (a) in - global efficiency and (c) out - strength for all brain regions in the density range 1% - 50%. The plots show the upper and lower bounds of the 95% confidence intervals (CI) in blue, and the differences in the network measures between groups in orange circles as a function of network density. The differences are considered statistically significant if they fall outside the CIs. (b, d) Regions that showed significant between-group differences in the corresponding measures after correction for multiple comparisons (FDR, $q < 0.05$). NS: no brain regions remained significant after correction for multiple comparisons. The networks were calculated using the anti-symmetric correlations using an alternative thresholding method where the edge weights were retained after binarization.

C. Lag 3

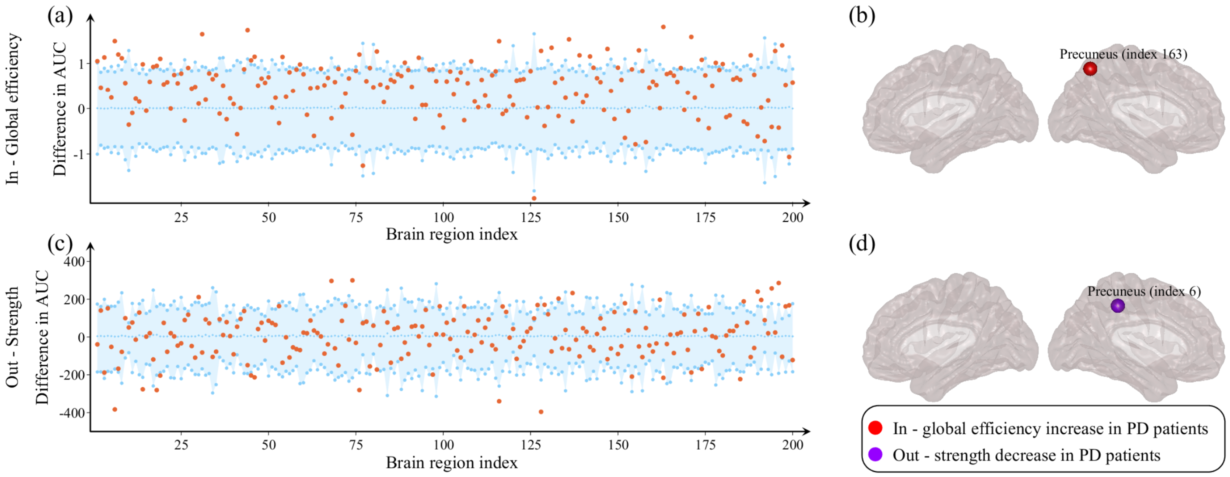

FIG. S26. **Differences between controls and PD patients in nodal network measures using AUC analysis for lag 3.** Plots showing differences in the areas under the curve between controls and participants with PD in the (a) out - strength for all brain regions in the density range 1% - 50%. The plots show the upper and lower bounds of the 95% confidence intervals (CI) in blue, and the differences in the network measures between groups in orange circles as a function of network density. The differences are considered statistically significant if they fall outside the CIs. (b) Regions that showed significant between-group differences in the corresponding measures after correction for multiple comparisons (FDR, $q < 0.05$). The networks were calculated using the anti-symmetric correlations using an alternative thresholding method where the edge weights were retained after binarization.

D. Lag 4

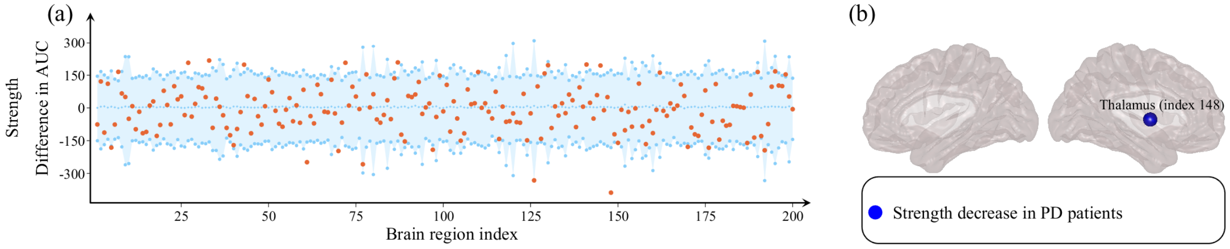

FIG. S27. **Differences between controls and PD patients in nodal network measures using AUC analysis for lag 4.** Plots showing differences in the areas under the curve between controls and participants with PD in the (a) strength for all brain regions in the density range 1% - 50%. The plots show the upper and lower bounds of the 95% confidence intervals (CI) in blue, and the differences in the network measures between groups in orange circles as a function of network density. The differences are considered statistically significant if they fall outside the CIs. (b) Regions that showed significant between-group differences in the corresponding measures after correction for multiple comparisons (FDR, $q < 0.05$). The networks were calculated using the anti-symmetric correlations using an alternative thresholding method where the edge weights were retained after binarization.

E. Lag 5

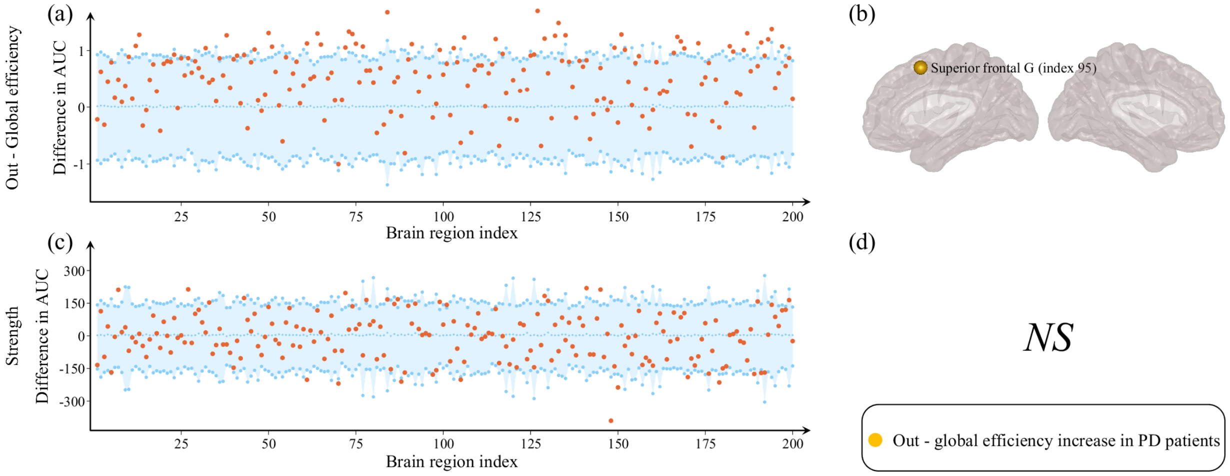

FIG. S28. **Differences between controls and PD patients in nodal network measures using AUC analysis for lag 5.** Plots showing differences in the areas under the curve between controls and participants with PD in the (a) out - global efficiency and (c) strength for all brain regions in the density range 1% - 50%. The plots show the upper and lower bounds of the 95% confidence intervals (CI) in blue, and the differences in the network measures between groups in orange circles as a function of network density. The differences are considered statistically significant if they fall outside the CIs. (b, d) Regions that showed significant between-group differences in the corresponding measures after correction for multiple comparisons (FDR, $q < 0.05$). NS: no brain regions remained significant after correction for multiple comparisons. The networks were calculated using the anti-symmetric correlations using an alternative thresholding method where the edge weights were retained after binarization.

F. Lag 7

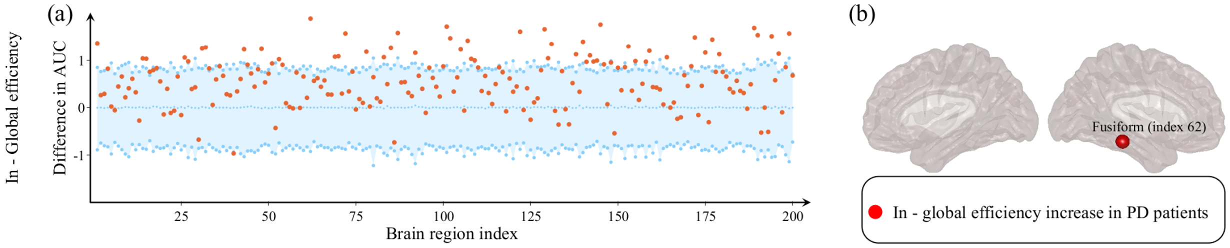

FIG. S29. **Differences between controls and PD patients in nodal network measures using AUC analysis for lag 7.** Plots showing differences in the areas under the curve between controls and PD patients in the (a) in - global efficiency for all brain regions in the density range 1% - 50%. The plots show the upper and lower bounds of the 95% confidence intervals (CI) in blue, and the differences in the network measures between groups in orange circles as a function of network density. The differences are considered statistically significant if they fall outside the CIs. (b) Regions that showed significant between-group differences in the corresponding measures after correction for multiple comparisons (FDR, $q < 0.05$).

XI. GRANGER CAUSALITY ANALYSIS

A. Lags 1-4

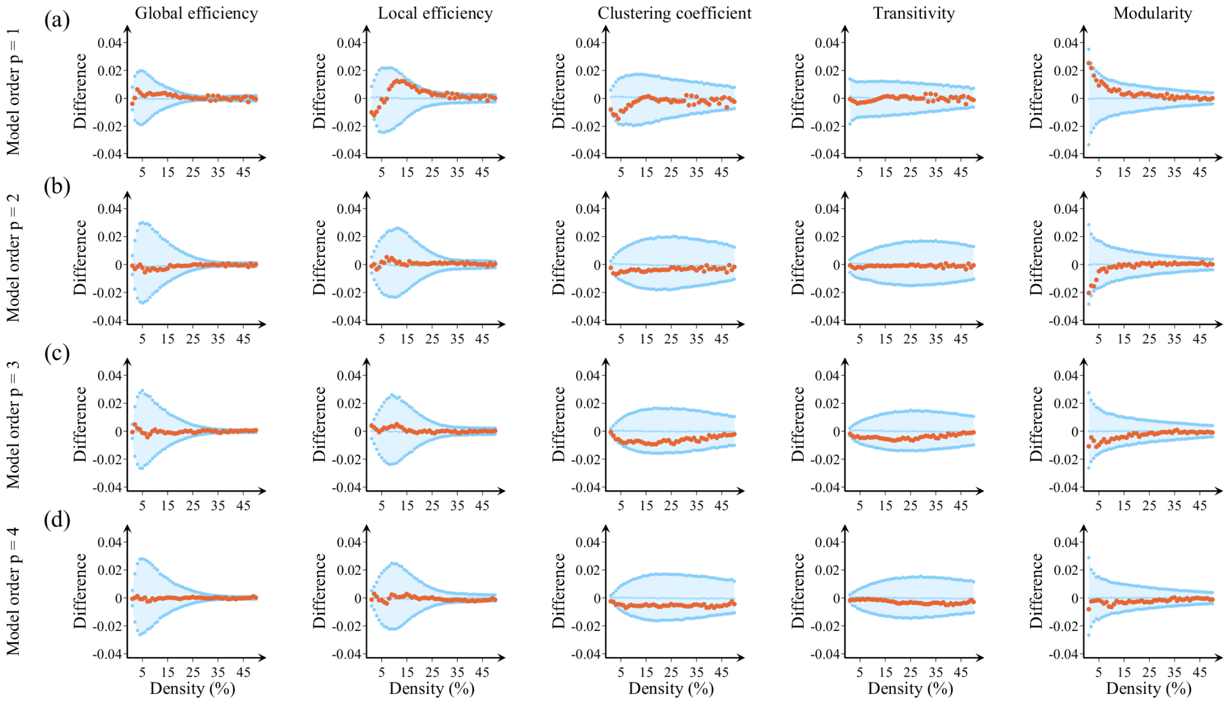

FIG. S30. **Differences between controls and PD patients in global network measures for networks derived by Granger Causality: Model orders 1-4.** Plots showing the differences between controls and PD patients in the global efficiency, local efficiency, clustering coefficient, transitivity and modularity for model order of (a) 1, (b) 2, (c) 3 and (d) 4, when the networks are calculated by the Granger Causality. The plots show the upper and lower bounds of the 95% confidence intervals (CI) in blue, and the differences in the network measures between groups in orange circles as a function of network density. The differences are considered statistically significant if they fall outside the CIs.

B. Lags 5-7

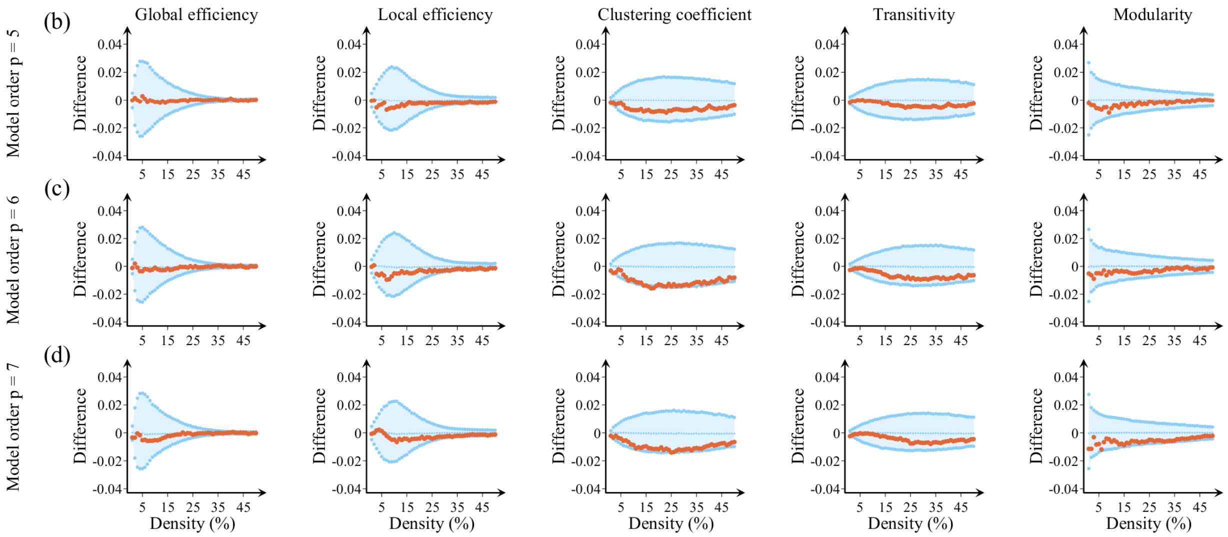

FIG. S31. **Differences between controls and PD patients in global network measures for networks derived by Granger Causality: Model orders 5-7.** Plots showing the differences between controls and PD patients in the global efficiency, local efficiency, clustering coefficient, transitivity and modularity for model order of (a) 5, (b) 6 and (c) 7, when the networks are calculated by the Granger Causality. The plots show the upper and lower bounds of the 95% confidence intervals (CI) in blue, and the differences in the network measures between groups in orange circles as a function of network density. The differences are considered statistically significant if they fall outside the CIs.
